# Cleavage and polyadenylation: Ending the message expands gene regulation

**DOI:** 10.1080/15476286.2017.1306171

**Published:** 2017-04-28

**Authors:** Jonathan Neve, Radhika Patel, Zhiqiao Wang, Alastair Louey, André Martin Furger

**Affiliations:** Department of Biochemistry, University of Oxford, Oxford, United Kingdom

**Keywords:** 3′end processing, alternative cleavage and polyadenylation, gene expression

## Abstract

Cleavage and polyadenylation (pA) is a fundamental step that is required for the maturation of primary protein encoding transcripts into functional mRNAs that can be exported from the nucleus and translated in the cytoplasm. 3′end processing is dependent on the assembly of a multiprotein processing complex on the pA signals that reside in the pre-mRNAs. Most eukaryotic genes have multiple pA signals, resulting in alternative cleavage and polyadenylation (APA), a widespread phenomenon that is important to establish cell state and cell type specific transcriptomes. Here, we review how pA sites are recognized and comprehensively summarize how APA is regulated and creates mRNA isoform profiles that are characteristic for cell types, tissues, cellular states and disease.

## Introduction

To express genes, the information present in the DNA blueprint is transcribed by RNA polymerases into RNA molecules. These initial or primary transcripts are often subjected to modifications and processing reactions that mature them into functional molecules. While the processing of some noncoding RNAs such as rRNA and tRNAs are also essential in prokaryotes, extensive processing of protein encoding pre-mRNA transcripts is a unique feature of eukaryotic gene expression.^1^

In eukaryotes, 3 major processing reactions are responsible for the maturation of pre-mRNA molecules into mRNAs that are competent for nuclear cytoplasmic export and subsequent translation.^2^ Firstly, the 5′ends of RNAs are modified by the addition of a cap structure in the form of a guanosine molecule that is enzymatically attached to the first nucleotide of the transcribed mRNA by an unusual 5′–5′ triphosphate linkage and subsequently modified by methylation. The genes that encode proteins are mostly not continuous, but separated into alternating exons (coding regions) and introns, which are noncoding regions that need to be removed. This occurs during splicing, where a large multi-protein-RNA complex, known as the spliceosome, directs precise excision of introns from the primary transcript and fuses the exons together. This generates an mRNA with a continuous open reading frame (ORF) that serves as a template for protein synthesis in the cytoplasm. In order for the transcript to be exported to the cytoplasm, the transcript needs to be detached from RNA Polymerase, which occurs by a 2-step cleavage and polyadenylation reaction. This forms the typical uniform polyadenylated 3′end of almost all of the protein encoding nuclear mRNAs.

While splicing has long been recognized to play an important role in the regulation of gene expression in eukaryotes, the scope for gene regulation at the level of cleavage and polyadenylation has only recently been fully recognized.

## Core cleavage and polyadenylation sequence elements

**Figure 1.**
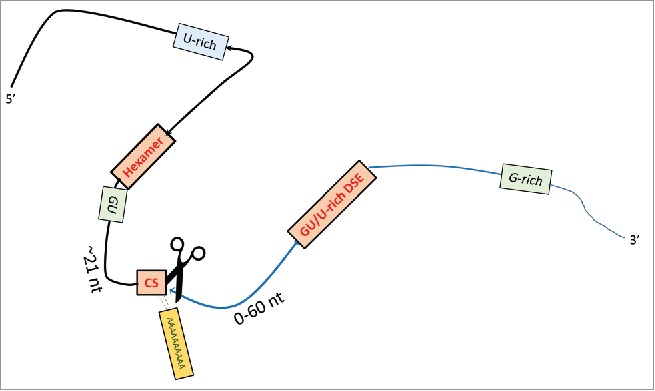
The cis-elements that define pA sites. The cleavage and polyadenylation machinery relies on key cis elements to mediate 3′end processing. Canonical cis elements include the A[A/U]UAAA hexamer and its variants which lie ∼21 nucleotides upstream of the cleavage site (CS) and a downstream less well defined GU/U-rich element. Additional auxiliary elements may be positioned upstream and/or downstream of the cleavage site and are often U, GU and or G-rich.

With the exception of replication dependent histone genes, metazoan protein encoding mRNAs contain a uniform 3′end consisting of a stretch of adenosines. This poly(A) tail is synthesized in a template independent fashion and is the result of an RNA processing reaction, where a multiprotein complex assembles on specific sequences on the pre-mRNAs, called the cleavage and polyadenylation signals (pA signals). pA signals consist of sequences that flank either side of where the pre-mRNA is endonucleolytically cleaved and subsequently polyadenylated (Fig. 1). The core pA signal is a bipartite sequence element that constitutes an A-rich hexameric sequence, found 21 nucleotides upstream of the cleavage site^3^ and a U and/or G/U rich sequence, located 10–30 nucleotides downstream of the cleavage site. Whereas the upstream hexamer motif for most (70–75%) pA signals conforms to the canonical A[A/U]UAAA hexamer,^4,5^ the nucleotide composition of the downstream sequence element (DSE) can vary significantly between pA signals. Although no clear consensus can be defined,^6^ often a GU rich sequence followed by a stretch of 3 or more uridines is present within a 40 nucleotide wide window downstream of the cleavage site.^7^

### Core sequences upstream of the cleavage site

The importance of the nucleotide composition in the hexamer sequence element is underpinned by numerous *in vivo* and *in vitro* experimental examples. Single nucleotide changes in the A[A/U]UAAA sequence, or its entire removal,^8-10^ have proven to severely impair cleavage efficiency. These laboratory based observations are further strengthened by the naturally occurring polymorphisms such as found in the human globin genes,^11-14^ which are associated with the thalassemia phenotype. However, these single nucleotide polymorphisms (SNPs) rarely occur in the pA hexamer,^15^ further emphasizing the importance of the hexamer nucleotide composition. However, naturally occurring **non-canonical variants**, with a deviation of one or more nucleotides from the A[A/U]UAAA hexamer sequence consensus, represent about 10-20% of all hexamer signals.^4,5,12-14,16^

### Core sequences downstream of the cleavage site

In contrast to the hexamer, the DSE has long been considered to be much more tolerant to single nucleotide changes.^17^ However, at least for a subset of genes, in particular those with degenerated hexamer sequences, the opposite is true: while mutations in the hexamer had little effect, base changes in the DSE reduced 3′end processing significantly.^18^ That the DSE can be a decisive element in regulating efficiency of cleavage is further supported by clinically relevant examples where changes in the DSE force a gain of function and increase the processing capacity of a particular pA site.^12,19^ In addition, knockdown of CstF64, the component of the 3′end processing machinery known to interact with the DSE (see below), affects usage of pA sites that are characterized by a non-canonical hexamer and a GUKKU “type” DSE.^20^ Furthermore, for a subset of genes that have pA sites with degenerated DSEs, CstF64 is not required for 3′end processing. The mRNAs of these genes are polyadenylated by Star-PAP, a non-canonical poly(A) polymerase.^21^ These observations suggest that for some pA sites, the DSE may represent the critical core element that drives cleavage efficiency and directs how the 3′end machinery is assembled at the pA site.

### The cleavage site

The point of cleavage occurs between the hexamer and DSE. In mammals, CA and UA appear to be the most frequent dinucleotides that precede the actual site of cleavage,^22^ however, the actual site where cleavage occurs is known to be heterogeneous. Nucleotide composition at the cleavage site has a complex impact on 3′end processing. Initial *in vitro* based studies revealed that mutations of the dinucleotides at the cleavage site generally resulted in relatively moderate effects on cleavage efficiency. Conversely, a prominent SNP occurring at a 1% frequency in the Caucasian population, altering the cleavage site of the prothrombin gene from CG to CA, significantly increases cleavage efficiency of the pre-mRNA, and has been linked to a higher tendency toward thrombophilia.^23^

### Auxiliary elements

In addition to the core sequence elements, many pA sites possess additional auxiliary sequences that influence their overall strength. These are diverse in location and nucleotide composition, but in most cases feature U/G rich sequences.^7^ The presence of these auxiliary elements correlates with deviation from the consensus A[A/U]UAAA hexamer, perhaps compensating for the loss of core sequence integrity.^18^ These “processing enhancers” can be found both upstream and downstream of the cleavage sites.

G-rich auxiliary sequences are some of the best characterized downstream enhancers that may fold into G-quadruplex structures. Several viral and mammalian pA sites have been described where these G-rich sequences significantly influence cleavage efficiency at the pA site^6^ by interacting with factors, such as hnRNPH, that stimulate processing.^24,25^ Because the G-rich sequences can exert their influence over distances that span several hundred bases,^24,26,27^ the true extent to which pA sites depend on these distant enhancers may be undervalued.

U-rich auxiliary regions are frequently found upstream and downstream of the cleavage site.^3,7^ The upstream U-rich sequences can be divided into 2 categories. The first category represents U-rich elements that are located between the hexamer and the actual cleavage site.^7,28^ They can function as binding sites for the CPSF subunit Fip1, which can stimulate the polyadenylation step^29^ and control pA site selection.^30,31^ The second category includes U-rich sequences that are positioned in the UTR upstream of the hexamer. These upstream elements (USEs) were first identified in several viral transcripts including SV40,^32^ Cauliflower Mosaic Virus (CaMV)^33^ and HIV-1^34^ In the HIV-1 transcript the USE, identified between 56 and 93 nucleotides upstream of the hexamer, was found to interact with CPSF160 and aid its interaction with the poly(A) signal, proving to be critical for the cleavage reaction.^35^ USEs generally have no consensus sequences, although tend to be U-rich.^17^ A number of USEs have now been identified in transcripts of the human genome. In the lamin B2 and C2 complement genes, auxiliary USEs are required for interaction with PTB and CstF64 respectively, which is critical for the cleavage and polyadenylation reaction.^36,37^ A USE controls the usage of the poly(A) sites in the prothrombin gene in a stress response,^38^ and efficiency of polyadenylation in collagen genes has also been shown to be modulated by USEs.^39^ In addition, the usage of the proximal poly(A) site of the cyclo-oxygenase 2 gene is controlled by 3 U-rich USEs that bind a protein complex to recruit the core polyadenylation factors.^40,41^

A UGUA motif has been identified as a USE, which is bound by the cleavage factor I (CFI) complex. UGUA mediated CFIm recruitment to the pre-mRNA can direct cleavage and polyadenylation at non-canonical sites, by anchoring Fip1 and the poly(A) polymerase (PAP) to the pre-mRNA.^42,43^ More recent whole transcriptome based analyses identified a more global role for this interaction and recognized CFI as a key regulator of pA site choice during alternative cleavage and polyadenylation (APA) for a significant number of genes.^44,45^ Finally, some of the U-rich elements have also been shown to mediate an interaction with hnRNPC similarly influencing pA site selection during APA.^3^

AUA auxiliary element. A subset of mRNAs are polyadenylated by the non-canonical poly(A) polymerase Star-PAP (see below), which binds an AUA motif in the 3′UTR of its target pre-mRNAs.^21^

## The core poly(A) machinery

**Figure 2.**
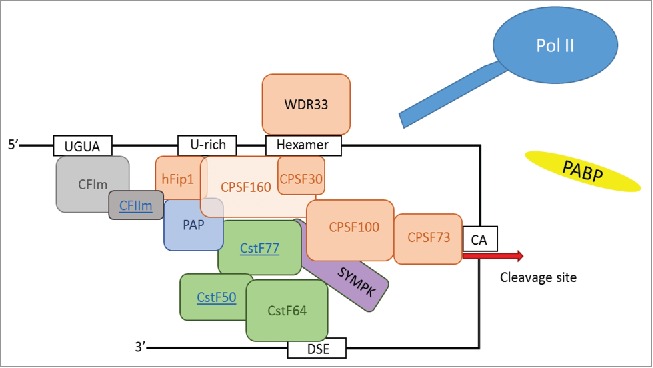
The core factors of the cleavage and polyadenylation complex. There are more than 80 proteins associated with the cleavage and polyadenylation machinery but fewer than 20 factors are considered to build the core of the processing complex.^47^ The major components are made up of multi-subunit factors including the cleavage and polyadenylation specificity factor CPSF (WDR33, hFip1, CPSF160, CPSF100, CPSF70, CPSF30); the cleavage stimulatory factor CstF (CstF77, CstF64, CstF50), the CFI (CFIm65, CFIm25) and CFII (∼15 subunits). The core factors involved in cleavage and polyadenylation, and the cis elements to which they bind are outlined here. Details of the individual factors are given in the text.

In mammals there are more than 80 proteins associated with the 3′end processing machinery,^46^ but this can be condensed to around 20 proteins that constitute the core factors^47^ (Fig. 2). Part of this core machinery includes 4 multi-subunit protein complexes which are highly conserved: the cleavage and polyadenylation specificity factor (CPSF), the cleavage stimulation factor (CstF), and mammalian cleavage factors I and II (CFIm and CFIIm). Additional core factors include symplekin, the poly(A) polymerase (PAP), nuclear poly(A) binding protein (PABPN1) and the C-terminal domain (CTD) of the largest subunit of RNA polymerase II (Pol II). The process of 3′end formation is initiated by the recognition of the respective *cis*-elements on the nascent transcripts by CPSF and CstF complexes in a cooperative manner.^48^ Depending on the pA site, this initial step is supported and often dependent on the interaction of other core factors (for example CFI)^43^ and also auxiliary factors (for example hnRNP H).^24^ After a functional complex is assembled cleavage and subsequent polyadenylation of the pre-mRNA can commence.

### CPSF

The CPSF protein complex features 6 protein subunits; WDR33, CPSF30, CPSF160, hFip1, CPSF100 and CPSF73, which are required for cleavage of the pre-mRNA. In contrast, only 4 CPSF subunits, CPSF160, CPSF30, WDR33 and hFip1 together with PAP are sufficient to direct AAUAAA dependent poly(A) tail addition *in vitro*.^49^ The different dependencies on the CPSF subunits raises the intriguing possibility that different sub-complexes may be specific for the cleavage or the polyadenylation steps.^49^ This idea is supported by the finding that different CPSF sub-complexes have been identified that cater either for pA or histone 3′end processing.^50^

**WDR33 *(WDR33)*** is a large 146 kDa protein, and despite the essential role its homolog Pfs2p plays in 3′end formation in yeast,^51^ its role during mammalian cleavage and polyadenylation has only recently been uncovered. The mammalian WDR33 was first shown to be essential in cleavage and polyadenylation *in vitro*,^52^ and has subsequently been identified as the CPSF subunit that directly binds the AAUAAA hexamer.^49,53^ Its importance for pA site recognition is further underpinned by PAR-CLIP data, which shows a strong interaction between WDR33 and the pre-mRNA directly upstream (−16 to −18 nt) of cleavage sites,^49^ close to the region where hexamers peak in transcripts.^3^ It is currently unclear how its specific interaction with AAUAAA is achieved, but the WD40 domain, which is principally associated with protein-protein interactions, also has RNA-binding properties.^54^

**CPSF30 *(CPSF4)*** is the smallest subunit of the CPSF complex. The involvement of a 30 kDa protein in the recognition of AAUAAA has long been recognized,^55^ but it took more than 25 years to show that this protein, together with WDR33, is responsible for the specific interaction of CPSF with the AAUAAA hexamer in mammals.^53^ Interestingly, CPSF30 has also been shown to play a central role in pA site selection in plants.^56,57^ The RNA-binding properties of CPSF30 reside in the 5 zinc finger (ZF) domains, primarily mediated by ZF2 and ZF3.^53,58^ The binding of CPSF30 to RNA is required for the entire CPSF complex to interact with the RNA substrate.^53,59^ Notably, the CPSF30-AAUAAA interaction is specifically inhibited by the influenza protein NS1A, that blocks ZF2 and ZF3, and so globally abrogates host 3′end processing.^60^ These observations clearly highlight the central role of CPSF30 in pA site recognition.

**CPSF160 *(CPSF1)*** was considered to be the major component that mediates the binding of the CPSF complex to AAUAAA. This was primarily based on the observation that a 160 kDa protein can be specifically cross linked to AAUAAA-containing RNA substrates^61^ and the Yhh1/Cft1p, the yeast homolog of CPSF160, also bound RNA.^62^ Furthermore, pull-down assays confirmed that recombinant CPSF160 interacts with AAUAAA-containing RNAs. However, as mentioned above, it is now clear that AAUAAA-specific binding of the CPSF complex falls to WDR33 and CPSF30 and thus the precise role of CPSF160 for the interaction with the pre-mRNA needs to be re-evaluated. Nevertheless, CPSF160 is a critical component of the cleavage and polyadenylation machinery and establishes important protein-protein interactions. These include interactions between CPSF and CstF by directly contacting the CstF77 subunit. This interaction may be integral to the cooperative nature of pA site recognition. In addition, CPSF160 also interacts functionally with the PAP.^63^

**hFip1 (*FIP1L1*)**, the human factor interacting with PAP, was discovered based on the sequence similarity to the yeast polyadenylation factor Fip1p. True to its name, Fip1, and hFip1, interact with and recruit PAP to the pA site where it can simultaneously bind to U-rich regions that are often located either upstream or downstream of the pA hexamer. The RNA-binding activity of hFip1 lies within the arginine-rich C-terminus.^29^ A role in pA site recognition is further supported by the fact that hFip1 levels change in stem cells compared with differentiated cells. This directs APA during stem cell self-renewal. Higher levels of hFip1 in ESCs and iPSCs promote the recognition of weaker, hFip1 dependent pA sites.^30^

**CPSF100 *(CPSF2)* and CPSF73 *(CPSF3)***, which both contain metallo-β-lactamase and β-CASP domains, form a heterodimer in mammals.^64^ The precise role of CPSF100 for cleavage and polyadenylation in mammals is still unclear. There is however evidence that CPSF100 is involved in THOC5 (a member of the TREX export complex) mediated control of 3′end processing of immediate early genes. THOC5 recruits CPSF100 to the 3′end of immediate early genes that are regulated by THOC5.^65^ The CASP domain in CPSF73 provides the zinc-dependent endonuclease activity during 3′end formation for both the cleavage and polyadenylation^66^ and the histone 3′end processing machineries.^67^ While they play a central role during cleavage, both proteins are superfluous for the polyadenylation step.

### CstF

**CstF** is the component of the cleavage and polyadenylation machinery that specifically recognizes the DSE region in pA sites. It is a heterotrimeric protein complex containing the subunits CstF77, CstF64 and CstF50.^48,68^ The CstF complex is essential for the cleavage reaction but is not required for the polyadenylation step.^69,70^

**CstF64 (*CSTF2*)** mediates the binding to the DSE in the pre-mRNA.^71^ The interaction interface resides in an RNA recognition motif (RRM) at its N-terminus, which specifically recognizes U/GU-rich regions downstream of the cleavage sites. How CstF64 is able to recognize diverse DSEs in pre-mRNAs remains uncertain, but consecutive Us are required for strong CstF-GU interactions. Interestingly, RNA binding also induces structural changes in CstF64 that may be critical to facilitate assembly of larger complexes.^72^ A global analysis that mapped transcriptome wide CstF64-RNA interactions *in vivo* suggests a role for CstF64 in modulating the selection of pA sites during APA.^73^ This promotes the usage of non-canonical pA sites that feature a GUKKU motif in their DSE.^20^

**CstF77 *(CSTF3)*** bridges the CstF64 and CstF50 subunits^74^ and thus plays a key role for the assembly of the CstF complex. CstF77 appears to be an elongated dimer, suggesting that CstF may form a hexomeric complex in cells.^75^ CstF77 also appears to be critical for the establishment of protein interactions between CstF and other pA complexes. Cross linking experiments demonstrate a strong interaction between CPSF160 and CstF77, which may provide the basis for the observed CPSF-CstF cooperative RNA binding during the assembly process.^63^

**CstF50 *(CSTF1)***, like CstF77, exists as a homodimer^76^ providing further evidence for the hexameric nature of functional CstF complexes. CstF50 contains 7 repeats of the WD40 motif, which mediates protein-protein interactions with CstF77 and BRCA1 associated RING domain 1 (BARD1).^77^ The latter interaction is inhibitory and believed to suppress erroneous 3′end formation possibly during transcription coupled DNA repair.^78^ CstF50 also interacts with the CTD of Pol II and this interaction may be established via the N-terminal part of CstF50.^79^

**CFIm** is a complex is made up of 4 polypeptides: **CFIm25 *(CPSF5/NUDT21)*, CFIm68 *(CPSF6)*, CFIm59 *(CPSF7)*** and **CFIm72 (alternative spliced isoform of CFIm68*)***. It has been suggested that different forms of CFIm complex exist *in vivo* including CFIm25-68, CFIm25-59 and possibly CFIm25-72. As shown by SELEX experiments^42^ and site directed mutagenesis,^43^ CFIm recognizes a UGUAN motif in RNA through the RRM in CFIm25. The RRM in CFIm68 is critical for the interaction with CFIm25 and enhances RNA-binding of the complex. CFIm68 also facilitates RNA looping, which may play a role in the regulation of APA.^80,81^ CFIm has originally been shown to stabilize the binding of CPSF complex to the pre-mRNA^82^ and then emerged as a critical factor for the recognition of pA sites that lack the A[A/U]UAAA hexamer.^43^ To date, CFI is arguably the best characterized factor that regulates APA at the point of cleavage. This is demonstrated by numerous examples where a depletion of CFIm results in the preferred usage of the proximal pA sites.^20,44,45,83^ CFIm has also been implicated in regulating alternative usage of non-canonical pA sites during spermatogenesis.^84^ There are also contacts between CFIm68 and THOC5, a component of the transcription export complex (TREX). THOC5 appears to be important for the recruitment of CFIm68 to distal pA sites.^85^

**CFIIm**, mammalian CFII, is only required for the cleavage step and is the least well characterized complex of the core component. To date, this complex has only been partly purified and can be separated into 2 fractions: CFIIAm and CFIIBm. The CFIIAm fraction contains the essential components for the cleavage reaction while CFIIBm is not required but has a stimulatory role for cleavage. One of the 15 polypeptides associated with CFIIAm, **hClp1** has been shown to interact with CFIm and CPSF by immunoprecipitation experiments, suggesting that it bridges these 2 during the cleavage process.^86^ An additional component of CFII is protein 1 of cleavage factor 1, **hPcf11**, which has been shown to be critical in the control of Pol II transcription termination, by mediating the degradation of the polymerase associated 3′ RNA product after cleavage at the pA site.^87^ More recently, hPcf11 has also been implicated in regulating APA.^31^

**PAP (*PAPOLA*)** is a monomer and responsible for the addition of the 3′ polyadenosine tail to a newly synthesized pre-mRNA molecule^88^ by catalyzing the chemical reaction ATP + RNA-3′OH → pyrophosphate + RNApA-3′OH.^89,90^ PAP adds 200–250 adenosines in a template-independent manner to the 3′end of the cleaved pre-mRNA. PAP is recruited to the 3′end processing complex by CPSF through interactions mediated by CPSF160 and hFip1.^29^ There are several PAP isoforms present in human cells which can chiefly be separated into canonical PAPs and non-canonical PAPs. Most RNAs are polyadenylated by canonical PAPs. Non-canonical PAPs, among other functions, are associated with mitochondrial mRNA polyadenylation (hmtPAP), cytoplasmic polyadenylation (hGld2) and miRNA biogenesis. A prominent non-canonical PAP called Star-PAP, controls the polyadenylation of a subset of mRNAs that encode for proteins that are associated with DNA damage induced apoptosis and stress responses.^91^

**PABPN1 (*PABPN1*)** has been shown to contain a single RRM and an arginine rich CTD.^92^ The rate at which PAP adds adenosine nucleotides is dependent on the presence of PABPN1. The first few nucleotides added by PAP are added with low efficiency until the short polyadenosine tail is bound by PABPN1, which accelerates the rate of adenosine addition by PAP.^93^ While PAP adds 200–250 adenosine nucleotides to the 3′end of the mRNAs,^94^ the median length of the poly(A) tail in cellular mRNAs is much shorter, between 50 to 100 nucleotides long due to the action of cytoplasmic deadenylases.^95^ In addition to its primary function, PABPN1 has also been implicated in controlling APA.^96,97^

**Symplekin** (***SYMPK***) is a protein that forms a high-molecular weight complex with CPSF and CstF by interacting with CPSF73, CPSF100 and CstF64. It is suggested to serve as a scaffold for recruiting other factors to the cleavage and polyadenylation complex.^98^ It also participates in the assembly of a processing complex that matures histone mRNA 3′ends, which do not undergo polyadenylation. Symplekin has also been found to form a complex with heat shock transcription factor 1 (HSF1) after stress treatment. This complex may be critical for the recruitment of the 3′end processing machinery to the heat shock protein *HSB* pre-mRNA during stress and so safeguard its expression.^99^ Symplekin appears to provide a scaffold around which different CPSF sub-complexes are assembled. Perhaps these different sub-complexes provide the necessary scope and flexibility to the 3′end machinery that is required to associate with pA sequences that differ widely in their architecture.

**Pol II CTD (*RBP1*)** is the key platform that couples pre-mRNA processing to transcription and it is required for efficient cleavage at the pA site.^100^ The human Pol II CTD features 21 consensus YSPTSPS and 31 non-consensus heptad repeats^101^ which form and present a dynamic interaction platform for a variety of pre-mRNA processing components including cleavage and polyadenylation factors such as Pcf11, CstF77 and CstF50.

## The assembly of a functional cleavage and polyadenylation complex

The initiating step of the 3′end processing complex assembly (Fig. 2) is the coordinated recognition of the hexamer signal AAUAAA and the DSE on the nascent RNA by CPSF and CstF. The recruitment of CPSF is central, as it constitutes the core processing complex required for both the cleavage and the subsequent polyadenylation reaction. It binds the RNA directly by the association of WDR33 and CPSF30 with the hexamer. Whether the 2 subunits bind to pre-mRNAs concurrently or in a time and/or pA site type dependent manner remains unknown. It may well be that different CPSF complexes provide a flexible machinery that can recognize different types of pA signals. Unlike CPSF, the CstF complex is essential only for the cleavage reaction. CstF associates with di-uridine pockets in the U/GU rich DSE via its RRM in CstF64.^71^ CstF77 is a critical component that bridges the CPSF and CstF components, facilitating the cooperative binding.^63^ With CPSF and CstF anchored to the pA site, additional factors join and complete the assembly of a functional 3′end processing complex. The complex can then catalyze a 2-step reaction comprising of an endonucleolyic cleavage at the cleavage site and the polyadenylation of the 3′end of the 5′cleavage product. RNA cleavage occurs between the hexamer and the DSE, generally within a window of 20 nucleotides, either side of these core sequences.

The speed of assembly of a functional cleavage and polyadenylation complex is dependent on the strength of the pA site, but is generally achieved within about 10 seconds.^102^ The absolute strength of a given pA site is highly complex and depends on the sequence architecture of the pA site (see above) and the relative availability of core and auxiliary promoting or inhibitory factors.

## Interconnection between pA cleavage, capping and splicing

The assembly process of the functional machinery is further complicated by the interconnection between 3′end processing with capping, pre-mRNA splicing and transcription. The relationship between capping and 3′end formation was first suggested after capped RNAs proved to be better substrates for 3′end processing in nuclear extracts.^103^ These initial observations were confirmed and shown to be mediated by the cap binding complex, CBC.^104^

The relationship between splicing and 3′end formation is extensive and principally relies on direct interactions between splicing and cleavage and polyadenylation components. The positive influence of splicing factors on 3′end formation was first reported over 25 years ago and has since been extensively documented. Intron insertion into a replication dependent histone gene reporter is one example of this interconnection, where a splicing event in the histone pre-mRNA is concomitant with the activation of cryptic pA sites. As a result, cleavage and polyadenylation in the spliced transcripts is favored over U7 snRNP dependent 3′end formation,^105^ which is typical for replication dependent histone genes (reviewed in Romeo and Schümperli, 2016).^106^ Splicing has not only been shown to activate cryptic pA sites but generally stimulates cleavage and polyadenylation of the downstream pA sites.^107-110^ This stimulation is achieved by interactions between components of the splicing machinery that associate with the 3' splice sites (3SS) and several poly(A) factors notably between the 65 kDa subunit of U2AF, PAP and CFIm59.^111-113^ In addition, physical interactions of CPSF components with several subunits of the U2 snRNP, including SF3b155, SF3b130 and SF3b49 have been found.^114^ Correspondingly, mutations of U2 snRNA-binding sites significantly reduced the cleavage efficiency in reporter genes. The stimulatory effect through these interactions is mutual as mutations of the hexamer, can inhibit terminal intron removal^109^ and the depletion of CPSF100 impaired upstream splicing in an *in vitro* coupled splicing and 3′ processing system.^114^ CPSF and symplekin have been implicated in the promotion of alternative splicing on a global scale^115^ which further emphasizes the close connections of splicing and 3′end formation.

While splicing components associated with the 3SS appear to stimulate 3′end formation, the opposite is generally true for splicing components that bind to the 5SS, notably the U1 snRNP. The suppression of pA sites by U1 snRNP has proven to be instrumental for the inhibition of late gene expression in the bovine papilloma type 1 virus^116-118^ and in HIV-1, where the U1 snRNP bound to 5SS acts as a suppressor of upstream pA site usage.^119,120^ In the latter example, suppression of the upstream pA site is crucial to enable transcription of viral protein-encoding mRNA and the genomic RNA. This U1 snRNP-mediated type of suppression of 3′end formation is not restricted to viruses but was suggested to prevent premature transcription termination in polycistronic transcription units at a global scale in *Caenorhabditis elegans* by suppressing the recognition of the pA sites in upstream positioned genes in polycistronic mRNAs by Pol II associated poly(A) factors.^121^ Importantly, the suppressive role of U1 snRNP for 3′end formation emerged as a general mechanism by which cryptic pA sites in eukaryotic introns are suppressed globally, thereby protecting the cell from aberrant pA usage and premature transcription termination.^122^ Physiologically, U1 snRNP levels are very high which may ensure that levels never fall below a critical threshold that would result in the activation of these cryptic pA sites.^123^ However, U1 snRNP levels in cells can fluctuate, for example as a result of UV-induced DNA damage which causes increased usage of intronic alternative pA sites.^124^

## The polyadenylation step

The second part of the reaction comprises of the non-templated addition of about 200 adenosines,^93,94^ which in reconstituted systems only requires the cleaved pre-mRNA template, CPSF, PAP and the poly(A) binding protein (PABPN1, PABPII).^59^ PAP is initially tethered to the cleaved pre-mRNA via interactions with the CPSF subunits 160 kDa and Fip1.^29,63^ This loose interaction leads to regular dissociation of PAP and adenylation is restricted to intermittent addition of a few adenosines.^59^ Subsequently, the nuclear poly(A) binding protein, PABPN1, binds these short oligoadenosine tails added by PAP in its distributive mode.^93^ PABPN1-binding provides an additional anchor point for PAP and increases its affinity to the RNA 80-fold.^125^ Once this quaternary complex is established on the cleaved transcript, CPSF and PABPN1 cooperatively shift PAP into a processive mode, enabling it to catalyze the full length poly(A) tail without dissociation. The actual number of adenosines added to transcripts is uniform within species, ranging from 70 to 90 in *Saccharomyces cerevisiae*^126^ to around 200 in mammals. Extending the poly(A) tail to an appropriate length appears to be critical for appropriate gene expression, as both hypo- and hyperadenylation negatively influence the fate of the mRNA. Hypoadenylated mammalian transcripts have been shown to be retained in the nucleus,^127,128^ and inefficient polyadenylation in yeast is linked to nuclear mRNA degradation.^129^ In addition, hyperadenylated host transcripts observed in herpesvirus-infected cells are retained in the nucleus and funnelled into the nuclear mRNA degradation pathway.^130^ Furthermore, inhibition of RNA export forces hyperadenylation of nuclear mRNAs.^131^

How the precise length of poly(A) tail synthesis is achieved by the polyadenylation complex has been the subject of intensive investigations and much has been learned from reconstituted *in vitro* systems. Central to the model derived from these observations is that the decoration of the poly(A) tail with PABPN1 provides some means of measuring and controlling the number of adenosines added to the 3′end of the cleaved mRNA. The current model envisages that the growing poly(A) tail coated with PABPN1 folds into a spherical complex that promotes contacts between CPSF, PAP and PABPN1. When the tail extends to 250 adenosines, new PABPN1 proteins can no longer be integrated into the sphere and this leads to the disruption of the interactions between CPSF and PAP. Subsequently PAP remains tethered to the RNA only by its interaction with PABPN1 causing it to readopt the distributive mode and ultimately terminate polyadenylation.^94^ A direct involvement of PABPN1 in poly(A) length control is further supported by *in vivo* evidence. siRNA mediated depletion of PABPN1 in mouse myoblasts triggered shortening of poly(A) tails and subsequent accumulation of these transcripts in the nucleus.^128^ Similarly, nuclear retained transcripts with shortened adenosine tails are characteristic for influenza A virus infected cells. The viral NS1 protein appears to restrict the length of poly(A) tails on host transcripts in infected cells to 12 nucleotides by sequestering PABPN1.^127^

While CPSF, PAP and PABPN1 are sufficient to direct poly(A) tail length control in reconstituted systems, it appears that *in vivo*, more factors are required. The protein nucleophosmin (NPM1) has been found to associate with many mRNAs only after proper termination of polyadenylation.^132^ Intriguingly, RNAi knockdown of NPM1 leads to hyperadenylation and nuclear retention of mRNAs suggesting a role of NPM1 in poly(A) tail measurement. The precise molecular mechanisms are not clear but NPM1 somehow contributes to poly(A) tail sizing by regulating the dissociation of the quaternary complex.^132^ Perhaps NPM1 functions as a gatekeeper and associates with correctly polyadenylated and export competent transcripts.^133^

### Alternative cleavage and polyadenylation

The complexity of the pA site architecture combined with the multitude of core and auxiliary interacting factors and interconnection with splicing and transcription provides ample opportunities to regulate pA site recognition. It is therefore perhaps not surprising that alternative pA site usage is prevalent in the mammalian transcriptomes with ∼70% of all human genes subjected to what is commonly known as alternative cleavage and polyadenylation (APA).^134^

**Figure 3.**
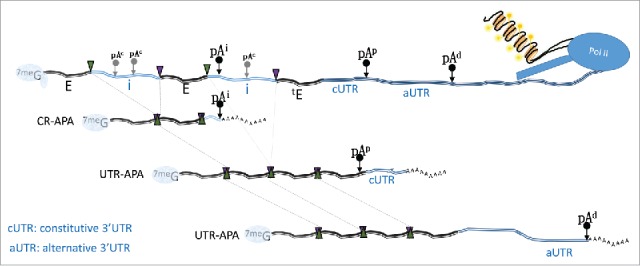
Coding region APA (CR-APA) and UTR APA. Depending on the location of the different pA sites, APA events can be classed into 2 major groups. CR-APA is the result of differential usage of pA sites that are located within the body of the gene and alternative usage produces APA mRNA isoforms that differ in their coding potential. UTR-APA summarizes events where the different pA sites are located downstream of the stop codon and alternative usage modulates 3′UTR length but does not change the coding potential. pA sites can be found in the intron and in the UTR of a gene. Intronic pA sites (pA^i^) are often cryptic poly A sites (pA^c^) that need to be actively repressed to enable gene expression. pA sites in the 3′UTR are generally separated into proximal (pA^p^) or distal (pA^d^) sites. Usage of the proximal sites generates mRNA isoforms that have a so called constitutive 3′UTR (cUTR) and isoforms that are generated by usage of the distal site contain both the constitutive and alternative 3′UTR (aUTR) regions. The respective resulting APA mRNA isoforms are indicated, dotted lines refer to the removal on introns (i) and fusion of exons (E) and the 5′ splice sites and 3′ slice sites are indicated by the green and purple triangles respectively. The terminal exon is indicated by “^t^E” and “^7me^G” refers to the 5′ cap.

APA occurs when a single gene has multiple pA sites, compared with constitutive polyadenylation, where one gene has one sole pA site. Like alternative splicing, APA results in multiple distinct RNA transcripts being produced from a single gene. The relative positions of these pA sites in a gene and their usage will ultimately determine the coding and regulatory sequence elements that are present in the different transcript isoforms. Depending on where the different pA sites are located in genes, APA events are separated into 2 major categories: untranslated region APA and coding region APA (Fig. 3).

**Untranslated region APA** (UTR-APA) occurs when multiple pA sites are available downstream of the terminal exon of a particular gene. As they are positioned outside the coding regions, differential usage of such pA sites does not alter the coding capacity of the resulting transcript isoforms but instead, alters the lengths of their 3′UTRs. The sequence that is absent in the short isoforms is commonly referred to as the alternative UTR (aUTR), relative to the common UTR (cUTR), which is present in both long and short APA isoforms. The presence or absence of an aUTR may equip a particular mRNA isoform with regulatory elements that influence the fate of the transcript regarding its stability, availability to the translational machinery and subsequent protein output, subcellular localization and even final protein destination. While aUTRs provide scope for potential regulation, it is not always clear what proportion of UTR-APA events have a physiologic impact.

**Coding region APA (CR-APA)** occurs when multiple pA sites are present at positions upstream of the 3′UTR. Thus CR-APA events can produce APA mRNA isoforms that differ in their coding capacity of the final protein product. Cryptic pA sites are also abundant in introns but, as mentioned above, they are actively suppressed by the U1snRNP.

In comparison to UTR-APA, it is easier to delineate a physiologic consequence for CR-APA events as they directly impact on the cellular proteome. Despite this, in the literature global analysis of APA is heavily bias toward UTR-APA.

## Transcriptome wide APA profiles

The sum of all UTR-APA and CR-APA isoforms constitute the cellular APA profile. Advances in next generation sequencing have allowed cellular APA profiles to be analyzed on a transcriptome wide scale and several different pipelines have been developed and are available to investigate APA events (Table 1). UTR-APA in particular has attracted significant attention in the last decade and a large number of APA profiles have since been determined and are accessible in various repositories including: [http://www.polyasite.unibas.ch]^3^ [http://genome.bucm.edu.cn/utr/].^135^ The analysis of these large data sets revealed that APA is highly dynamic and that the profiles can change depending on the context particular cells are studied in.

**Table 1.** Methods of 3′end targeted deep sequencing.

Category	Technique	Overview	References
3′end capture	3P-Seq	A biotinylated primer is added to the poly(A) tail and solely the poly(A) fragments can then be isolated using streptavidin. RT with just TTP is used to fill in poly(A) tail and RNase H is then used to cleave the poly(A) tail leaving just the very 3′end which is then used as input for sequencing library preparation.	^136^
		3′READS	Uses a unique chimeric CU_5_T_45_ oligo isolation system, which completely eradicates internal priming and amplification of oligo adenylated transcripts.	^137^
Direct RNA sequencing	DRS	DRS uses the Helicos BioSciences system which starts by using an oligo(dT) coated surface to which the poly(A) tail binds to. Reverse transcription using only dTTP is then used to fill in the entire poly(A) tail. Sequencing is then initiated from the most 3′prime non-A base.	^28,73,138^	
Oligo(dT)-based priming	3Seq	Standard oligo(dT)-based priming technique using an oligo(dT)_25_ containing RT primer, and sequencing the terminal 25 bp upstream of the cleavage site to map the pA site.	^139,140^	
		3′end RNA-Seq	Standard oligo(dT)-based priming technique using paired-end sequencing to obtain strand-specificity	^141,142^
		3′end-seq	Includes an *in vitro* transcription step after reverse transcription to increase the quality of the input RNA and as an amplification step.	^143^
		3Seq	Adapted slightly from 3Seq	^96,144,145^
		3′T-fill	Directly before sequencing, the poly(A) tail is filled using TTP resulting in sequencing starting from the base directly upstream of the cleavage site.	^146^
		A-seq	Uses an RT primer consists of an anchor nucleotide, followed oligo(dT) sequence with a stem-loop containing the adaptor sequence for priming the subsequent PCR reaction, in the middle of the oligo(dT).	^147^
		MAPS	Standard oligo(dT)-based priming using a random primer for second-strand cDNA synthesis.	^148^
		PAS-Seq	Variation to the standard oligo(dT)-based priming technique involves using the terminal transferase activity of MMLV reverse transcriptase, which allows generation of cDNAs with linkers in a single RT step, thus skipping several enzymatic steps.	^149^
		PolyA-seq	Standard oligo(dT)-based priming using a random primer for second-strand cDNA synthesis.	^134^
		Quant-seq	Commercialised standard oligo(dT)-based priming using a random primer for second-strand cDNA synthesis.	^150^
		SAPAS	Oligo(dT)-based priming using template switching and optimised primer anchoring in the RT-reaction to avoid sequencing in the long poly(A) tail.	^135,151,152^
		SMPSS	Uses a single-molecule system based on the HeliScope single molecule sequencer. It is amplification and ligation-free, allowing very little bias in quantitation.	^97^

## APA profiles in the context of proliferating and activated cells

A general pattern of shifts toward the proximal pA sites (UTR shortening) has been seen upon increased cellular proliferation.^145,153^ In the case of murine T-lymphocyte activation, the UTR-APA events show a clear pattern of a switch to proximal pA sites, compared with CR-APA, where there appeared to be equal movements toward both proximal and distal pA sites upon cell activation.^153^ Comparison with an extensive range of both murine and human cell lines revealed a similar pattern whereby the proliferation rate was inversely correlated with the 3′UTR length.^153^

The activation of neurons by depolarising agents also induces shifts from the distal to proximal pA sites in a subset of genes that are regulated by the MEF2 family of transcription factors.^154^ Unlike the examples described above, many of these APA shifts are CR-APA events and thus result in the expression of functionally distinct proteins that may be critical for synapse development.^154^ The generality of APA profile shifts in response to cell activation is further shown by shifts that are apparent when astrocytes are exposed to eukaryotic growth factor EGF, T-cells are activated by interleukin-2 and B cells are cultured in the presence of anti-IgM and CpG. Interestingly there appears to be little overlap in genes that are undergoing stimulus dependent APA. This indicates that these shifts are stimulus and also cell type specific,^154^ but how they are achieved is unclear in most cases.

Similar to activation, stressed cells tend to enhance the use of intergenic pA sites and produce 3′extended transcripts.^155,156^ While no clear trend toward shortening or lengthening in UTR-APA has been observed, intronic pA sites are suppressed upon exposure to stress.^157^

## APA profiles in the context of development, tissue and cell types and different species

APA profiles have also been found to vary as a function of the differentiation status of cells. This was first described during mouse embryonic development, where the differentiation of C2C12 myoblast cells to myotubes showed a general shift to distal pA sites (UTR lengthening).^158^ Conversely, if differentiation is reversed, and induced pluripotent stem cells are generated from fully differentiated somatic cells, a general 3′UTR shortening is observed.^159^ Widespread CR-APA and UTR-APA have been reported to be critical during spermatogenesis. Genes associated with sperm maturation and testis specific genes compared with ubiquitously expressed genes tend to undergo 3′UTR shortening early in spermatogenesis avoiding destabilising and transposable elements that are located in the aUTRs of these genes.^160^

When specific cell types or tissues are compared, they appear to have characteristic APA profiles.^16^ The most striking differences in APA profiles are those between mammalian and invertebrate testes and brain. While in testis the proximal pA sites are generally favored, in the brain the opposite is true and a preferred shift toward distal pA sites is characteristic.^161-164^ The prevalence of long 3′UTRs in neuronal tissue was reported using northern blot analysis over 20 y ago.^165^

In addition to simple switches between common pA sites, there are several examples of unique pA sites that are only activated in specific tissues such as testis^166^ and the brain.^161^ In testis, these sites appear to have a lower incidence of the AAUAAA canonical hexamer, as well as having unique upstream and downstream elements.^166^ Since these sites are not used efficiently in somatic cells, it has been suggested that testis-specific pA factors may be responsible for this pA site switching,^167^ such as the CstF-64 variant, τCstF-64.^168^

Similarly, tissue specific pA sites are also used in the brain where they generate specific APA mRNA isoforms with unusually long 3′UTRs ( >10 kb).^161^ These tissue specific APA events potentially provide a huge repertoire of diverse regulatory elements that can be plugged into tissue specific post-transcriptional regulatory networks.

The conservation of APA sites between species is limited and is restricted to fewer than 500 genes when tandem APA sites are compared between mouse and humans.^169^ If human pA sites are compared with rhesus, mouse, rat and dog, fewer than 1% are found in similar regions between them,^134^ indicating that APA is chiefly tissue specific, rather than species specific.

Experiments in yeast have compared the conservation of APA between different yeast species including *Saccharomyces cerevisiae, Kluyveromyces lactis*, and *Debaryomyces hansenii*.^170^ Here, despite the similarities in the general sequence preferences surrounding the pA sites, the APA profiles show species specificity. This led to the conclusion that much of the heterogeneity seen in the pA sites is likely to be biologic noise as defined by “biochemical events that occur *in vivo* as a result of a low-specificity process that has not been subjected to evolutionary optimisation.”^170^

Recently, single cell studies have started to expose the heterogeneity of pA site usage within a population of homogenous stem cells.^171^ This revealed that individual cells differ significantly in their choice of pA site usage. Interestingly, it appears that the heterogeneity in pA site choice observed in these cells surpasses that of what would be expected if pA site choice was random, thereby concluding that this variability has the potential to contribute to the functional cell-to-cell heterogeneity.^171^

## APA profiles in the context of cancer

Several studies have observed shifts to usage of proximal pA sites upon cellular transformation and carcinogenesis.^44,138,143,151,153,172-174^ It is thought that shifts to these proximal pA sites in cancer function to exclude instability elements present in the aUTR, avoiding particular regulatory networks, such as those involving miRNA-mediated destabilisation of specific transcripts. Accordingly, in many cases shortening can result in increased mRNA and protein levels of oncogenes, for example in *CCND1*.^172^ However, it is noteworthy that in the cancer cells that show general 3′UTR shortening, there are also a significant number of genes that undergo 3′UTR lengthening.^138,151,173^ This clearly highlights that APA patterns in transformed cells are complex making the interpretation of the profiles in the context of the underlying mechanisms and the physiologic impact of the shifts more difficult.

How cancer specific APA profiles are established is largely unclear, but the dysregulation of CFIm in some cancer cells can be linked to 3′UTR shortening and provides a mechanistic explanation for this cancer-specific APA feature.^44^

In addition to further the understanding of how cancer specific gene expression profiles are established, logging changes in APA profiles from tumor cells may also provide a valuable resource that can be exploited to develop tools to type cancer subtypes,^173^ provide information for diagnosis,^175,176^ and prognosis,^176-178^ and perhaps even treatment stratification. Indeed, APA profiles have previously been proposed to serve as biomarkers for cancer progression.^143^

## APA profiles in the context of other disease phenotypes

Many individual APA events have been linked with specific disease phenotypes such as thalassemia and IPEX syndrome.^11,179-181^ In these examples, pathogenesis is generally the result of mutations of *cis*-elements in one specific gene that compromises its 3′end processing efficacy. These mutations can result either in loss of function, such as in thalassemia and IPEX syndrome, or gain of function, as exemplified in thrombophilia and mantle cell lymphoma. In the latter, a point mutation in the *CCND1* gene causes activation of a novel pA site leading to 3′UTR shortening and transcript and protein overexpression.^182^

If proteins that regulate cleavage and polyadenylation are mutated, 3′end processing at many different pA sites can be altered and this can lead to changes in APA profiles. For example, the APA profiles between normal and failing hearts differ. While no clear global shifts in 3′UTR lengths are obvious, smaller cohorts of genes in the diseased compared with the normal heart show distinct changes in their APA profiles. Mechanistically the shifts in these APA profiles may be caused by the reduced expression levels of several cleavage and polyadenylation factors, including PABPN1. Interestingly, the levels of functional PABPN1 have before been linked to global shifts from distal to proximal pA sites in cells from oculopharyngeal muscular dystrophy (OPMD) patients.^96,97^ In OPMD cells the availability of functional PABPN1 is compromised by a triplet repeat expansion mutation of an alanine repeat in the PABPN1 gene.^183^ It has been suggested that in healthy cells, high levels of PABPN1 are available and can bind and suppress the proximal pA site in a significant number of genes. Conversely, in OPMD cells, the depletion of functional protein results in the activation of proximal pA sites causing 3′UTR shortening which may contribute to the pathology.^96^

Myotonic dystrophy, a neuromuscular disease, represents an additional example where disease specific APA profiles are dependent on the availability of functional proteins. The Muscleblind-like (MBNL) protein family are key regulators of alternative splicing and mutations that compromise their function are associated with myotonic dystrophy. Mutations that cause loss of function in MBNL proteins also result in large scale bidirectional APA shifts.^184^

Extensive APA defects that occur in the cerebellum of amyotrophic lateral sclerosis (ALS) patients have been reported. ALS patients that feature a repeat expansion in the C9orf72, have a higher frequency of short 3′UTR APA isoforms. The cause and the physiologic impact of these APA changes however is unknown.^185^

For all these examples it is important to keep in mind that it is not clear whether the shifts in APA profiles are driving disease development or progression, or whether they are a reflection of the disease dependent changes in the cells.

## Issues surrounding the global analysis and interpretation of APA profiles

As outlined above, APA profiles of cells appear to have characteristic signatures that are dynamic and can change in response to specific cues and disease. To elucidate the physiologic role of global APA changes, it is imperative to unravel the mechanisms and networks that are responsible for shaping these profiles. To do this, a distinction has to be drawn between alterations of APA profiles that are due to specific selection of one pA site over another at the point of cleavage, and changes in APA profiles that are due to post-transcriptional mechanisms, such as degradation of specific isoforms.

**Figure 4.**
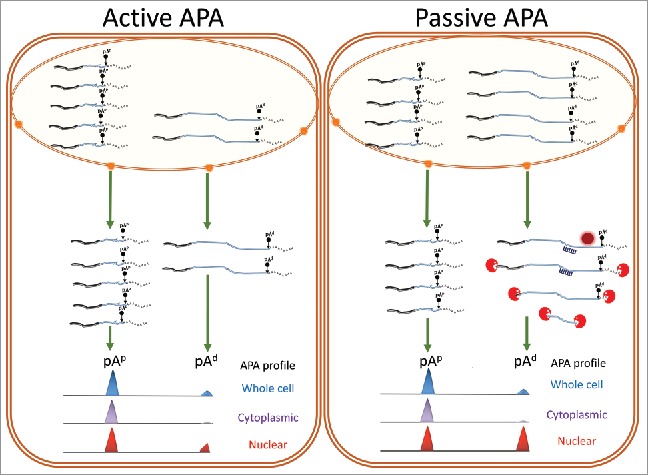
Distinguishing between ACTIVE and PASSIVE APA. The APA profile can either be modified at the point of cleavage (Active), or at the post transcriptional level (Passive). In active APA, factors that inhibit or enhance one pA site over another produce APA isoforms that can avoid a particular regulatory pathway. On the other hand, in passive APA, the availability of factors such as RBPs (dark red circle) and miRNAs (navy) in the cytoplasm alter the APA profile by specifically downregulating a particular isoform. For example, as depicted here, miRNAs can target the aUTR which can recruit the RNA induced silencing complex (RISC) result in degradation by exoribonucleases (red “PacMan”). Different RBPs that bind to the aUTR can either stabilize or degrade the isoform. In this case although the whole cell APA profile is the same, the nuclear APA profile is different, highlighting the importance of assessing changes in the cytoplasm compared with the nucleus to distinguish Active and Passive APA. This gives a better resolution of the causes that enforce specific APA changes in different environments.

APA profile changes that occur as a result of cleavage site selection can be regarded as **“active” APA** (Fig. 4, left panel). Active APA can be achieved by changing the availability of factors that either enhance or suppress the usage of a particular pA site over another in response to a specific cue or change in the state of the cell. Conversely, profile changes that are the result of post-transcriptional events can be described as **“passive” APA** and may occur in response to a specific cue that alters the availability of factors, for example miRNA levels, that act post-transcriptionally (Fig. 4, right panel). This includes processes that alter the APA profile by interfering with the nuclear export rates or the stability of specific APA mRNA isoforms.

As most APA analysis is performed using whole-cell RNA, active and passive APA events are difficult to distinguish (Fig. 4). This may complicate the evaluation of particular profiles and may lead to misinterpretations of the underlying mechanisms and thus the physiologic relevance of APA. For example, shortening events in the context of cancer are often attributed to the selection of proximal sites at the point of cleavage to avoid post-transcriptional control executed by miRNAs. This interpretation however overlooks the fact that miRNA levels are known to be subjected to dramatic changes during oncogenic transformation^186^ and that different cancer subtypes have distinctive miRNA signatures.^187^ Thus a significant number of the shortening or lengthening events may be due to passive APA acting at the post-transcriptional level. Liaw *et. al.* highlight this possibility by cross-referencing miRNA profiles with previous APA data showing apparent 3′UTR shortening in the breast cancer cell line, MCF-7.^151,188^ This analysis showed that genes with target sites for upregulated miRNAs in their aUTR exhibited a larger degree of 3′UTR shortening relative to those without targets, and the extent of this was influenced by the number of target sites. In addition, the positioning of target sites for miRNAs upregulated in MCF-7 cells showed that they were enriched in the aUTRs of genes where 3′UTR shortening was seen, relative to downregulated miRNAs. Combining this data, it was concluded that selective degradation of solely the longer transcript isoforms by upregulated miRNAs has a significant impact that contributed to the observed changes in APA profiles in MCF-7 cells.^188^

Perhaps many of these passive APA events in cancer cells are “collateral damage” caused by the upregulation or downregulation of particular miRNAs that aim to modulate a handful of specific genes. However, due to the nature of miRNA-mediated regulation, this will also affect many other genes that have little, if any, consequence for the progression or establishment of tumorigenesis.

The most common method used to untangle active and passive APA events is to look at the expression levels of all transcript isoforms.^144,153,177^ This approach argues that if observed shifts in APA patterns are due to a change in pA site choice, the increased proportion of the shorter transcripts leads to a lower overall decay rate, as 3′UTRs overall are of a repressive nature.

The expression level observed would, therefore, increase in this situation. Conversely, if the shift in APA pattern is due to an increase in the decay rate of solely the longer transcript, a decrease in expression would be seen. This method has its limitations, as any number of mechanisms, aside from APA, can influence transcript levels of a gene. To address this issue more directly, APA profiling of different subcellular fractionations have been used.^16,189^ In particular, the comparison between nuclear and cytoplasmic APA events can be used to get a better resolution on specific APA events and determine whether they are due to a change in cleavage site choice, or due to regulation at the post-transcriptional level including nuclear retention and transcript stability^16^ (Fig. 4). If subcellular APA is performed between different states of cells it may reveal how APA events are integrated with other regulatory networks that dynamically respond to changes in the state of cells.

## Factors that regulate active APA by influencing pA sites choice at the point of cleavage

**Figure 5.**
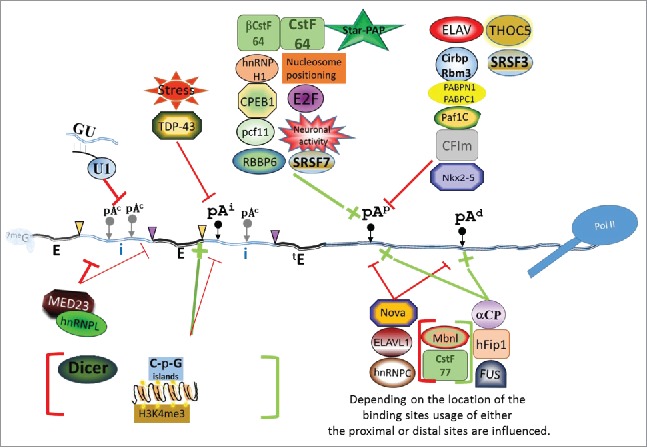
Factors that regulate APA at the point of cleavage. Numerous RNA-binding proteins, and environmental stresses have been associated with modulating active APA at the point of cleavage. Factors are grouped into enhancing (green) or repressing (red) effects on a particular site and factors that are between a green and red bracket can either enhance or repress a site depending on the circumstances. For more details, see Tables 2, 3 and 4 or download the interactive slide. Red lines indicate inhibitory effects on pA sites and green lines indicate enhancing effects of factors on particular pA sites. Black and gray dots with arrows indicate the position of the different types of pA sites: (pA^i^) = intronic pA site; (pA^c^) = cryptic pA site; pA^p^, proximal pA site; pA^d^ = distal pA site. The gene structure is detailed by specifying introns as blue double lines (i) and exons as black double lines (E) and the 5′ splice sites and 3′ splice sites are indicated by yellow and purple triangles respectively. The terminal intron is symbolised by ^t^E.

Despite the large number of reported shifts between pA sites in different cells and cell states, the precise mechanism of defining pA site choice in active APA remains ill defined. This is partly due to the complexity by which the strength of a pA site is defined and the many ways this can be modulated. In addition, as mentioned above, the difficulty to discriminate between active and passive APA further complicates the identification of factors. As the *cis*-elements influencing polyadenylation efficiency do not change, the switching between pA sites can only be achieved through the fluctuation of *trans*-factors or the presentation of pA sites to the processing machinery during transcription. The former is exemplified by the transplant of large chromosomal segments from *Debaryomyces hansenii* into *Saccharomyces cerevisiae* and the concomitant adoption of the host APA profile by the recipient.^170^ Several *trans*-factors have been shown to influence pA site choice (Fig. 5), including core pA factors (Table 2) and several RNA-binding proteins of diverse function (Table 3).

**Table 2.** pA-factors known to influence pA efficiency and may be involved in regulating APA.

Factor	Motif bound	Proposed model	References
CF_Im_	(UGUA)_n_	At high levels, CF_Im_ interacts with suboptimal CF_Im_ binding sites preventing the interaction of CPSF with these proximal pA sites and promotes usage of distal pA sites. Depletion of CF_Im_ allows the interaction of CPSF with proximal pA sites, resulting in 3′UTR shortening. At the single gene level increase of CF_Im_ causes distal pA usage in MeCP2, a protein which is important for brain function. Thus CF_Im_ mediated APA in MeCP2 links APA to neuropsychiatric conditions.	^44,83,147,190,191^
CstF64	U-rich	Co-depletion of CstF64 and CstF64τ leads to APA shifts in a small number of genes primarily to the distal pA site, which is thought to be reflective of the general higher efficiency of distal pA sites. Furthermore, CstF64 has been found to promote usage of weaker pA sites containing the downstream GUKKU motif.	^20,73,192,193^
CstF77		High levels of CstF77 result in activation of the pA site in intron 3 of CstF77 gene resulting in a negative feedback loop. Additionally, it influences both shortening and lengthening event changes in APA profiles of cell cycle genes, specifically where U-rich regions surround the pA sites.	^194^
hFip1		A component of CPSF complex. Regulation of APA by Fip1 is dependent on the distance between pA sites. When far apart, low-levels of Fip1 result in reduced pA efficiency and decreased use of weaker, proximal pA sites. When close together, Fip1 blocks CstF binding at the proximal site and therefore results in distal pA site usage.	^30,31^
Pcf11		Pcf11 is a component of CFIIm. It binds directly to the pre-mRNA and enhances the use of proximal pA sites through direct binding to the pre-mRNA.	^31,87^
βCstF-64		A neuronal splice variant of CstF64 that associates with the CstF complex and stimulates pA thereby activating weaker pA sites.	^195^
Star-PAP	AUA	Star-PAP is a noncanonical poly (A) polymerase. It associates with RNAs that have an AUA motif upstream of a pA site that also has a suboptimal DSE. This Star-PAP mediated selection of pA sites may play a role in the regulation of APA.	^21^
PABPC1		No obvious *cis*-element enriched around pA sites regulated by PABPC1, but, like PABPN1, promotes distal pA site usage. Shuttles between the cytoplasm and nucleus and may regulate cytoplasmic polyadenylation.	^31^
PABPN1		Promotes the use of distal pA sites by inhibiting pA at weaker proximal pA site through competition with CPSF for binding to the PAS. Reduced availability of functional PABPN1 in OPMD causes widespread 3′UTR shortening.	^96,97,196,197^

**Table 3.** Other RNA-binding factors known to influence pA efficiency.

Factor	Motif bound	Proposed model	References
α CP (αCP)	C-rich motifs	αCP binds mRNAs containing a subgroup of C-rich elements in their UTRs and acts as an upstream 3′end processing enhancer. Usage of distal or proximal pA sites can be influenced depending on upstream C-rich regions close to the respective pA site by varying αCP levels.	^198^
Cirbp and Rbm3	GNNGNNG	Upon cold-shock, these factors are upregulated and, through 3′UTR binding, inhibit the use of proximal pA sites.	^199^
CPEB1	CPE	CPEB1 shuttles to the nucleus binding cytoplasmic polyadenylation elements and enhances polyadenylation at nearby pA sites. Also, it prevents U2AF65 binding, which inhibits splicing. CPEB1 in the nucleus causes shortening and this correlates with cell proliferation.	^200^
DICER		Nuclear Dicer affects pA site usage by modifying the chromatin landscape surrounding the 3′end processing sites. In a region of closed chromatin Pol II progression is slowed down, increasing the likelihood that a weak pA site is recognized. In contrast if the weak pA site is in an open conformation, Pol II progression is fast decreasing pA site usage.	^16^
ELAV (*Drosophila)*		In the neuronal tissues, ELAV is recruited to the promoter-paused Pol II complex. Upon resuming transcription, ELAV is deposited near proximal pA sites, inhibiting their usage, resulting in extended 3′UTRs.	^201,202^
FUS	UGGUU	FUS binds directly downstream of a proximal pA site, which enhances CPSF160 recruitment and activates the pA site leading to short transcripts. If there is no pA site upstream of a FUS binding site, FUS binding causes Pol II stalling and premature termination, producing short transcripts that are not polyadenylated.	^203^
hnRNP C	U-rich	hnRNP C binds to U-rich sequences, which masks the pA site in its vicinity to represses their use. The transcripts affected by hnRNP C mediated APA are enriched in ELAVL1 binding sites and this process may thus be linked to the HuR (*ELAVL1*) mediated recruitment of specific mRNA isoforms to the Endoplasmic Reticulum.	^3^
hnRNP F	G-rich DSE	Competes with CstF-64 by binding to G-rich motifs near pA sites.	^204^
hnRNP H1	Auxiliary DSE	Depletion results in a general shift to distal pA sites, with hnRNP H1 binding sites surrounding proximal pA sites.	^205^
hnRNP H2	G-rich	Binds near pA sites and enhances binding of CstF-64.	^206^
hnRNP K	UCCCUU	Competes with CFI for binding the pre-mRNA, reducing pA efficiency and reduced usage of that pA site.	^207^
hnRNP L	CA-rich elements	Functions as a splicing regulator, so altering levels of hnRNP L can sway the balance between competing splicing and intronic pA events	^208^
HuR (*ELAVL1*)	AU-rich elements (AREs)	HuR (*ELAVL1*) binds to AREs in transcripts. Through competition for binding sites on pre-mRNAs, HuR can influence both APA and splicing, including its autoregulation.	^209,210,211^
		When associated with particular aUTRs, HuR can also control the final destination of the protein product. For example, the *CD47* aUTR mRNA isoform protein product is preferentially located to the plasma membrane, whereas the short UTR isoform lacking HuR sites translates *CD47* that remains predominantly in the ER.	
Mbnl proteins	R/YGCY	Muscleblind-like proteins (Mbnl) are important regulators of alternative splicing during development. Mbnl is also implicated in APA and can either inhibit pA site usage if it binds close to a pA site or enhance pA site usage if it binds further upstream. Inhibition is thought to occur through steric hindrance. Mbnl is critical for creating a normal APA landscape during development and dysregulation of this process is associated with myotonic dystrophy.	^184^
MED23		Mediator complex subunit 23 (MED23) interacts with hnRNP L and affects hnRNP L regulated APA events, possibly by controlling hnRNP L occupancy at the promoter.	^212^
Nkx2-5		In conjunction with Xrn2, Nkx2-5 regulates pA site usage which is of high importance during mouse heart development. This tissue specifically expressed factor regulates APA, and its knockdown causes 3′UTR lengthening.	^213^
Nova	YCAY	NOVA is a neural-specific factor that binds YCAY elements in the 3′UTR. Depending on the location of these motifs, binding of NOVA can influence pA site choice by suppressing their use.	^214^
Paf1C		Depletion of some Paf1C subunits (Paf1, Cdc73,Ski8) results in global 3′UTR shortening. Regarding CR-APA, only Paf1 and Cdc73 depletion activated coding region pA sites. Paf1C subunits also play a role in suppressing transcription site intronic pA sites. Absence of Paf1 may cause increased Pol II pausing, which stimulates recognition of a pA site in the coding region.	^215^
PTB	G-rich USE	PTB competes with CstF64 to bind the DSE, thereby inhibiting pA site usage. However, it can also aid recruitment of hnRNP H1, which stimulates pA site usage.	^216^
RBBP6	unknown	RBBP6 competes with its isoform iso3 for binding with the core pA machinery. When RBBP6 is bound, it enhances pA site cleavage efficiency and promotes the use of weaker proximal pA sites. RBBP6 and iso3 particularly affect APA in transcripts that have AU-rich 3′UTRs such as c-jun.	^217^
SRm160	Unknown	Enhances pA through the association with CPSF.	^218^
SRSF3	CNUC	Promotes biogenesis of long 3′UTR APA isoforms and regulates their nuclear cytoplasmic export.	^219^
SRSF7		Promotes biogenesis of short 3′UTR APA isoforms and regulates their nuclear cytoplasmic export.	^219^
TDP-43	UG rich	High levels of TDP-43 cause inhibition of pA1 site in intron 7 of its own *TARDBP* pre-mRNA, resulting in usage of pA2 or pA4, which both produce transcripts that are targeted by the Nonsense Mediated Decay pathway, providing a mechanism of auto-regulation.	^220^
THOC5		THOC5 is a member of the human transcription export complex (TREX). THOC5 knockdown activates proximal pA site usage. It is suggested that THOC5 recruits CF_Im_68 to target genes, promoting distal pA site usage.	^65^
U1 snRNP	AGGURAGU	Suppresses cryptic pA sites in the gene body, which is essential for the formation of full-length transcripts. Shown to suppress premature transcription termination in polycistronic pre-mRNAs in *C. elegans* by inhibition of pA site recognition by Pol II associated poly(A) factors.	^121,122,124,221,222^
				U1 snRNA levels drop after UV-induced DNA damage and activate intronic pA sites.
U1A	AUGCN(1–3)C	Component of U1 snRNP which binds GU-rich regions downstream of pA sites inhibiting the binding of CstF64, thus inhibiting polyadenylation. U1A can also bind to PAP inhibiting the polyadenylation reaction itself.	^223,224^	
				U1A is known to inhibit polyadenylation of its own mRNA, and has also been shown to act independently of U1 snRNP to inhibit polyadenylation of the *SMN* transcript.
U2	3SS	U2 interacts with CPSF and enhances polyadenylation efficiency.	^114^	
U2AF	Pyrimidine tract	U2AF interacts with CFI stimulating pA.	^111^	

As can be seen from Tables 2, 3 and 4, there is a plethora of *trans*-factors that, when depleted or compromised, cause shifts in APA profiles.

**Table 4.** Features and conditions that can influence pA site choice.

Factor	Motif bound or affected	Proposed model	References
DNA methylation (imprinting)	CpG islands	The methylation status of CpG islands influences pA site selection in the murine imprinted gene *H13.* This is thought to act via an unidentified DNA methylation sensitive pA factor. Similarly, in the Herc3/Nap1i5 locus, the regulation of an internal pA site and the downstream pA site that produces full length Herc3 mRNAs is controlled by DNA methylation at internal CpG islands.	^234,235^
E2F	TTGGCGG	Through enhanced proliferation, increased levels of the transcription factor E2F result in the increased use of proximal pA sites by upregulation of key 3′end processing genes.	^145^
Nucleosome positioning		High nucleosome occupancy directly upstream of proximal pA sites generally correlates with increased proximal pA usage.	^231,232^
Transcription rate		Slow transcription rates result in a longer time between when the proximal and distal pA sites are transcribed thereby causing in an increased probability of proximal pA site utilization. At the single gene level, pausing downstream of the intronic (μS) pA site in the *IGHM* gene can coordinate 3′end processing factor recruitment and pA site usage.	^16,230,234^
H3K4me3 levels		Chromatin status regulates pA site choice. An “open chromatin” state as measured by high H3K4me3 levels in spermatids compared with spermatocytes influences pA site usage resulting in global UTR shortening accompanied with greater transcript stability.	^160^
Neuronal activity		Neuronal activity promotes the use of proximal and internal pA sites affecting many transcription factor MEF2 target genes.	^154^
Stress: arsenite, anisomycin, viral stress		Viral stress or cells exposed to stress agents such as arsenite and ansiomycin tend to enhance the usage of intergenic pA sites and generate 3′extended transcripts. Ansiomycin mediated stress also suppressed intronic pA sites and pA sites that are located in the ORF. No clear trend is observed regarding 3′UTR-APA events.	^155,157^

A number of RNA binding proteins have been associated with influencing pA site usage, that primarily have other nuclear functions, as outlined in Table 3. Many of these mediate splicing, again highlighting the interconnection between these 2 pre-mRNA processing mechanisms.

As summarised in Table 4, several features that modulate gene transcription have also been linked to active APA. It has been long known that pre-mRNA processing and transcription are tightly coupled.^2^ Cleavage and polyadenylation are essential for transcription termination,^225^ as well as the recycling of transcriptional factors and the re-initiation at upstream promoters.^226^ The phosphorylated CTD of Pol II also provides an essential platform for the recruitment of several pA factors to the pre-mRNA.^2^ Specifically, CPSF and CstF, together with Pcf11, are brought to the pre-mRNA through the interaction with Pol II.^227^ The pausing of Pol II during transcription, and the subsequent recruitment of CstF to the transcription elongation complex, are decisive in pA site choice.^228^ Even after the Pol II has passed the pA site, the RNA tether between the 2 is necessary for the assembly of the mature protein complex, to perform efficient cleavage and polyadenylation.^229^

It is therefore not surprising that APA is also governed by parameters that compromise the kinetics of transcription, including Pol II transcriptional rates, chromatin structure, and histone modifications.^16^ This is beautifully exemplified in mutant *Drosophila* strains that reduce transcription elongation rates of Pol II which results in the preferential use of proximal pA sites in several alternatively polyadenylated genes.^230^ This proposed mechanism is analogous to the one proposed for exon skipping, whereby a faster Pol II reduces the latency period between when the proximal and distal pA sites are transcribed, thus reducing the probability of using the generally weaker proximal pA site.^16^ Furthermore, the conformation of chromatin has been shown to influence APA.^160^ Studies looking at nucleosome positioning surrounding pA sites show a general depletion of nucleosomes immediately downstream of high-usage pA sites, but then an enrichment past ∼100 bp downstream of the pA site, when compared with low-usage pA sites.^231,232^ The influence of transcription on cleavage and polyadenylation is not limited to the 3′end of the gene. The presence or absence of specific transcriptional activators can stimulate transcription-coupled cleavage and polyadenylation in yeast through Paf1c, which is thought to facilitate the formation of the pA factors in the elongation complex.^233^ In mammalian cells, the Paf1 complex is also implicated in pA site regulation. Here, the depletion of some subunits (Paf1, Cdc73, Ski8) caused pervasive transcript shortening of UTRs, and reduction in Paf1c or Cdc73 also increased usage of pA site located in upstream introns/exons. It appears that Paf1c depletion affects Pol II progression through the gene affecting pA site selection. Absence of Paf1c function causes slowing of the polymerase which would favor the more proximal positioned sites.^215^ Furthermore, the promoters of specific genes in *Drosophila*, have been shown to be essential in the recruitment of ELAV to Pol II, which subsequently acts at the 3′ends of those genes to inhibit proximal pA site, resulting in 3′UTR extension.^201^ The upregulation of specific transcription factors in response to cell activation, for example E2F, can influence APA profiles indirectly by increasing the gene expression of key 3′end processing factors.^145^

## The impact of active APA on the regulation of gene expression

**Figure 6.**
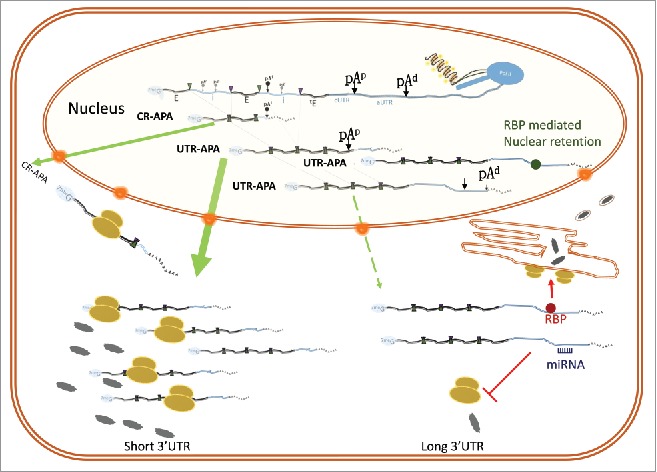
Consequences of APA: APA-isoform dependent decay rates and protein output. The 3′UTR length changes arising from APA can have implications on mRNA localization and transcript stability, which can impact on protein output and also determine the final destination of the encoded protein. This figure depicts the case where a short 3′UTR evades miRNA target sites in the aUTR, making it a more stable transcript, enabling increased protein output (protein symbolised by gray globules; ribosomes symbolised by mustard colored structures). The longer isoform shown here is bound by an RBP (dark green) in the nucleus, which prevents its export into the cytoplasm. The transcripts that are exported can be targeted for degradation by miRNA binding to the aUTR. The aUTR of the longer isoform can also bound by an RBP (dark red circle) in the cytoplasm which alters the localization of the transcript, for example in close proximity to the Endoplasmic Reticulum, for protein synthesis. Therefore, the UTR is important in mediating nuclear export, transcript stability, translatability and mRNA localization and the modulation of this is achieved by changing the expression of RBPs and miRNAs.

Modulating the length of the UTRs by APA has the potential to produce mRNA isoforms that can be subjected to distinct post-transcriptional regulation (Fig. 6). As 3′UTRs are thought to have an overall repressive role,^144^ cleavage and polyadenylation of a transcript at a pA site more proximal to the stop codon is generally considered to result in the removal of potentially repressive *cis*-elements in the 3′UTR, summarised in Table 5. This can, therefore, stabilize the transcript, or promote its translation, thus increasing the overall expression of that gene (Fig. 6). However, several RNA binding proteins have been identified to bind to the 3′UTR and stabilize the transcript, for example the Hu family of proteins (including HuR), which bind to AREs^236,237^. The neuronal specific HuD protein stabilizes transcripts required in neurite outgrowth enabling neuronal differentiation in several cell lines.^238,239^

**Table 5.** *Cis*-elements in the 3′UTR.

class of *cis*-elements	Sequence element	Overview	References
AU-rich elements (AREs)	AUUUA	These are present in 5–8% of all genes and can trigger mRNA destabilisation and translational repression. This is triggered by the binding of ARE-binding proteins (ARE-BPs), including TTP.	^240-242^
				The Hu family of proteins bind AREs and stabilize the corresponding transcript, particularly during neuronal differentiation.
GU-rich elements (GREs)	GUUUG	Contained in at least 5% of human mRNAs and triggers mRNA deadenylation and degradation. Acts through binding of proteins from the CELF family.	^243^	
CU-rich elements (CUREs)	(C/U)CCAN_x_CCC	PTB is the best-characterized CURE-binding protein and can affect translational repression, polyadenylation and mRNA stability.	^244^	
			(U/A)Py_x_UC(C/U)CC	
CA-rich elements (CAREs)	(CA)_n_	A stabilizing dinucleotide repeat, which acts primarily via hnRNP L binding, which alters the susceptibility of the mRNA to endo- and exonucleases.	^245^	
microRNA target sites	NNNNNNN	By far the most common destabilising element and target sites are present in > 60% of all genes. Regulation is primarily done via destabilisation of target mRNA (> 84%), rather than translational inhibition.	^246,247^

**Table 6.** Examples of genes producing differentially regulated UTR-APA isoforms.

Gene	Gene function	Summary	References
*CCND2*	Cell cycle regulator	3′UTR shortening is seen in cancer cell lines relative to normal tissues, thereby avoiding regulation by miR-15/16. Preferential use of the proximal pA site has been shown to increase the number of cells present in S-phase.	^172^
*CDC6*	Cell cycle regulator	Usage of the proximal pA sites avoids miRNA-mediated repression, resulting in increased CDC6 protein levels. This is triggered by the potent proliferation signal 17β-estradiol (E2), and may, therefore, be a mechanism by which the cell promotes cell cycle progression in response to proliferation signals.	^251^
*ELAVL1* (HuR)	RNA-binding protein	The aUTR region of HuR mRNA region contains an ARE region where HuR and TTP competitively bind, resulting in mRNA stabilization or destabilisation respectively. This, therefore, creates an autoregulatory loop, which may amplify the pathological role of HuR.	^254^
*MAPT*	Stabilises microtubules, specifically in neurons	UTR-APA isoforms are differentially regulated in neuroblastoma cell lines, with miR-34 family members targeting solely the distal APA isoforms. This gene encodes the Tau protein, which is one of the key components of protein aggregates formed during Alzheimer disease.	^255^
*MGMT*	DNA repair	Glioblastomas are shown to shift pA site usage to a distal site, resulting in the inclusion of target sites for miR-767-3p, miR-181d and miR-648, thus reducing the expression of *MGMT.*	^250^
*PAX3*	Transcription factor which controls myogenesis	In quiescent muscle stem cells, APA results in the production of *PAX3*, a key myogenic regulator, with a shortened 3′UTR allowing escape from regulation via miR-206 that targets the aUTR.	^249^
*PDCL*	Putative modulator of heterotrimeric G proteins	Several AREs are located between 2 pA sites in the 3′UTR of *PDCL*, which results in a significantly shorter mRNA half-life of the longer transcript.	^253^
*ZFR*	Neuron development	*ZFR* APA isoforms are differentially regulated by miR-579, which itself is co-transcribed with the *ZFR* gene. miR-579 also regulates *CPSF2*, creating a negative feedback loop wherein transcription of *ZFR* results in the production of miR-579, which targets CPSF2, favoring usage of the proximal pA site, which is resistant to regulation by miR-579.	^252^
				Transcription of ZFR to produce miR-579 also regulates CPSF2 in a negative feedback loop. The longer CPSF2 isoform is targeted by miR-579, favoring the usage of the proximal pA site, which is resistant to regulation by miR-579.

## The impact of APA on transcript isoform stability and translation

Global comparisons of *cis*-elements located in the aUTRs compared with cUTRs revealed a bias toward a higher representation of conserved miRNA seed regions in aUTRs.^153,158,248^ The miRNA target sites in aUTRs are also located in regions with higher AU content. This reduces the possibility that these target sites are involved in secondary structures and thus would represent better targets for miRNAs.^158^

Individual gene examples such as *PAX3, CCND1, CCND2, CDC6, MAPT* and *MGMT* support that the biased distribution of miRNA binding sites in APA mRNA isoforms can act as a potent layer to govern miRNA mediated regulation of gene expression.^172,249-252^ Similarly, APA in *ELAVL1* and *PDCL* regulates their expression by controlling the presence or absence of destabilising or stabilizing RNA binding protein recognition motifs (Table 6).^253,254^

However, despite these individual gene examples and the general biased distribution of *cis*-elements, the actual global impact of APA on isoform stability and transcript levels has been found to be fairly modest.^97,144,145,151^ This was further supported by more detailed analyses addressing the impact of UTR shortening on mRNA isoform half-life times in proliferating T-cells^265^ and mouse 3T3 cells, where aUTRs showed a limited influence on the stability of APA mRNA isoforms.^257^ In addition, contrary to expectations, a significant proportion of APA mRNA isoforms with extended UTRs have been shown to have increased stability in 3T3 cells,^257^ a phenomenon that was also observed in adipocyte stem cells.^258^ Following this trend, a recent analysis of nuclear and cytoplasmic fractions confirmed that the impact of UTR-APA on the stability is modest.^16,259^ In HEK293 cells, around 10 percent of all APA events are found to be subject to post-transcriptional regulation and around 3% of all those cytoplasmic events are destabilised by miRNAs. Interestingly, this study further showed that miRNAs target both short and long UTR-APA isoforms for degradation.^16^

The lack of a global impact of APA on mRNA stability in mammalian cells however is not universal. Contrary to mammals, in yeast, changes in UTR length have comprehensive implications on transcript stability and even changes at a single nucleotide level can have a significant large scale impact on transcript stability.^260,261^

The impact of different APA mRNA isoforms on translation efficiency follows a similar pattern. While the APA mediated shortening of UTRs in several mRNAs including *CDC6*,^251^ *HSPA2*,^262^ *ECE-1*,^263^ *CCND2* and *DICER1*^174^ was concomitant with a higher translational output, the opposite was true for many genes in 3T3 cells.^257^ Most notably in the *SERT* gene (serotonin receptor), the distal isoform is translated at a higher rate.^264^ As for the impact on stability, APA controlled UTR shortening appears to have a limited global impact on translation efficiency.^256,257^

While there is not a clear global trend, it is nevertheless clear from these examples that the differential usage of pA sites can alter the stability of selected transcripts and affect their overall protein expression, consequently having a significant impact on major biochemical processes.

## UTR-APA and the control of subcellular localization of transcripts

UTRs can play an important role in directing the subcellular localization of transcripts^265^ and it is therefore not surprising that UTR-APA influences subcellular distribution of mRNA isoforms. 3′UTR lengthening in the brain is one of the most striking examples of tissue-specific APA^161^ and this also appears to provide an important platform to control the localization of APA mRNA isoforms. *BDNF*,^266^ *Ranbp1*,^267^ *Impa1*,^268^ *MKK7*^269^ *and KPNB1*^270^ are examples where APA isoforms with long 3′UTRs include localization signals resulting in targeted transport and translation of the respective transcripts. Similarly, 3′UTR extension in α-synuclein transcripts, which are associated with Parkinson disease pathology, not only increases the translational output but also affects their localization away from the synaptic-terminals to mitochondria.^271^ On a more global scale, a recent comparison between RNA isolated from the dendrites and the stroma in neurons found UTR-APA isoforms that display differential subcellular localization. Distal APA isoforms that are induced during neuronal differentiation are more likely to be localized in neurite projections than their proximal APA counterparts.^189^

APA mediated subcellular localization is not just restricted to highly polarized cells. At the individual gene level, it has been shown that the presence of *Alu* repeats in the aUTRs of the *Nicolin1* gene is incompatible with nuclear cytoplasmic export in HEK293 cells.^272^ In a more recent global study, a significant number of nuclear retained APA mRNA isoforms have been identified when nuclear and cytoplasmic APA profiles in HEK293 cells were compared. At least for some of these APA isoforms incomplete splicing resulting in intron retention is instrumental to prevent export into the cytoplasm.^16^ In addition, APA isoforms with short 3′UTRs tend to be overrepresented in the cytoplasm compared with the nucleus and while this phenomenon is conserved between different cell types, the genes affected are cell type specific.^16^ Interestingly, 2 prominent splicing factors, SRSF3 and SRSF7 have recently been linked to both pA site selection and selective nuclear cytoplasmic transport. SRSF3 promotes the usage of distal pA sites and by recruiting the export factor NXF1, controls the export of long 3′UTR APA isoforms. In contrast, SRSF7 causes a shift toward proximal pA site usage and subsequently supports the export of the resulting short UTR-APA isoforms.^219^

UTR-APA controlling localized translation has also been seen with some membrane bound proteins including *CD47.* The distal APA isoform of *CD47* allows binding of HuR and SET resulting in the translated protein to be localized to the plasma membrane. Conversely, the proximal APA isoform translated *CD47* locates primarily to the endoplasmic reticulum.^211^

## The impact of CR-APA

In contrast to UTR-APA, CR-APA by definition, will affect the protein identity, as introducing a pA site upstream of a translational stop codon will result in a protein with a different C-terminus. This may be a simple truncation in the case of exonic CR-APA, or more commonly a different C-terminus in composite-intronic CR-APA or skipped-intronic CR-APA (Fig. 1). In the case of skipped-intronic APA, polyadenylation is coupled to an alternative splicing event of the alternative terminal intron and is therefore in dynamic competition with splicing.^273^ Thousands of these intronic pA sites have been identified, but largely remain dormant through inhibition by factors such as U1 snRNP^73^ (see above). However, these intronic pA sites can indeed be activated, as is seen during increased proliferation.^145^ In around 7.5% of breast cancer patients, a short version of *MAG1I3* as a result of premature polyadenylation at a cryptic pA site located in intron 10 of the gene is present. The shortened MAHI3 protein product promotes mammary cell transformation. However, the activation of this cryptic pA site is unclear as it is not dependent on mutations of local cis-elements and thus is more likely to involve an imbalance of trans-factors.^156^

The classic example of physiologic relevant CR-APA is that of the switch between the membrane-bound form of IgM in B-cells to the secreted form in plasma cells.^274^ This switch is thought to be governed by changes in the CstF-64 levels, whereby an elevation of CstF-64 in plasma cells activates the weaker proximal pA site, triggering the switch to the upstream pA site, ultimately removing the membrane anchoring C-terminus.^193^

One important constraint of CR-APA isoforms is that they do not result in the introduction of premature translational termination codons. Therefore, they are not subject to nonsense-mediated decay and have the capability of producing alternative protein isoforms with potentially distinct physiologic roles.^275^ CR-APA events also include examples where the polyadenylation event within the coding region of an exon can convert a tyrosine codon (TAT) into a stop codon (TAA). This mechanism regulates expression of a truncated form of glutamyl-prolyl tRNA synthetase that enables the escape of inflammatory genes from the GAIT complex mediated translational silencing.^276^

## Outlook

The advent of high-throughput sequencing technologies has no doubt made a huge contribution to our understanding of 3′end formation and APA. It has allowed us to map pA sites on a transcriptome wide scale and compare their differential usage in tissues and diverse cellular states. While in the past the focus was on mapping “global” trends such as 3′UTR lengthening and shortening in different cellular contexts, in the future the focus has to shift more toward identifying APA events that have a proven physiologic impact. This requires a clear distinction to be made between active and passive APA events. Furthermore, rather than just bioinformatics correlations, the physiologic consequence of such APA events also has to be confirmed by direct experimentation at the individual gene level.

The appreciation of APA as a global regulator of gene expression in recent years has also revived a renewed focus on the mechanistic side of pA site recognition. Despite the simplicity of the reaction, it is still unclear how the pA machinery can assemble into functional complexes at pA signals that deviate considerably from the consensus sequences and differ widely in their architecture. Developing methods that can assess the likelihood of a pA site being subjected to regulation^277^ and further dissecting the mechanistic details of pA site recognition will also be critical to fully understand APA and its physiologic impact.

## Supplementary Material

Supplementary_Material.pptx
