# Supplementary material for: Cleavage and polyadenylation: Ending the message expands gene regulation
Source: RNA Biol. 2017 Apr 28;14(7):865–90. doi: 10.1080/15476286.2017.1306171 (PMC5546720; doi:10.1080/15476286.2017.1306171)
Supplement: Supplementary_Material.pptx [file krnb-14-07-1306171-s001.pptx]

## Slide 1
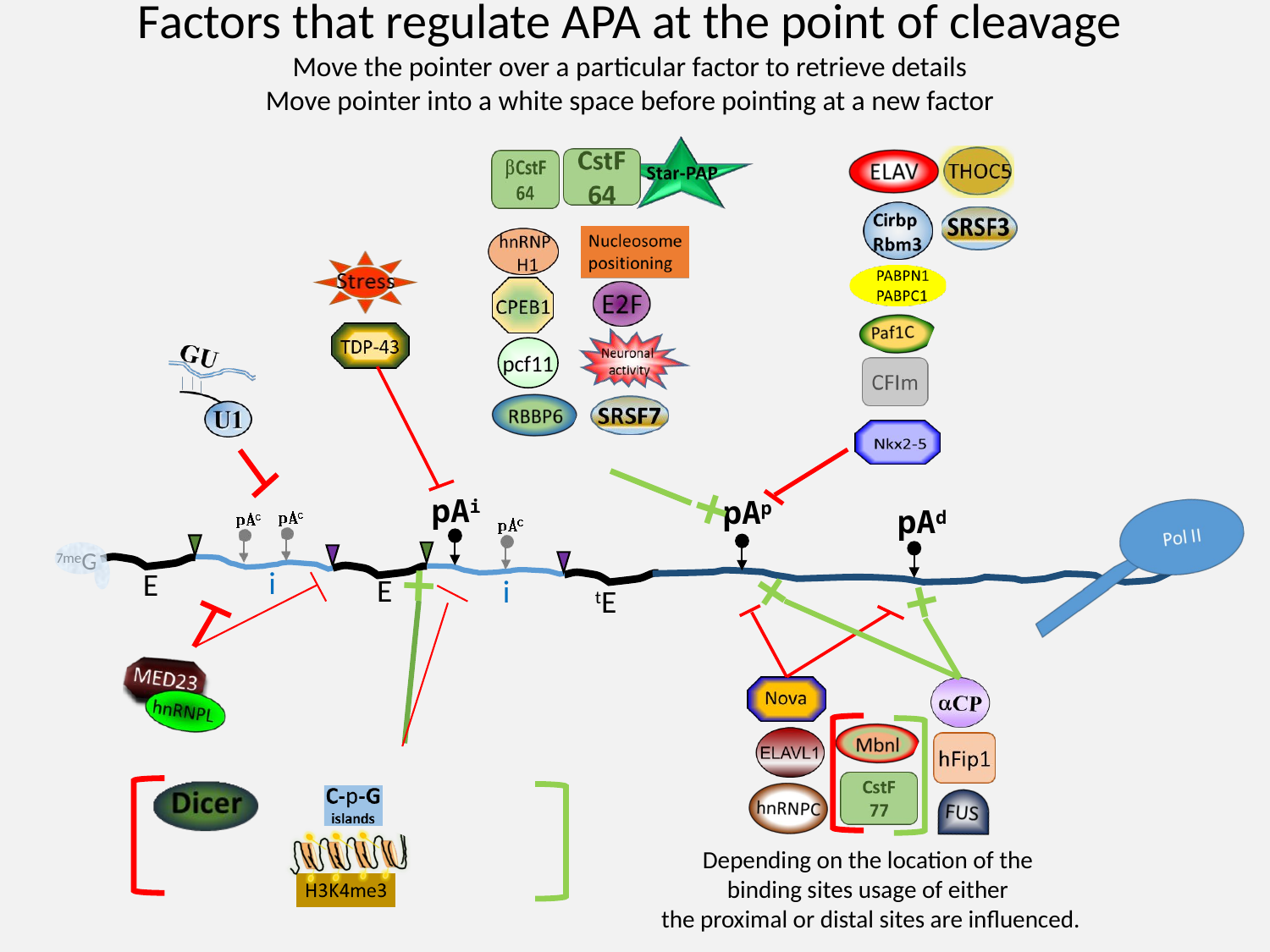

Factors that regulate APA at the point of cleavage
Move the pointer over a particular factor to retrieve details
Move pointer into a white space before pointing at a new factor
pcf11
+
pAi
pAp
pAd
+
7meG
+
+
i
E
E
i
tE
Depending on the location of the
binding sites usage of either
the proximal or distal sites are influenced.

## Slide 2
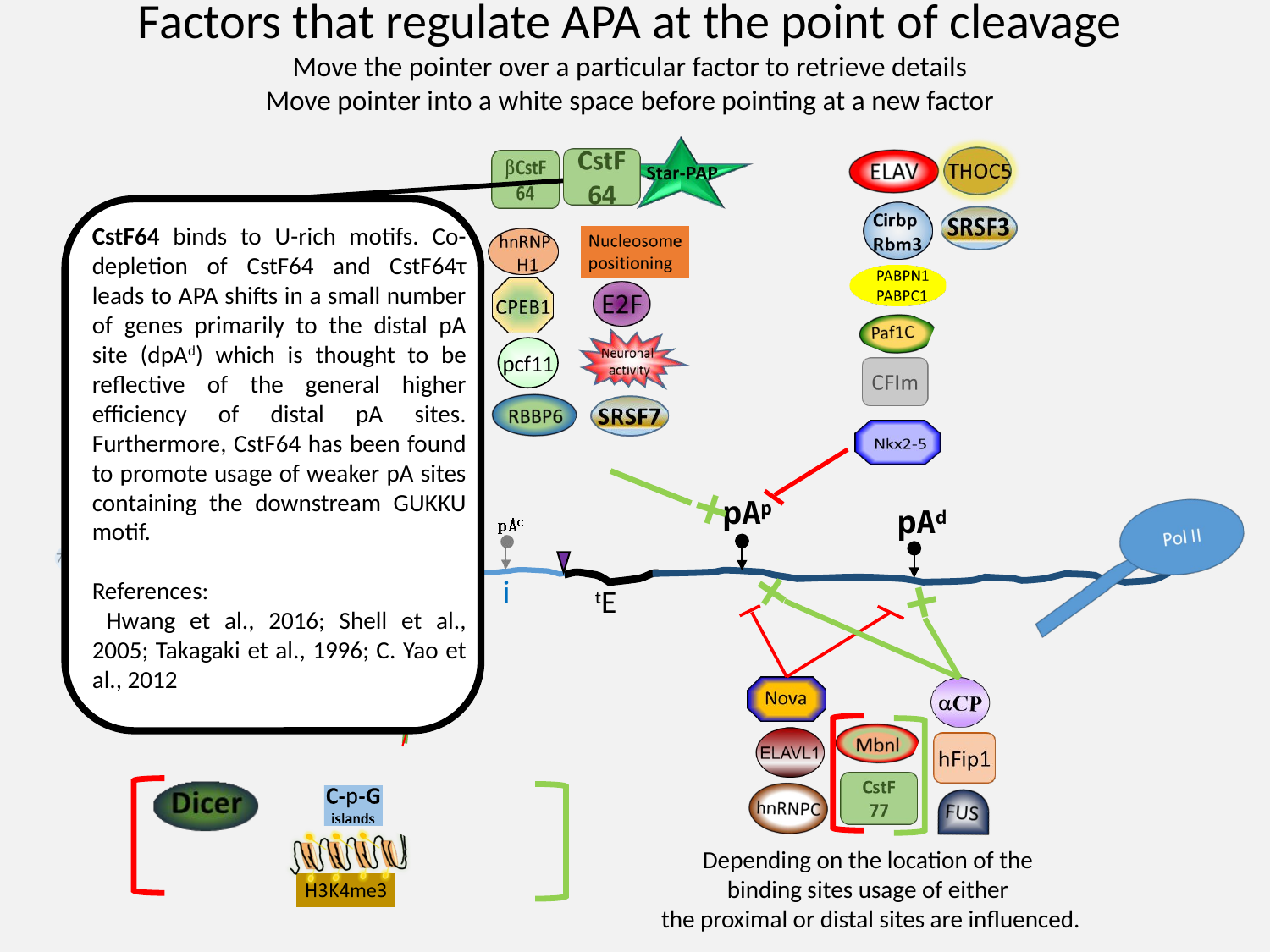

Factors that regulate APA at the point of cleavage
Move the pointer over a particular factor to retrieve details
Move pointer into a white space before pointing at a new factor
CstF64 binds to U-rich motifs. Co-depletion of CstF64 and CstF64τ leads to APA shifts in a small number of genes primarily to the distal pA site (dpAd) which is thought to be reflective of the general higher efficiency of distal pA sites. Furthermore, CstF64 has been found to promote usage of weaker pA sites containing the downstream GUKKU motif.
References:
 Hwang et al., 2016; Shell et al., 2005; Takagaki et al., 1996; C. Yao et al., 2012
pcf11
+
pAi
pAp
pAd
+
7meG
+
+
i
E
E
i
tE
Depending on the location of the
binding sites usage of either
the proximal or distal sites are influenced.

## Slide 3
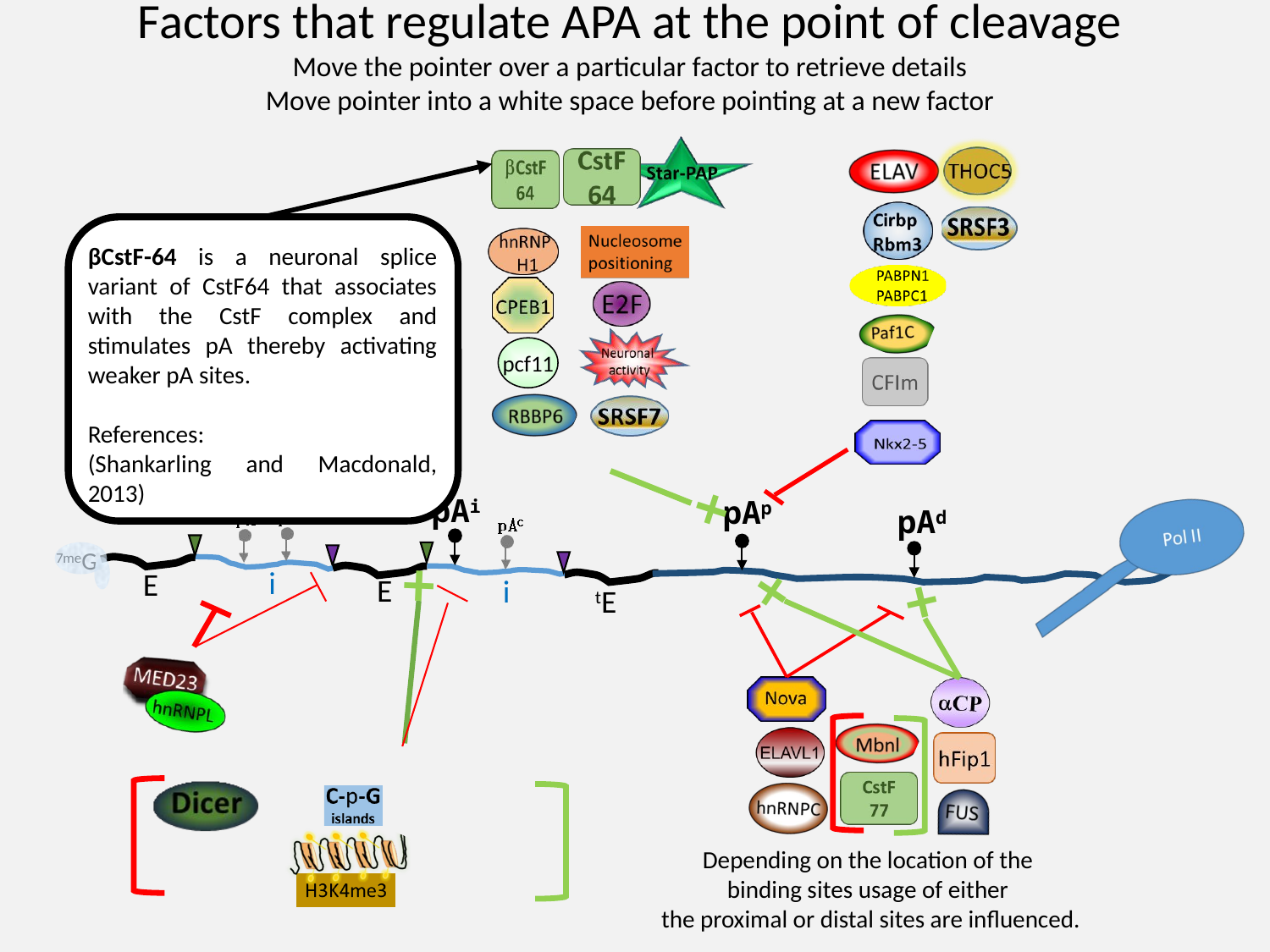

Factors that regulate APA at the point of cleavage
Move the pointer over a particular factor to retrieve details
Move pointer into a white space before pointing at a new factor
βCstF-64 is a neuronal splice variant of CstF64 that associates with the CstF complex and stimulates pA thereby activating weaker pA sites.
References:
(Shankarling and Macdonald, 2013)
pcf11
+
pAi
pAp
pAd
+
7meG
+
+
i
E
E
i
tE
Depending on the location of the
binding sites usage of either
the proximal or distal sites are influenced.

## Slide 4
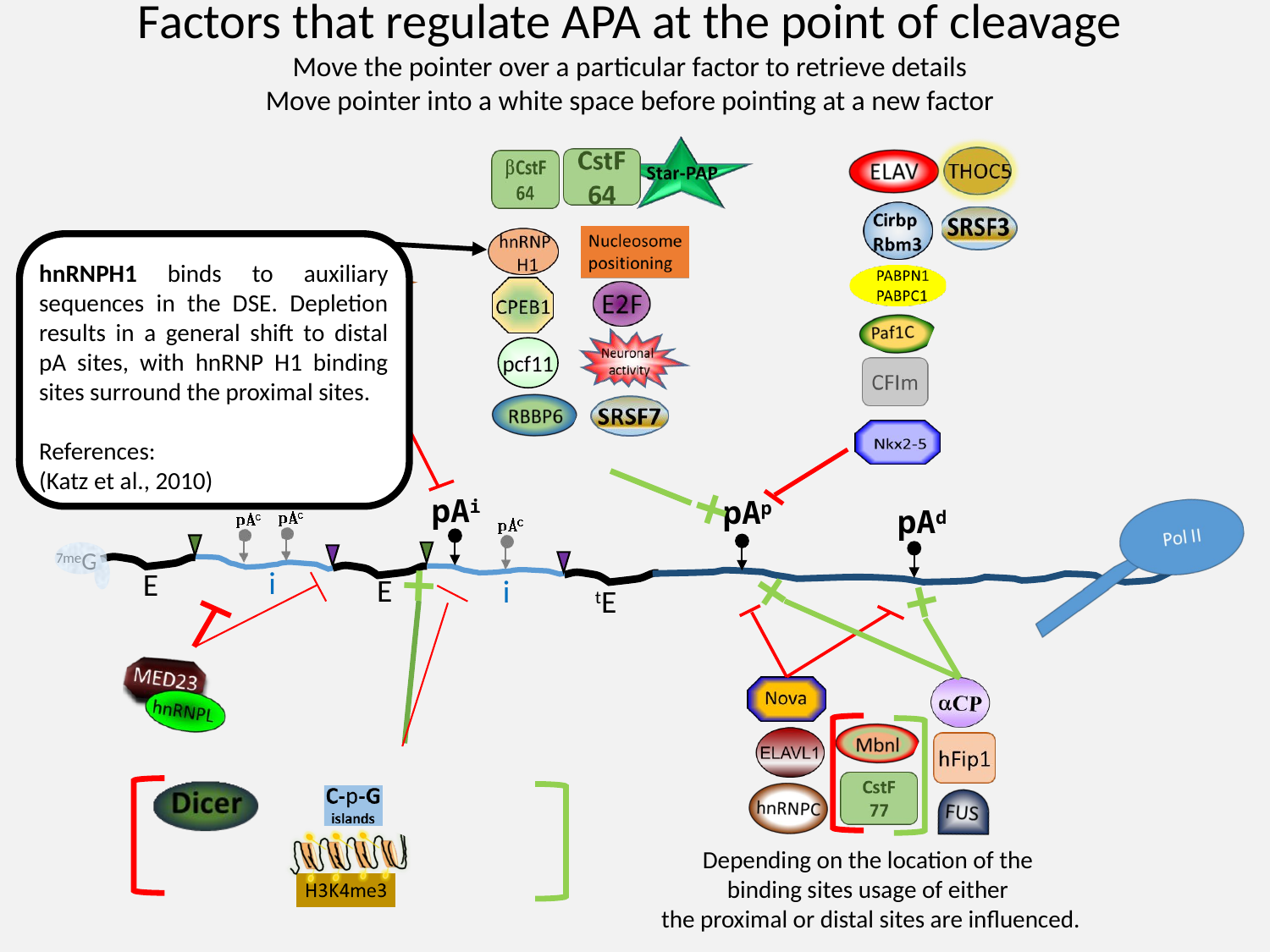

Factors that regulate APA at the point of cleavage
Move the pointer over a particular factor to retrieve details
Move pointer into a white space before pointing at a new factor
hnRNPH1 binds to auxiliary sequences in the DSE. Depletion results in a general shift to distal pA sites, with hnRNP H1 binding sites surround the proximal sites.
References:
(Katz et al., 2010)
pcf11
+
pAi
pAp
pAd
+
7meG
+
+
i
E
E
i
tE
Depending on the location of the
binding sites usage of either
the proximal or distal sites are influenced.

## Slide 5
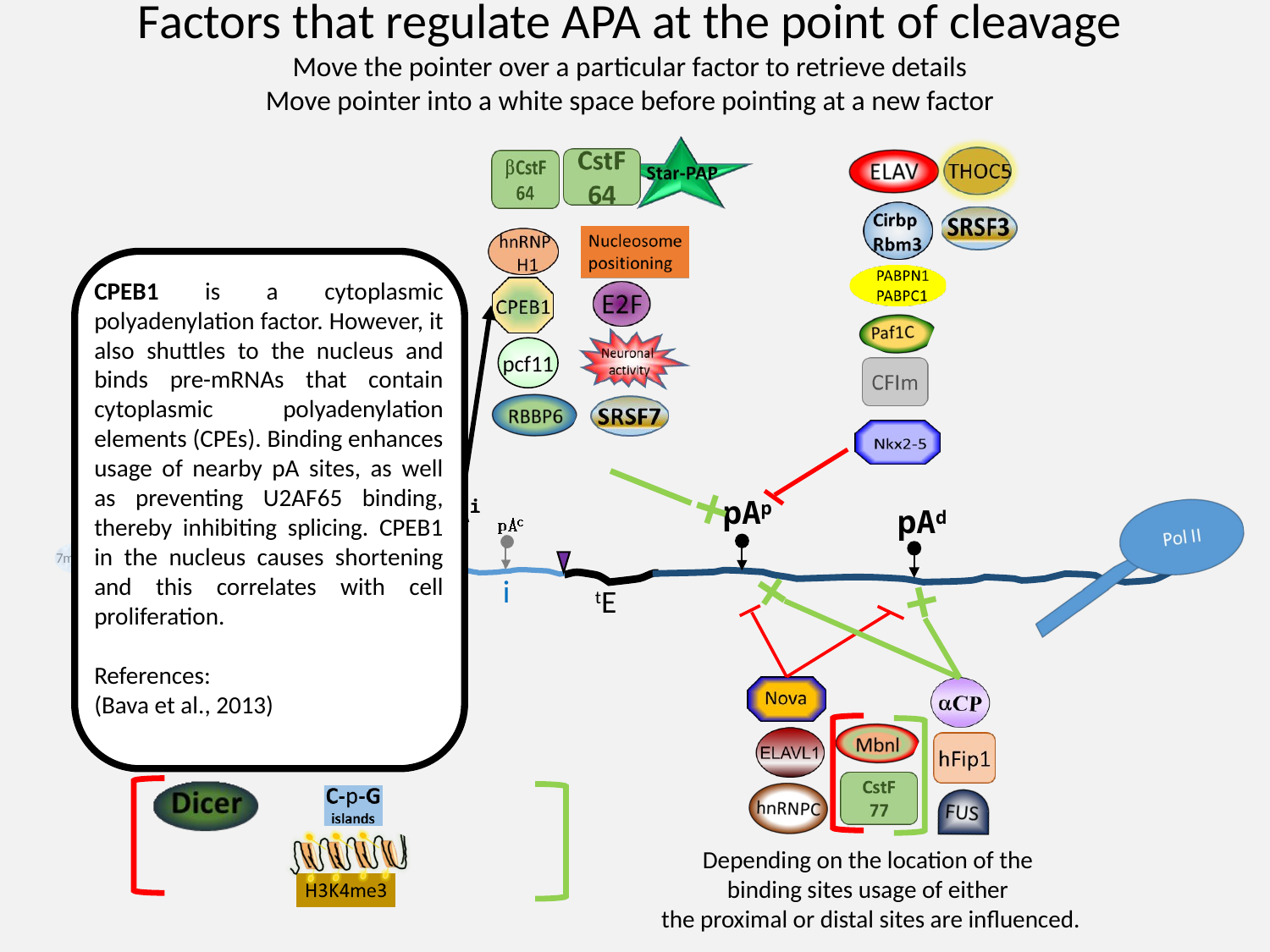

Factors that regulate APA at the point of cleavage
Move the pointer over a particular factor to retrieve details
Move pointer into a white space before pointing at a new factor
CPEB1 is a cytoplasmic polyadenylation factor. However, it also shuttles to the nucleus and binds pre-mRNAs that contain cytoplasmic polyadenylation elements (CPEs). Binding enhances usage of nearby pA sites, as well as preventing U2AF65 binding, thereby inhibiting splicing. CPEB1 in the nucleus causes shortening and this correlates with cell proliferation.
References:
(Bava et al., 2013)
pcf11
+
pAi
pAp
pAd
+
7meG
+
+
i
E
E
i
tE
Depending on the location of the
binding sites usage of either
the proximal or distal sites are influenced.

## Slide 6
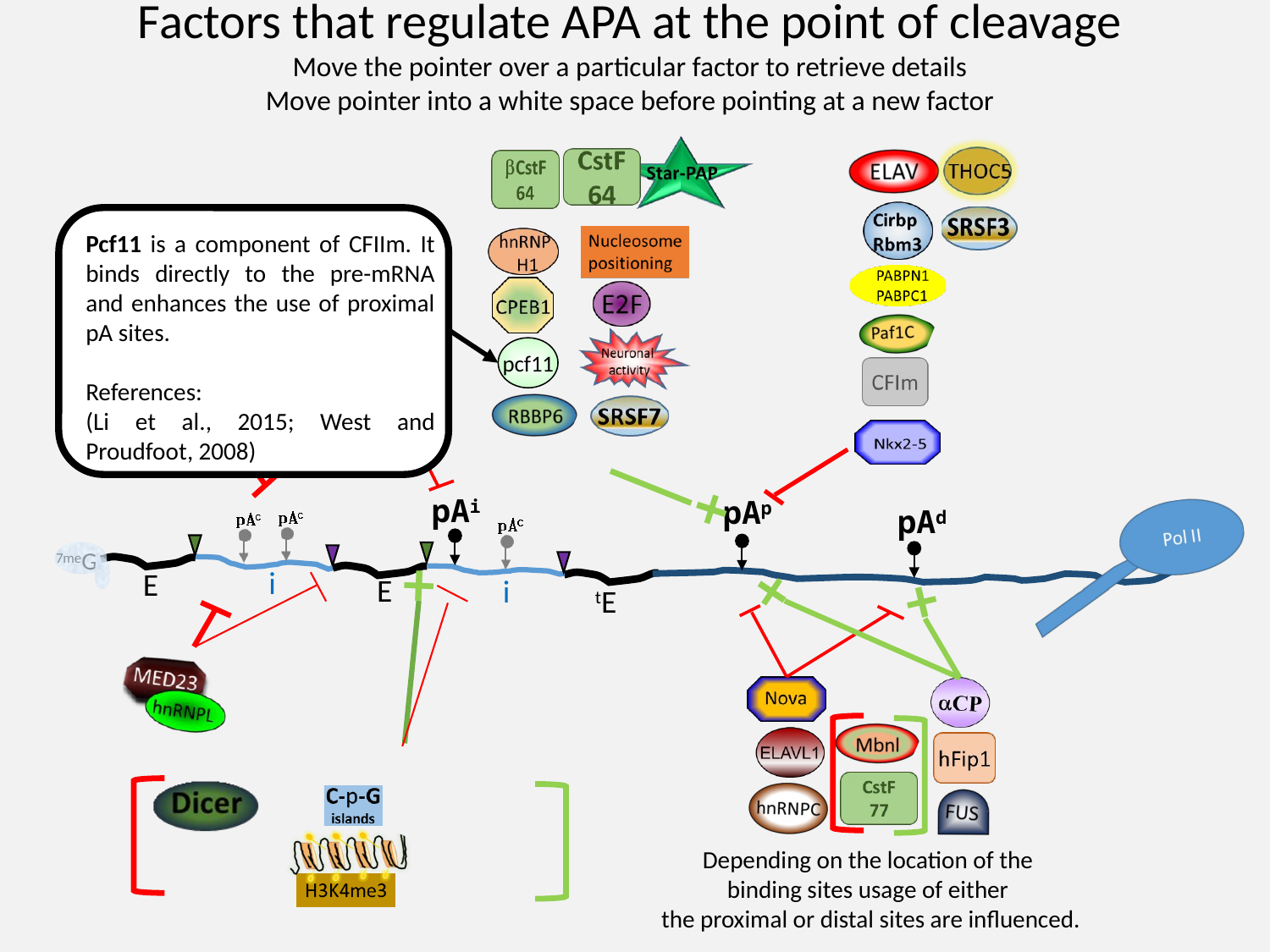

Factors that regulate APA at the point of cleavage
Move the pointer over a particular factor to retrieve details
Move pointer into a white space before pointing at a new factor
Pcf11 is a component of CFIIm. It binds directly to the pre-mRNA and enhances the use of proximal pA sites.
References:
(Li et al., 2015; West and Proudfoot, 2008)
pcf11
+
pAi
pAp
pAd
+
7meG
+
+
i
E
E
i
tE
Depending on the location of the
binding sites usage of either
the proximal or distal sites are influenced.

## Slide 7
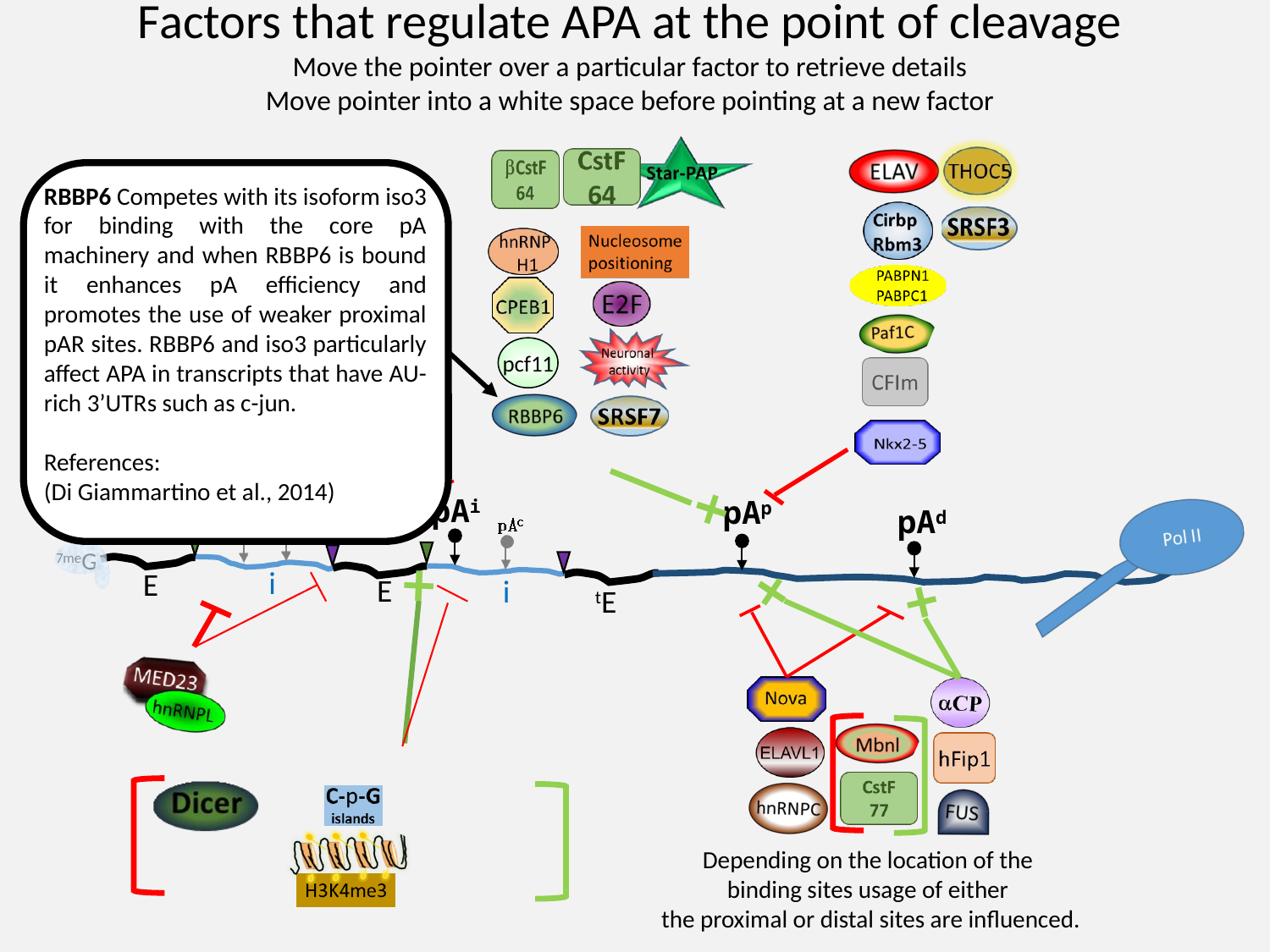

Factors that regulate APA at the point of cleavage
Move the pointer over a particular factor to retrieve details
Move pointer into a white space before pointing at a new factor
RBBP6 Competes with its isoform iso3 for binding with the core pA machinery and when RBBP6 is bound it enhances pA efficiency and promotes the use of weaker proximal pAR sites. RBBP6 and iso3 particularly affect APA in transcripts that have AU-rich 3’UTRs such as c-jun.
References:
(Di Giammartino et al., 2014)
pcf11
+
pAi
pAp
pAd
+
7meG
+
+
i
E
E
i
tE
Depending on the location of the
binding sites usage of either
the proximal or distal sites are influenced.

## Slide 8
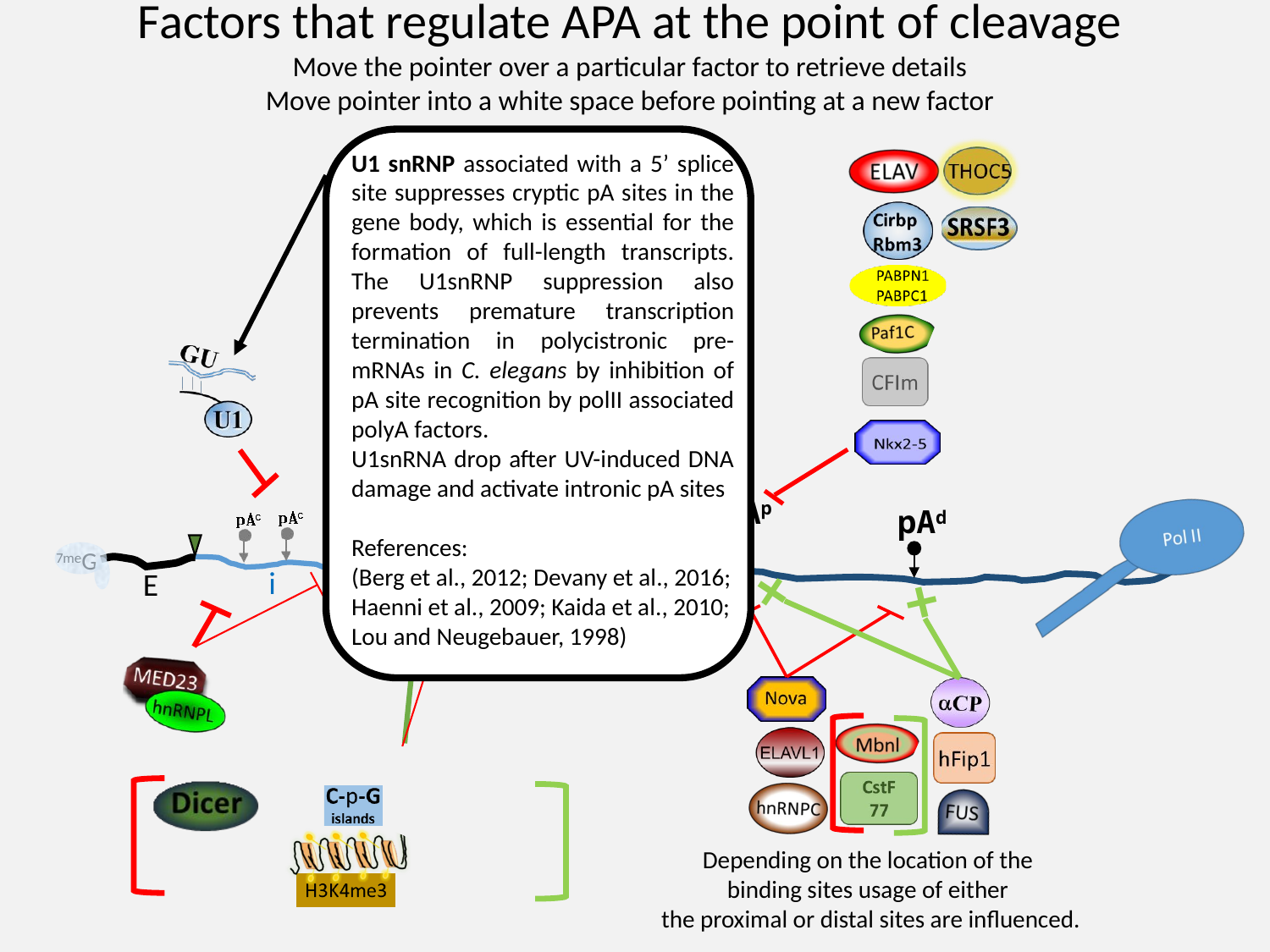

Factors that regulate APA at the point of cleavage
Move the pointer over a particular factor to retrieve details
Move pointer into a white space before pointing at a new factor
U1 snRNP associated with a 5’ splice site suppresses cryptic pA sites in the gene body, which is essential for the formation of full-length transcripts. The U1snRNP suppression also prevents premature transcription termination in polycistronic pre-mRNAs in C. elegans by inhibition of pA site recognition by polII associated polyA factors.
U1snRNA drop after UV-induced DNA damage and activate intronic pA sites
References:
(Berg et al., 2012; Devany et al., 2016; Haenni et al., 2009; Kaida et al., 2010; Lou and Neugebauer, 1998)
pcf11
+
pAi
pAp
pAd
+
7meG
+
+
i
E
E
i
tE
Depending on the location of the
binding sites usage of either
the proximal or distal sites are influenced.

## Slide 9
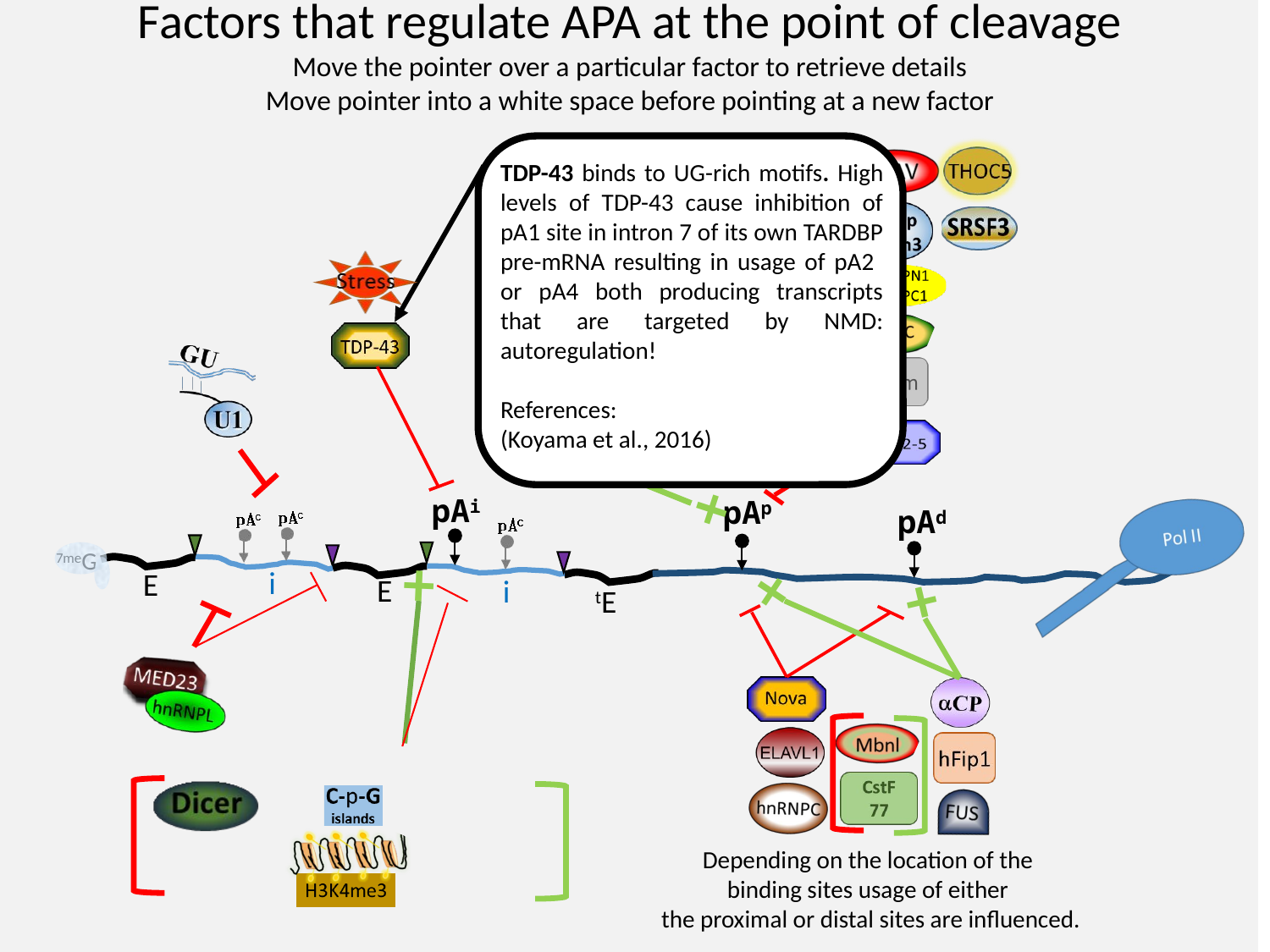

Factors that regulate APA at the point of cleavage
Move the pointer over a particular factor to retrieve details
Move pointer into a white space before pointing at a new factor
TDP-43 binds to UG-rich motifs. High levels of TDP-43 cause inhibition of pA1 site in intron 7 of its own TARDBP pre-mRNA resulting in usage of pA2 or pA4 both producing transcripts that are targeted by NMD: autoregulation!
References:
(Koyama et al., 2016)
pcf11
+
pAi
pAp
pAd
+
7meG
+
+
i
E
E
i
tE
Depending on the location of the
binding sites usage of either
the proximal or distal sites are influenced.

## Slide 10
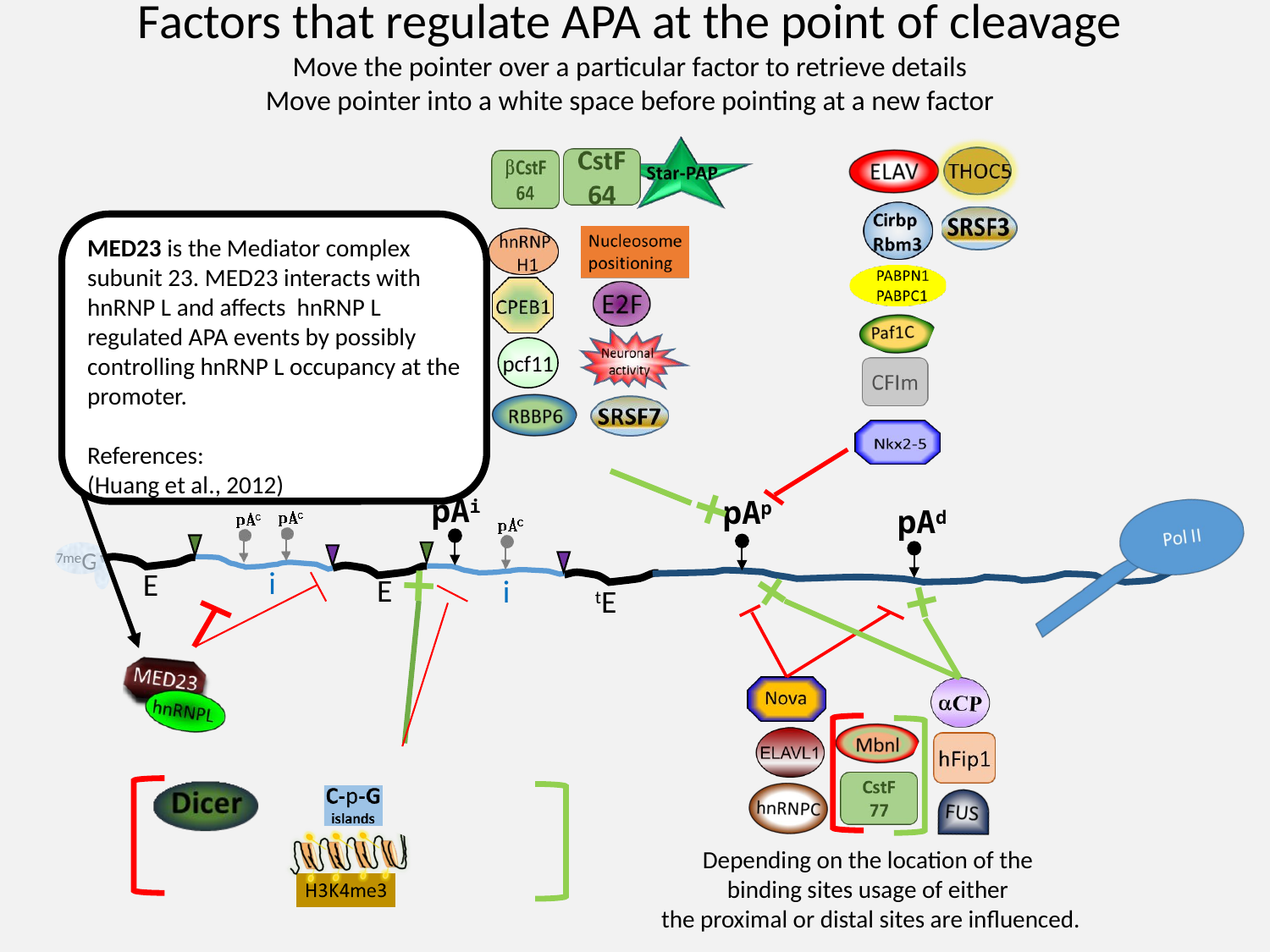

Factors that regulate APA at the point of cleavage
Move the pointer over a particular factor to retrieve details
Move pointer into a white space before pointing at a new factor
MED23 is the Mediator complex subunit 23. MED23 interacts with hnRNP L and affects hnRNP L regulated APA events by possibly controlling hnRNP L occupancy at the promoter.
References:
(Huang et al., 2012)
pcf11
+
pAi
pAp
pAd
+
7meG
+
+
i
E
E
i
tE
Depending on the location of the
binding sites usage of either
the proximal or distal sites are influenced.

## Slide 11
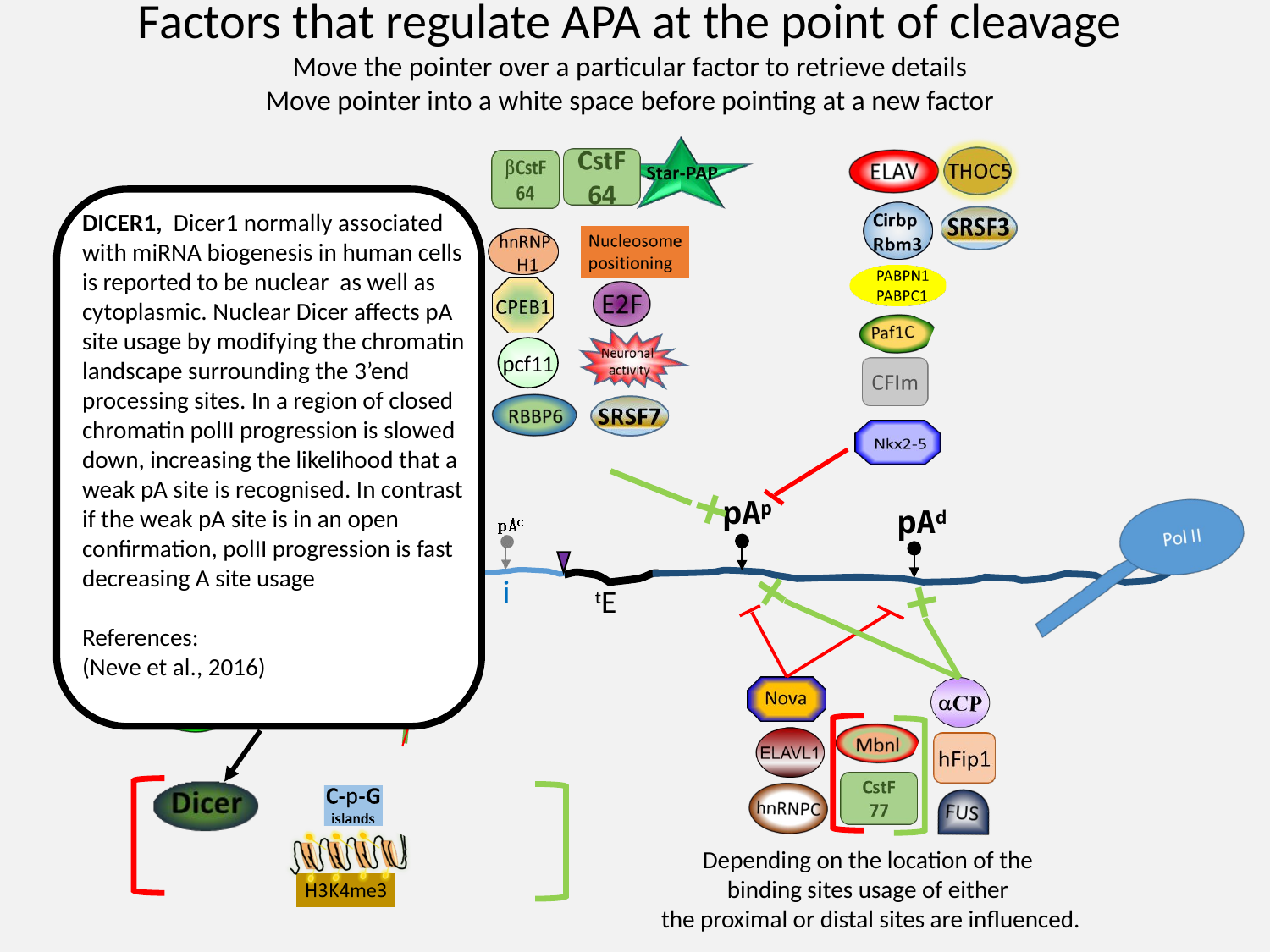

Factors that regulate APA at the point of cleavage
Move the pointer over a particular factor to retrieve details
Move pointer into a white space before pointing at a new factor
DICER1, Dicer1 normally associated with miRNA biogenesis in human cells is reported to be nuclear as well as cytoplasmic. Nuclear Dicer affects pA site usage by modifying the chromatin landscape surrounding the 3’end processing sites. In a region of closed chromatin polII progression is slowed down, increasing the likelihood that a weak pA site is recognised. In contrast if the weak pA site is in an open confirmation, polII progression is fast decreasing A site usage
References:
(Neve et al., 2016)
pcf11
+
pAi
pAp
pAd
+
7meG
+
+
i
E
E
i
tE
Depending on the location of the
binding sites usage of either
the proximal or distal sites are influenced.

## Slide 12
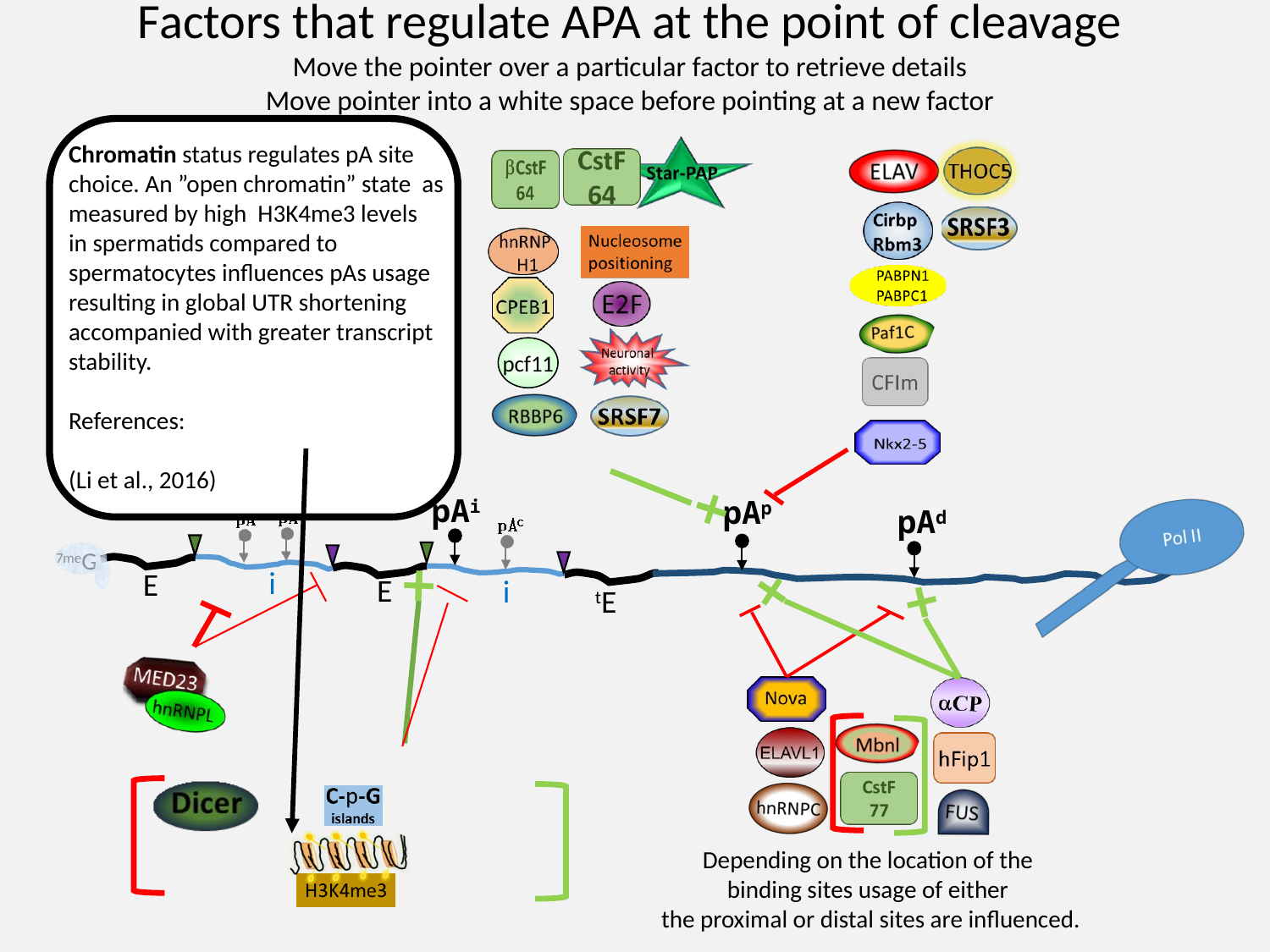

Factors that regulate APA at the point of cleavage
Move the pointer over a particular factor to retrieve details
Move pointer into a white space before pointing at a new factor
Chromatin status regulates pA site choice. An ”open chromatin” state as measured by high H3K4me3 levels
in spermatids compared to spermatocytes influences pAs usage resulting in global UTR shortening accompanied with greater transcript stability.
References:
(Li et al., 2016)
pcf11
+
pAi
pAp
pAd
+
7meG
+
+
i
E
E
i
tE
Depending on the location of the
binding sites usage of either
the proximal or distal sites are influenced.

## Slide 13
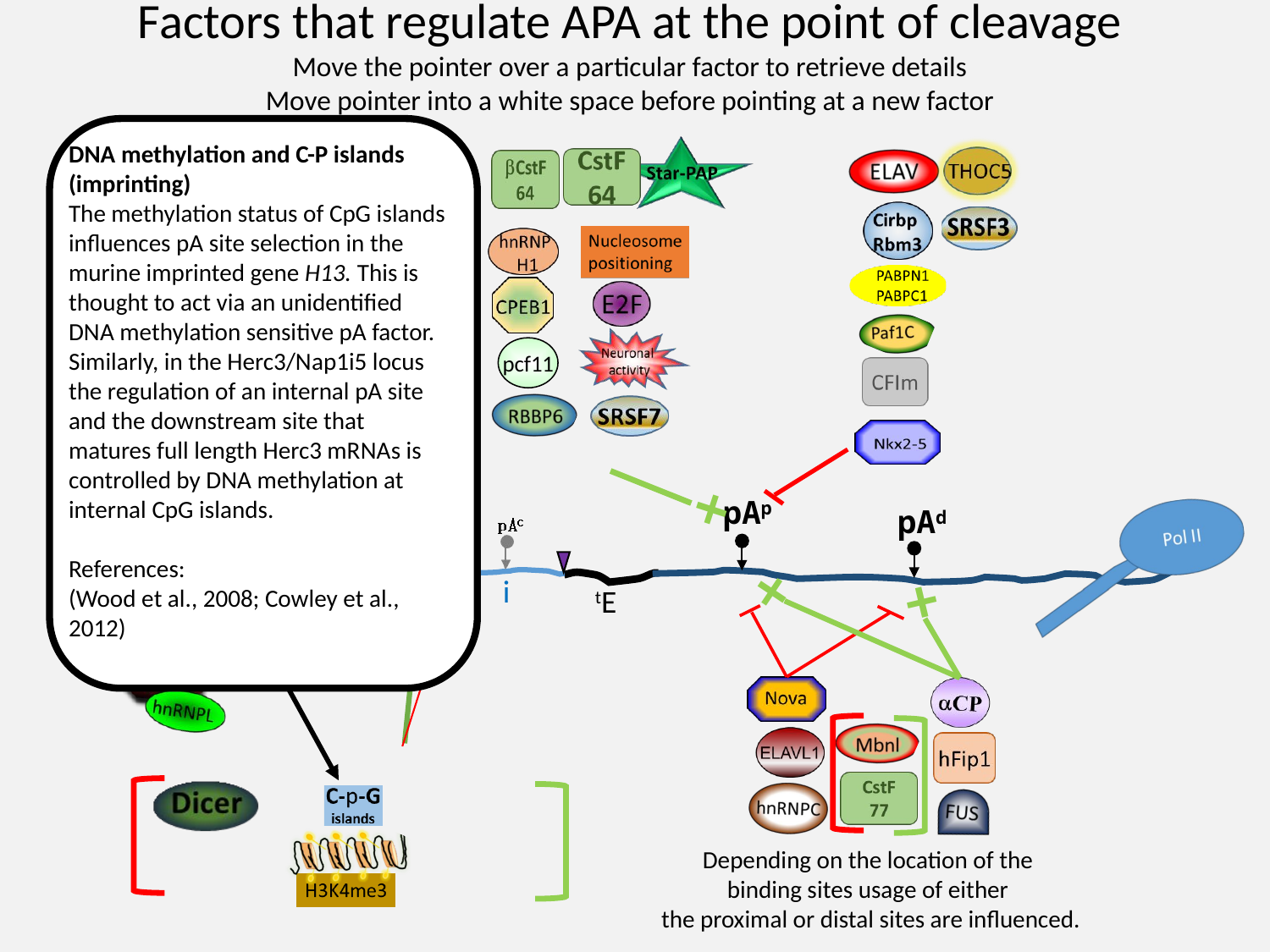

Factors that regulate APA at the point of cleavage
Move the pointer over a particular factor to retrieve details
Move pointer into a white space before pointing at a new factor
DNA methylation and C-P islands (imprinting)
The methylation status of CpG islands influences pA site selection in the murine imprinted gene H13. This is thought to act via an unidentified DNA methylation sensitive pA factor. Similarly, in the Herc3/Nap1i5 locus the regulation of an internal pA site and the downstream site that matures full length Herc3 mRNAs is controlled by DNA methylation at internal CpG islands.
References:
(Wood et al., 2008; Cowley et al., 2012)
pcf11
+
pAi
pAp
pAd
+
7meG
+
+
i
E
E
i
tE
Depending on the location of the
binding sites usage of either
the proximal or distal sites are influenced.

## Slide 14
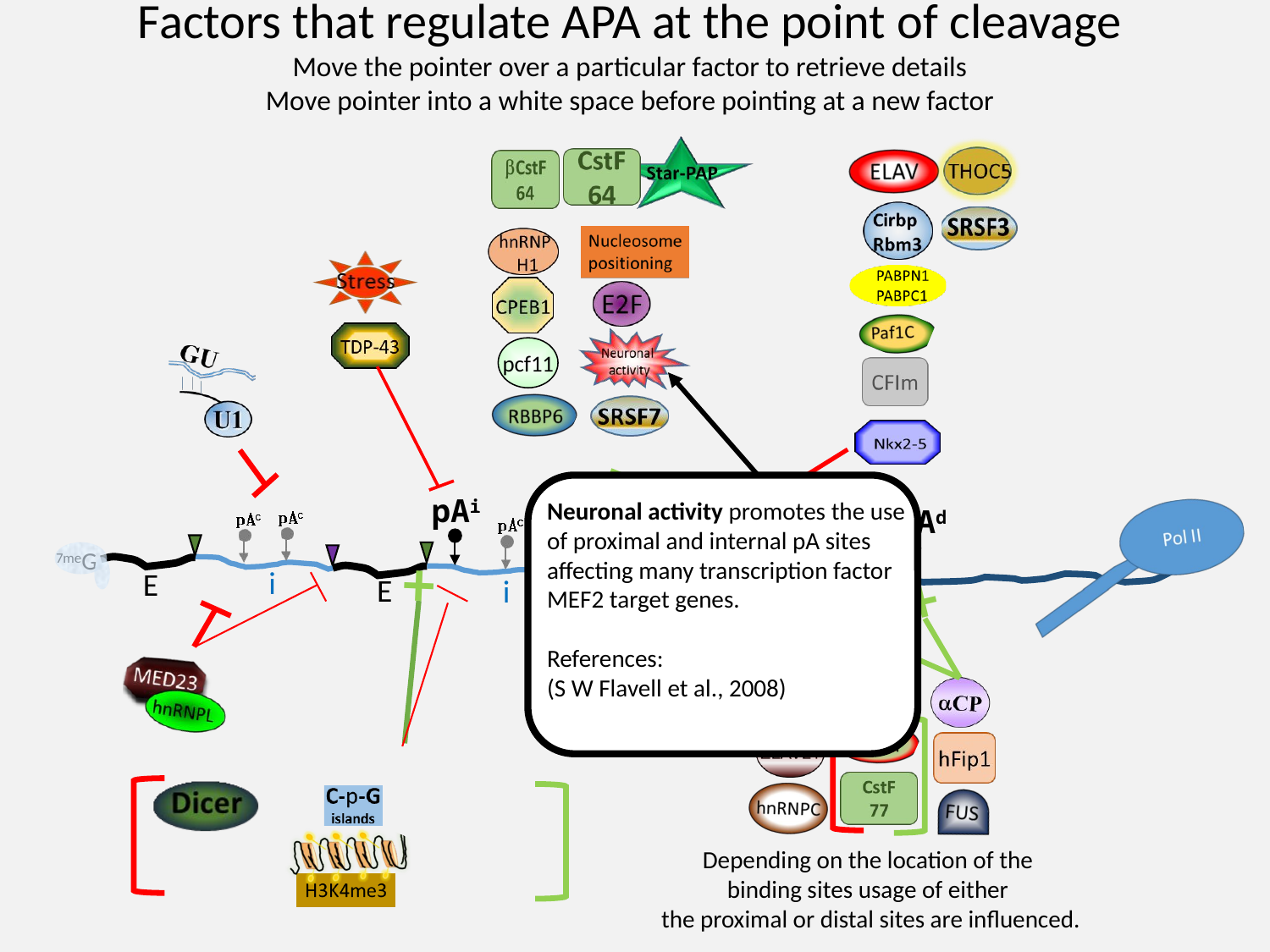

Factors that regulate APA at the point of cleavage
Move the pointer over a particular factor to retrieve details
Move pointer into a white space before pointing at a new factor
pcf11
+
pAi
pAp
Neuronal activity promotes the use of proximal and internal pA sites affecting many transcription factor MEF2 target genes.
References:
(S W Flavell et al., 2008)
pAd
+
7meG
+
+
i
E
E
i
tE
Depending on the location of the
binding sites usage of either
the proximal or distal sites are influenced.

## Slide 15
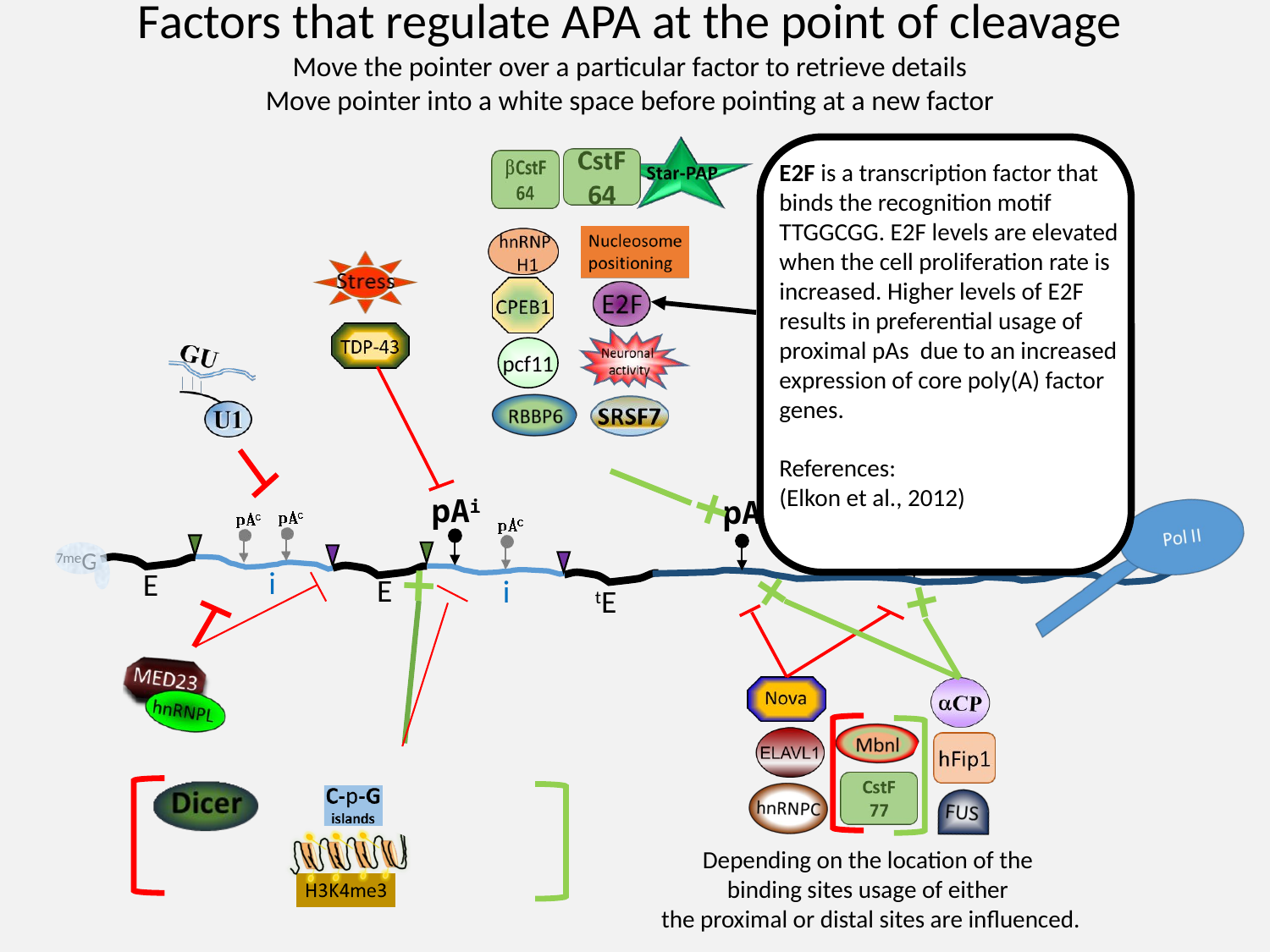

Factors that regulate APA at the point of cleavage
Move the pointer over a particular factor to retrieve details
Move pointer into a white space before pointing at a new factor
E2F is a transcription factor that binds the recognition motif TTGGCGG. E2F levels are elevated when the cell proliferation rate is increased. Higher levels of E2F results in preferential usage of proximal pAs due to an increased expression of core poly(A) factor genes.
References:
(Elkon et al., 2012)
pcf11
+
pAi
pAp
pAd
+
7meG
+
+
i
E
E
i
tE
Depending on the location of the
binding sites usage of either
the proximal or distal sites are influenced.

## Slide 16
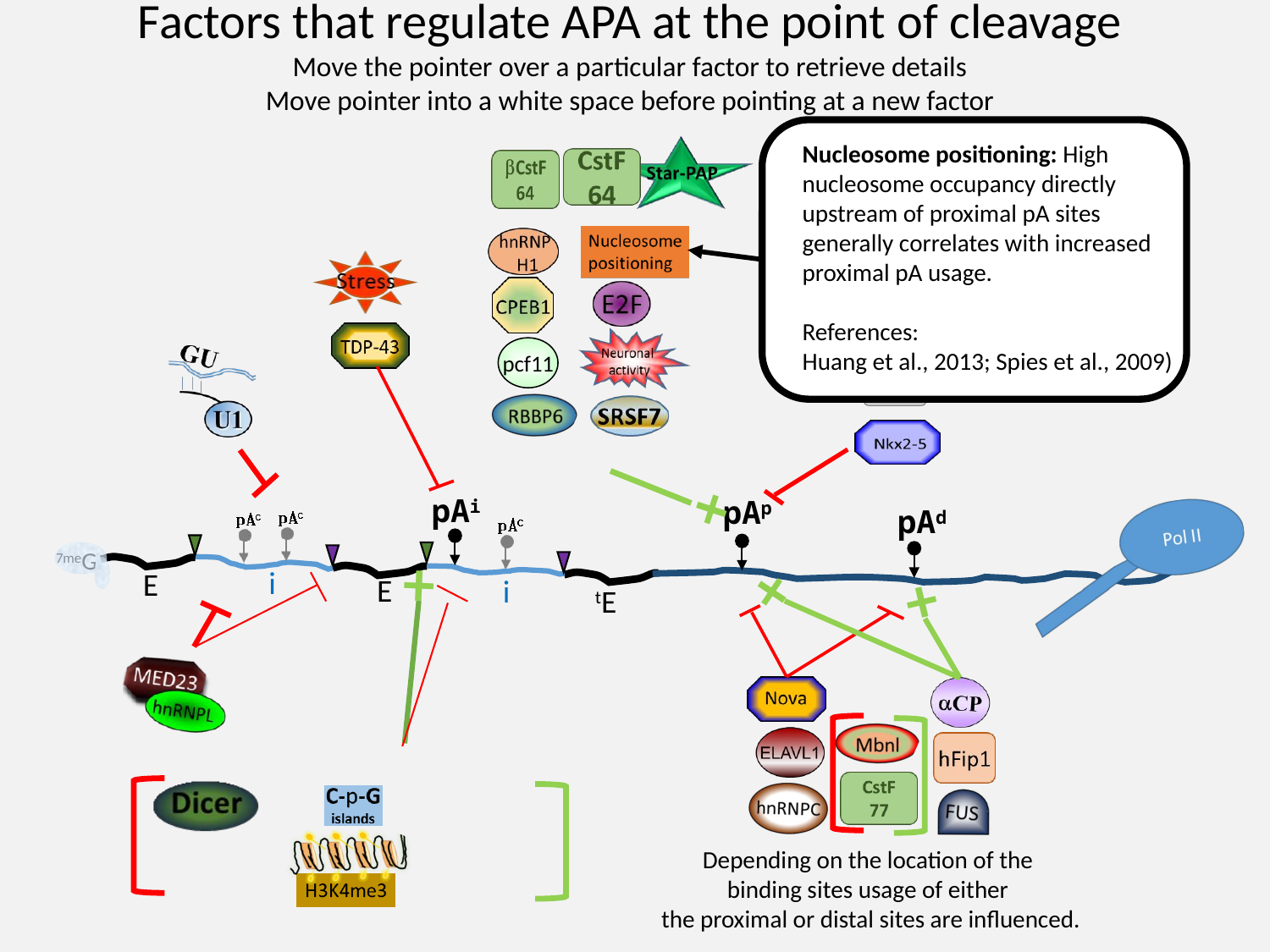

Factors that regulate APA at the point of cleavage
Move the pointer over a particular factor to retrieve details
Move pointer into a white space before pointing at a new factor
Nucleosome positioning: High nucleosome occupancy directly upstream of proximal pA sites generally correlates with increased proximal pA usage.
References:
Huang et al., 2013; Spies et al., 2009)
pcf11
+
pAi
pAp
pAd
+
7meG
+
+
i
E
E
i
tE
Depending on the location of the
binding sites usage of either
the proximal or distal sites are influenced.

## Slide 17
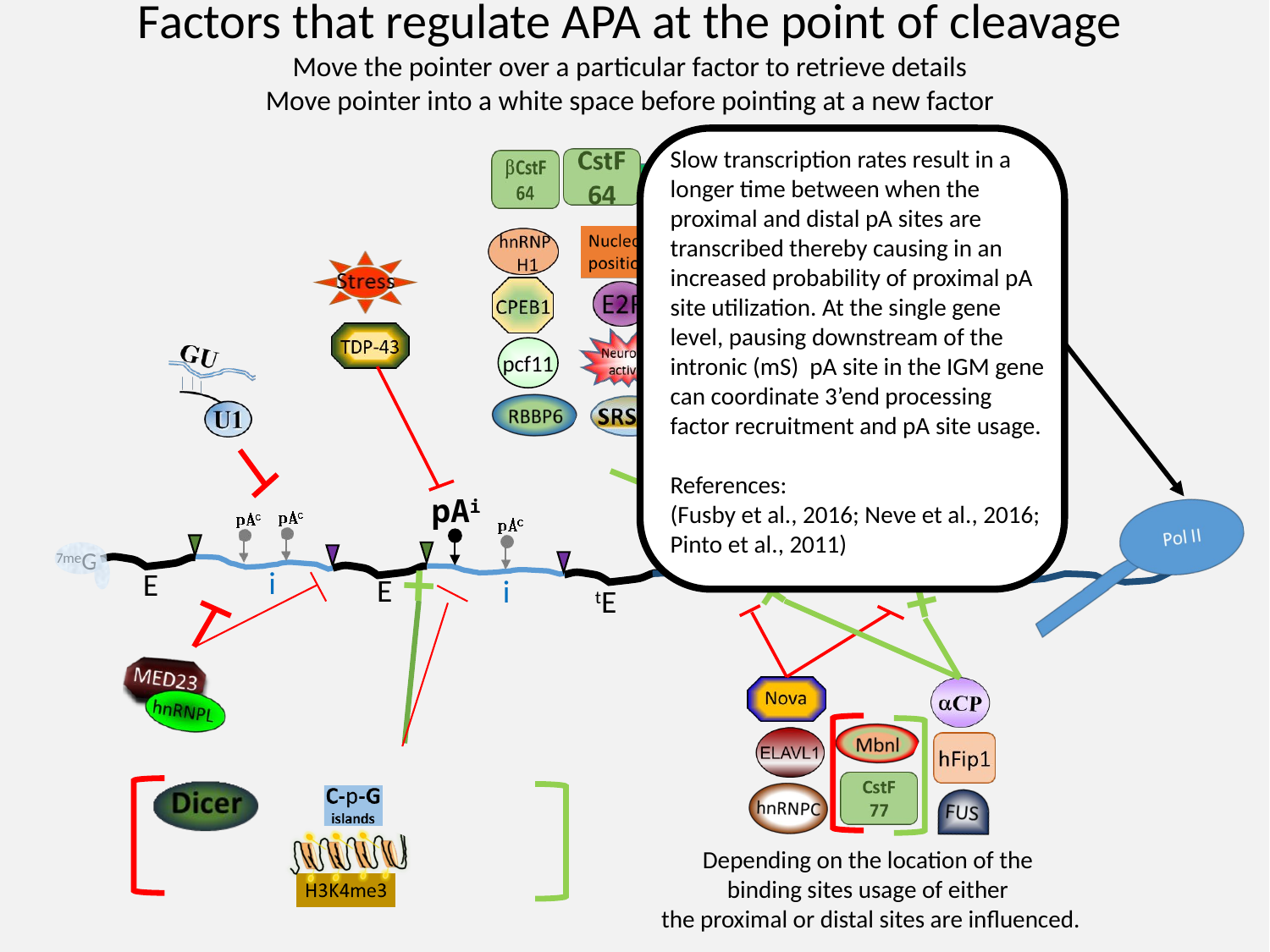

Factors that regulate APA at the point of cleavage
Move the pointer over a particular factor to retrieve details
Move pointer into a white space before pointing at a new factor
Slow transcription rates result in a longer time between when the proximal and distal pA sites are transcribed thereby causing in an increased probability of proximal pA site utilization. At the single gene level, pausing downstream of the intronic (mS) pA site in the IGM gene can coordinate 3’end processing factor recruitment and pA site usage.
References:
(Fusby et al., 2016; Neve et al., 2016; Pinto et al., 2011)
pcf11
+
pAi
pAp
pAd
+
7meG
+
+
i
E
E
i
tE
Depending on the location of the
binding sites usage of either
the proximal or distal sites are influenced.

## Slide 18
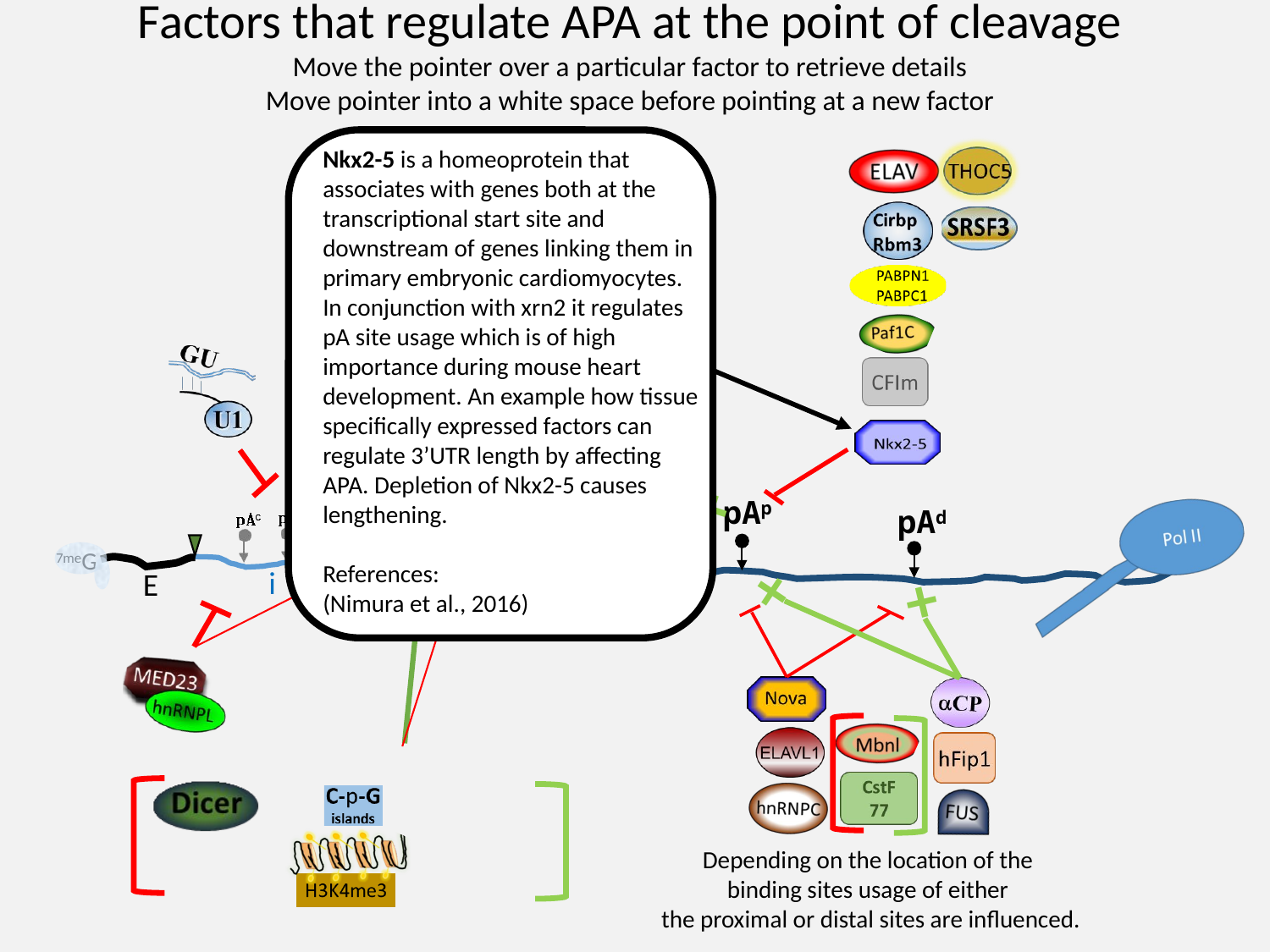

Factors that regulate APA at the point of cleavage
Move the pointer over a particular factor to retrieve details
Move pointer into a white space before pointing at a new factor
Nkx2-5 is a homeoprotein that associates with genes both at the transcriptional start site and downstream of genes linking them in primary embryonic cardiomyocytes. In conjunction with xrn2 it regulates pA site usage which is of high importance during mouse heart development. An example how tissue specifically expressed factors can regulate 3’UTR length by affecting APA. Depletion of Nkx2-5 causes lengthening.
References:
(Nimura et al., 2016)
pcf11
+
pAi
pAp
pAd
+
7meG
+
+
i
E
E
i
tE
Depending on the location of the
binding sites usage of either
the proximal or distal sites are influenced.

## Slide 19
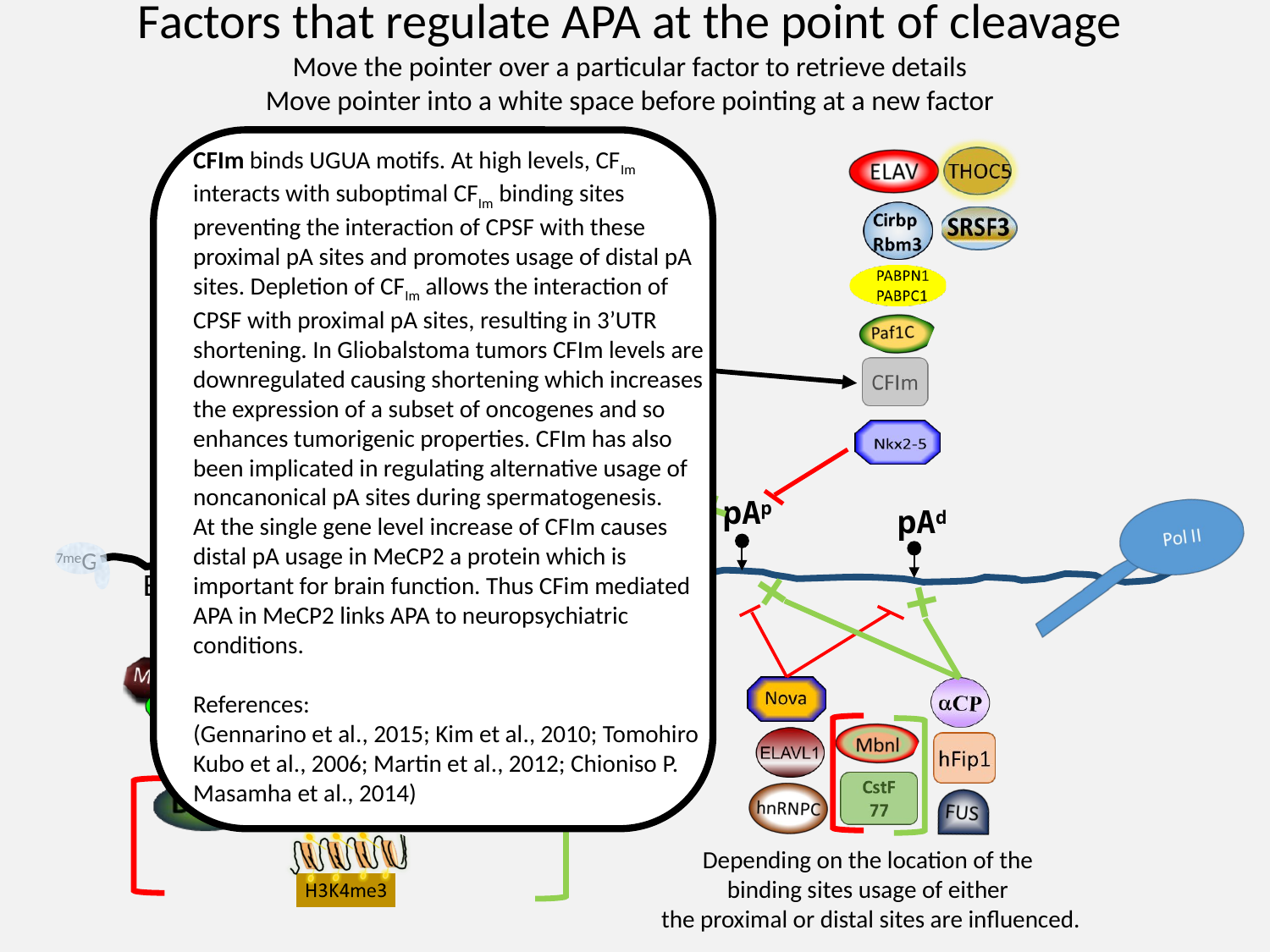

Factors that regulate APA at the point of cleavage
Move the pointer over a particular factor to retrieve details
Move pointer into a white space before pointing at a new factor
CFIm binds UGUA motifs. At high levels, CFIm interacts with suboptimal CFIm binding sites preventing the interaction of CPSF with these proximal pA sites and promotes usage of distal pA sites. Depletion of CFIm allows the interaction of CPSF with proximal pA sites, resulting in 3’UTR shortening. In Gliobalstoma tumors CFIm levels are downregulated causing shortening which increases the expression of a subset of oncogenes and so enhances tumorigenic properties. CFIm has also been implicated in regulating alternative usage of noncanonical pA sites during spermatogenesis.
At the single gene level increase of CFIm causes distal pA usage in MeCP2 a protein which is important for brain function. Thus CFim mediated APA in MeCP2 links APA to neuropsychiatric conditions.
References:
(Gennarino et al., 2015; Kim et al., 2010; Tomohiro Kubo et al., 2006; Martin et al., 2012; Chioniso P. Masamha et al., 2014)
pcf11
+
pAi
pAp
pAd
+
7meG
+
+
i
E
E
i
tE
Depending on the location of the
binding sites usage of either
the proximal or distal sites are influenced.

## Slide 20
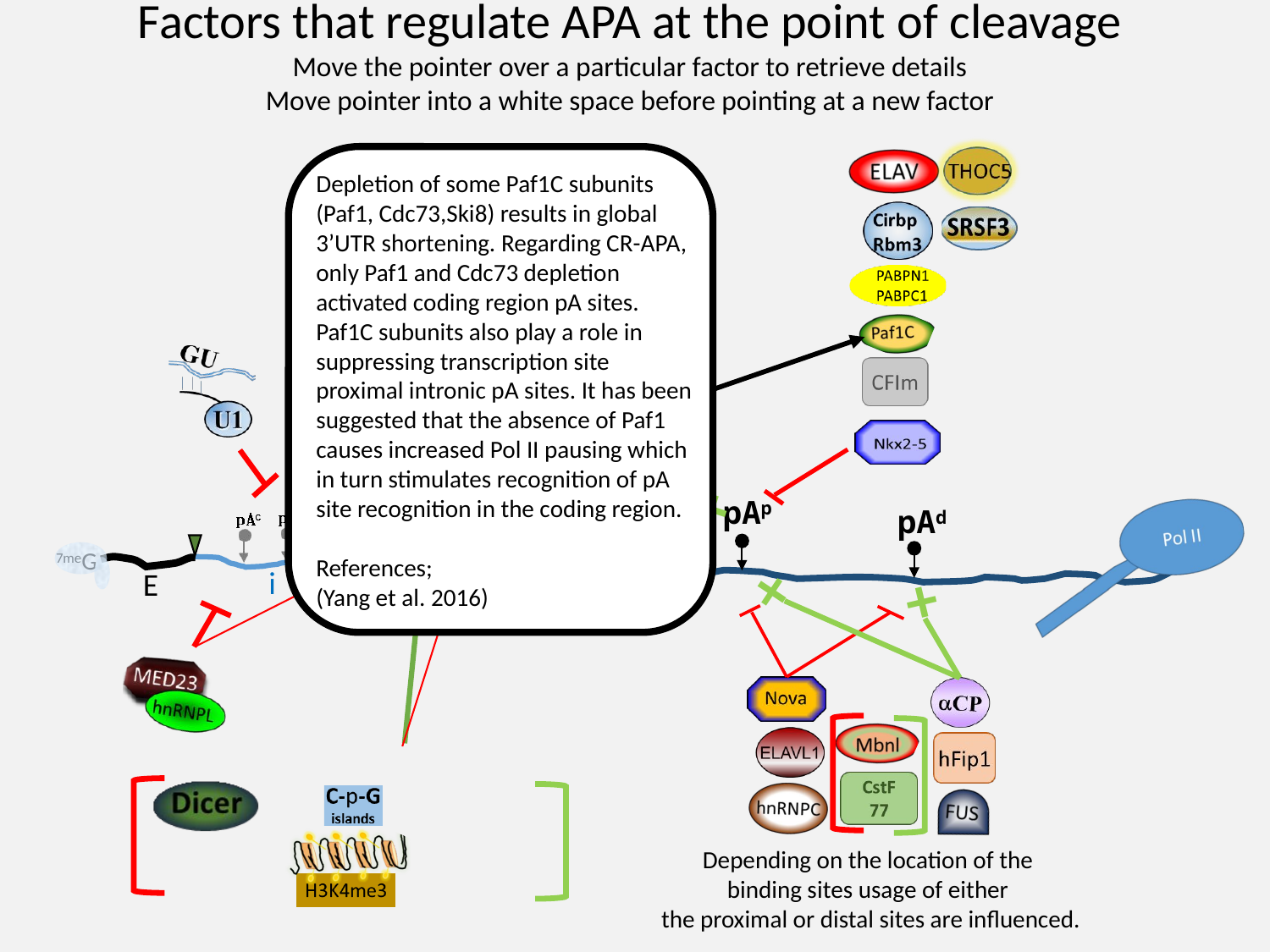

Factors that regulate APA at the point of cleavage
Move the pointer over a particular factor to retrieve details
Move pointer into a white space before pointing at a new factor
Depletion of some Paf1C subunits (Paf1, Cdc73,Ski8) results in global 3’UTR shortening. Regarding CR-APA, only Paf1 and Cdc73 depletion activated coding region pA sites. Paf1C subunits also play a role in suppressing transcription site proximal intronic pA sites. It has been suggested that the absence of Paf1 causes increased Pol II pausing which in turn stimulates recognition of pA site recognition in the coding region.
References;
(Yang et al. 2016)
pcf11
+
pAi
pAp
pAd
+
7meG
+
+
i
E
E
i
tE
Depending on the location of the
binding sites usage of either
the proximal or distal sites are influenced.

## Slide 21
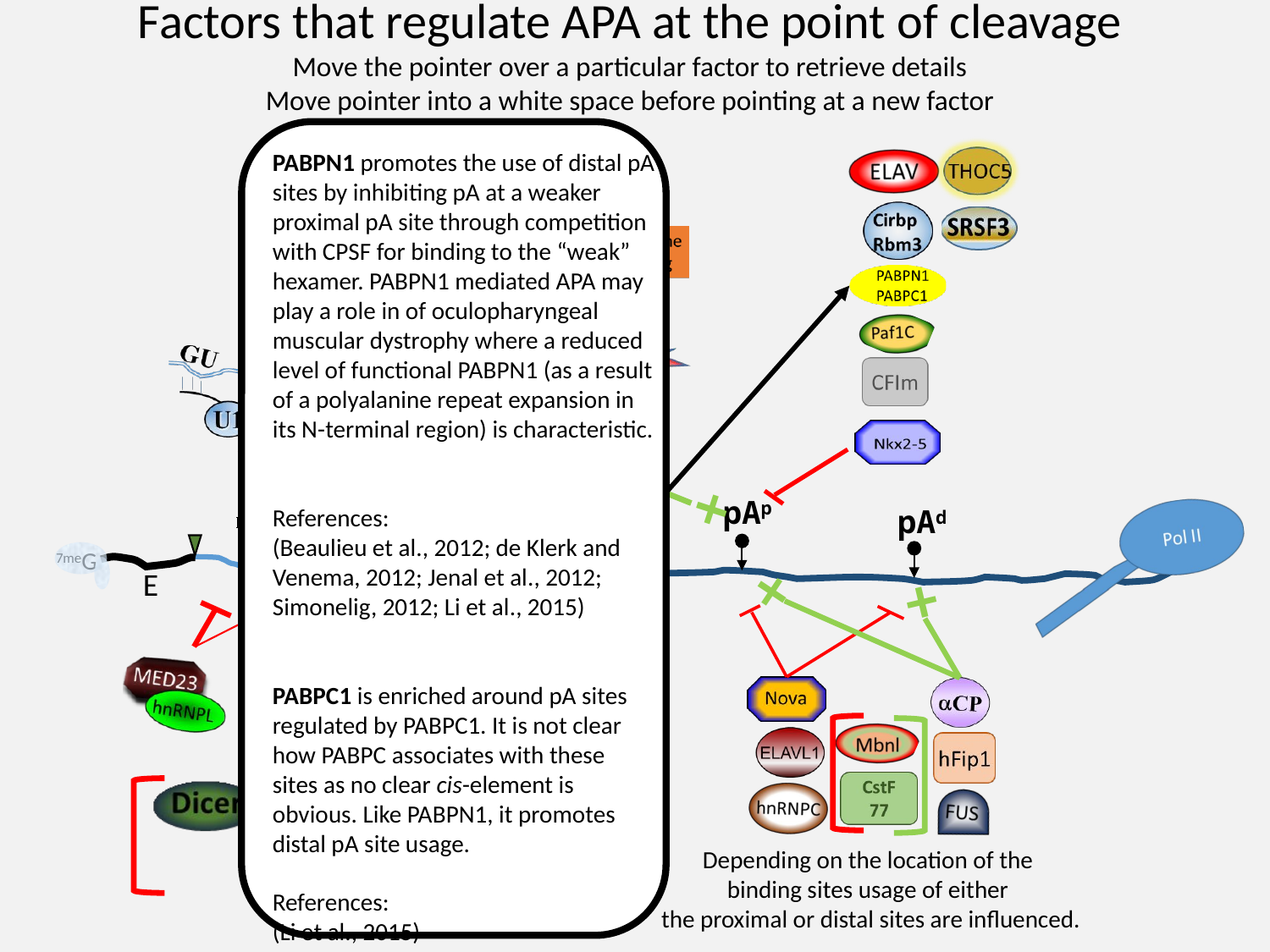

Factors that regulate APA at the point of cleavage
Move the pointer over a particular factor to retrieve details
Move pointer into a white space before pointing at a new factor
PABPN1 promotes the use of distal pA sites by inhibiting pA at a weaker proximal pA site through competition with CPSF for binding to the “weak” hexamer. PABPN1 mediated APA may play a role in of oculopharyngeal muscular dystrophy where a reduced level of functional PABPN1 (as a result of a polyalanine repeat expansion in its N-terminal region) is characteristic.
References:
(Beaulieu et al., 2012; de Klerk and Venema, 2012; Jenal et al., 2012; Simonelig, 2012; Li et al., 2015)
PABPC1 is enriched around pA sites regulated by PABPC1. It is not clear how PABPC associates with these sites as no clear cis-element is obvious. Like PABPN1, it promotes distal pA site usage.
References:
(Li et al., 2015)
pcf11
+
pAi
pAp
pAd
+
7meG
+
+
i
E
E
i
tE
Depending on the location of the
binding sites usage of either
the proximal or distal sites are influenced.

## Slide 22
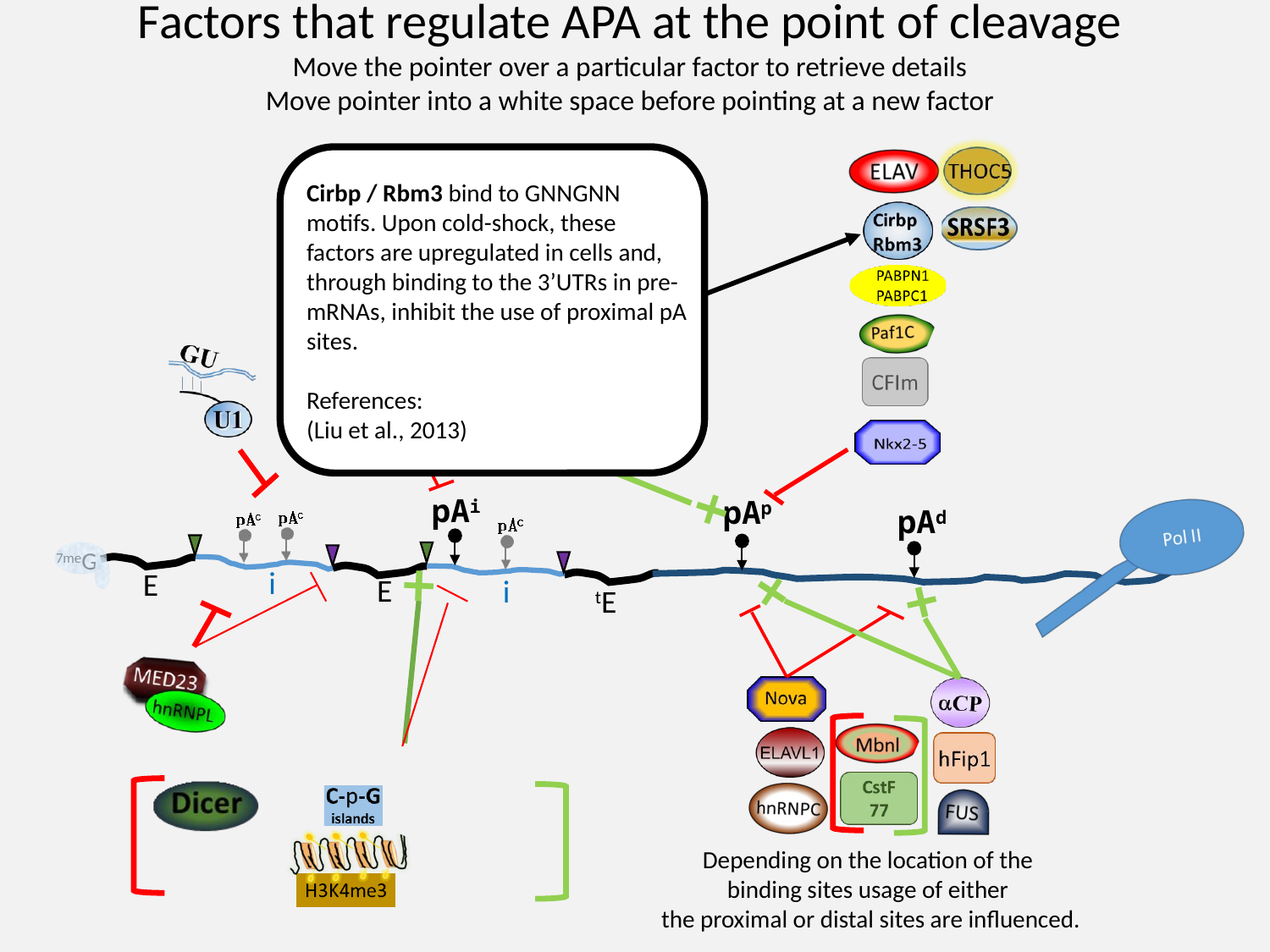

Factors that regulate APA at the point of cleavage
Move the pointer over a particular factor to retrieve details
Move pointer into a white space before pointing at a new factor
Cirbp / Rbm3 bind to GNNGNN motifs. Upon cold-shock, these factors are upregulated in cells and, through binding to the 3’UTRs in pre-mRNAs, inhibit the use of proximal pA sites.
References:
(Liu et al., 2013)
pcf11
+
pAi
pAp
pAd
+
7meG
+
+
i
E
E
i
tE
Depending on the location of the
binding sites usage of either
the proximal or distal sites are influenced.

## Slide 23
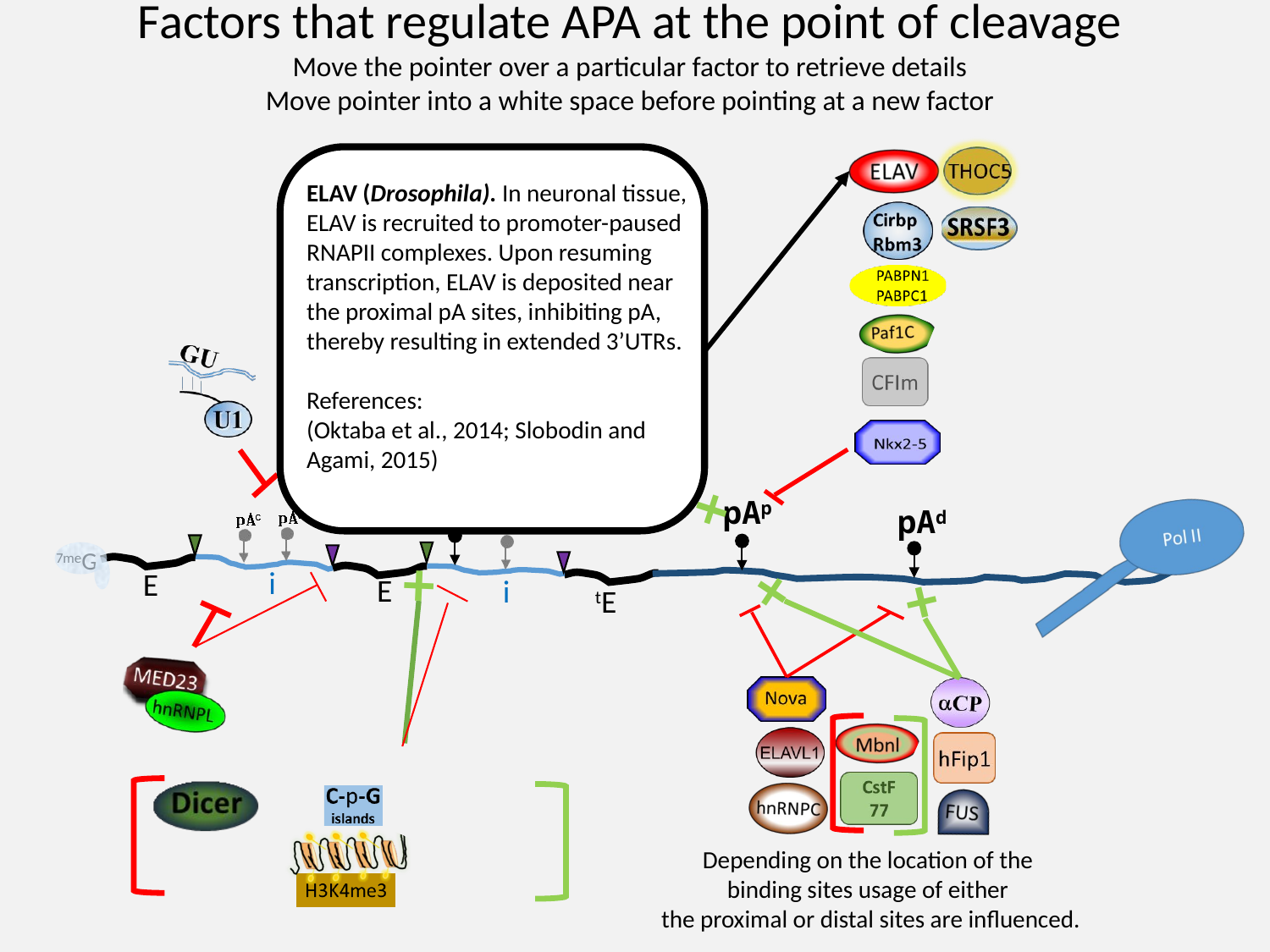

Factors that regulate APA at the point of cleavage
Move the pointer over a particular factor to retrieve details
Move pointer into a white space before pointing at a new factor
ELAV (Drosophila). In neuronal tissue, ELAV is recruited to promoter-paused RNAPII complexes. Upon resuming transcription, ELAV is deposited near the proximal pA sites, inhibiting pA, thereby resulting in extended 3’UTRs.
References:
(Oktaba et al., 2014; Slobodin and Agami, 2015)
pcf11
+
pAi
pAp
pAd
+
7meG
+
+
i
E
E
i
tE
Depending on the location of the
binding sites usage of either
the proximal or distal sites are influenced.

## Slide 24
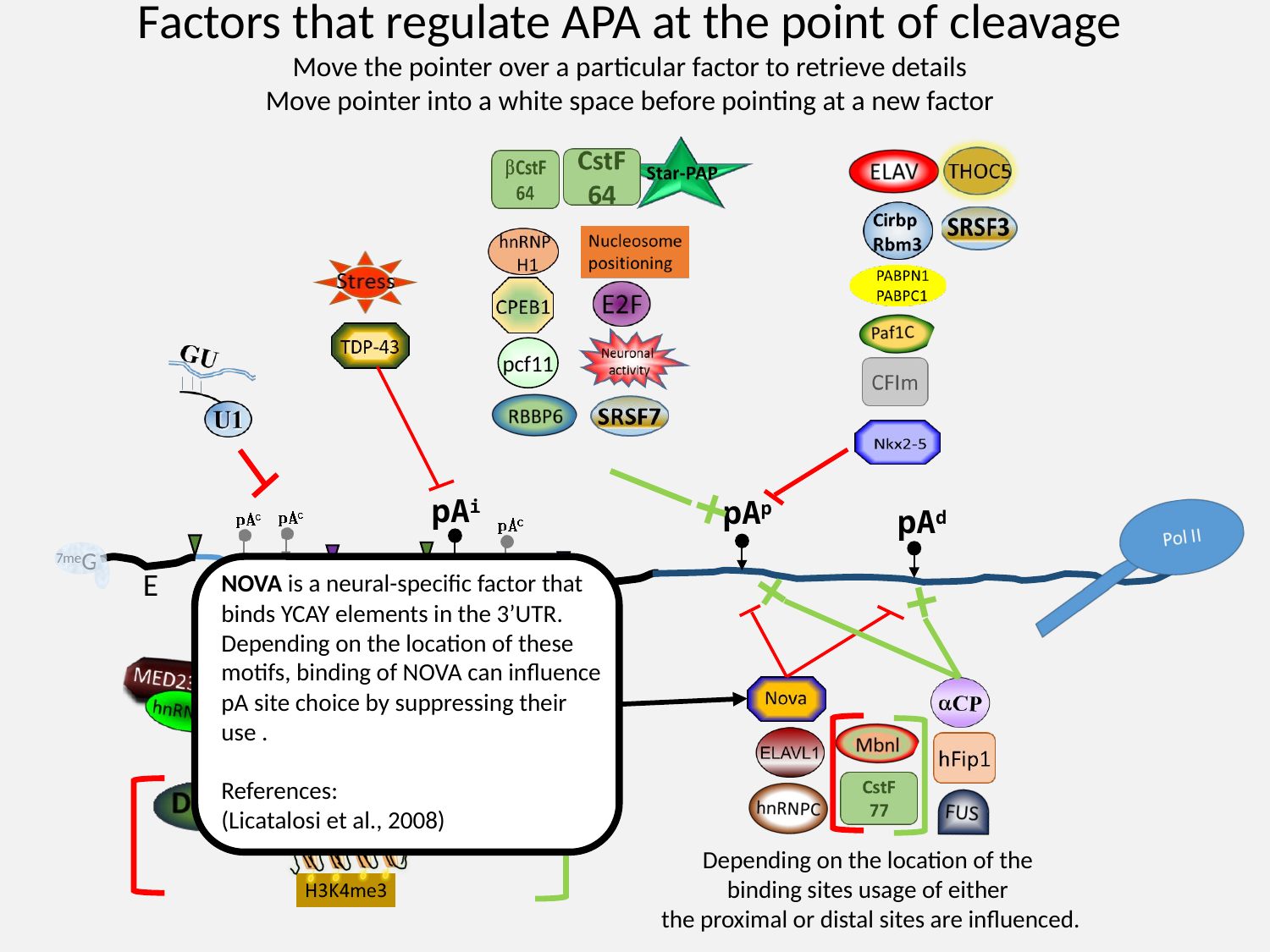

Factors that regulate APA at the point of cleavage
Move the pointer over a particular factor to retrieve details
Move pointer into a white space before pointing at a new factor
pcf11
+
pAi
pAp
pAd
+
7meG
+
+
i
E
NOVA is a neural-specific factor that binds YCAY elements in the 3’UTR. Depending on the location of these motifs, binding of NOVA can influence pA site choice by suppressing their use .
References:
(Licatalosi et al., 2008)
E
i
tE
Depending on the location of the
binding sites usage of either
the proximal or distal sites are influenced.

## Slide 25
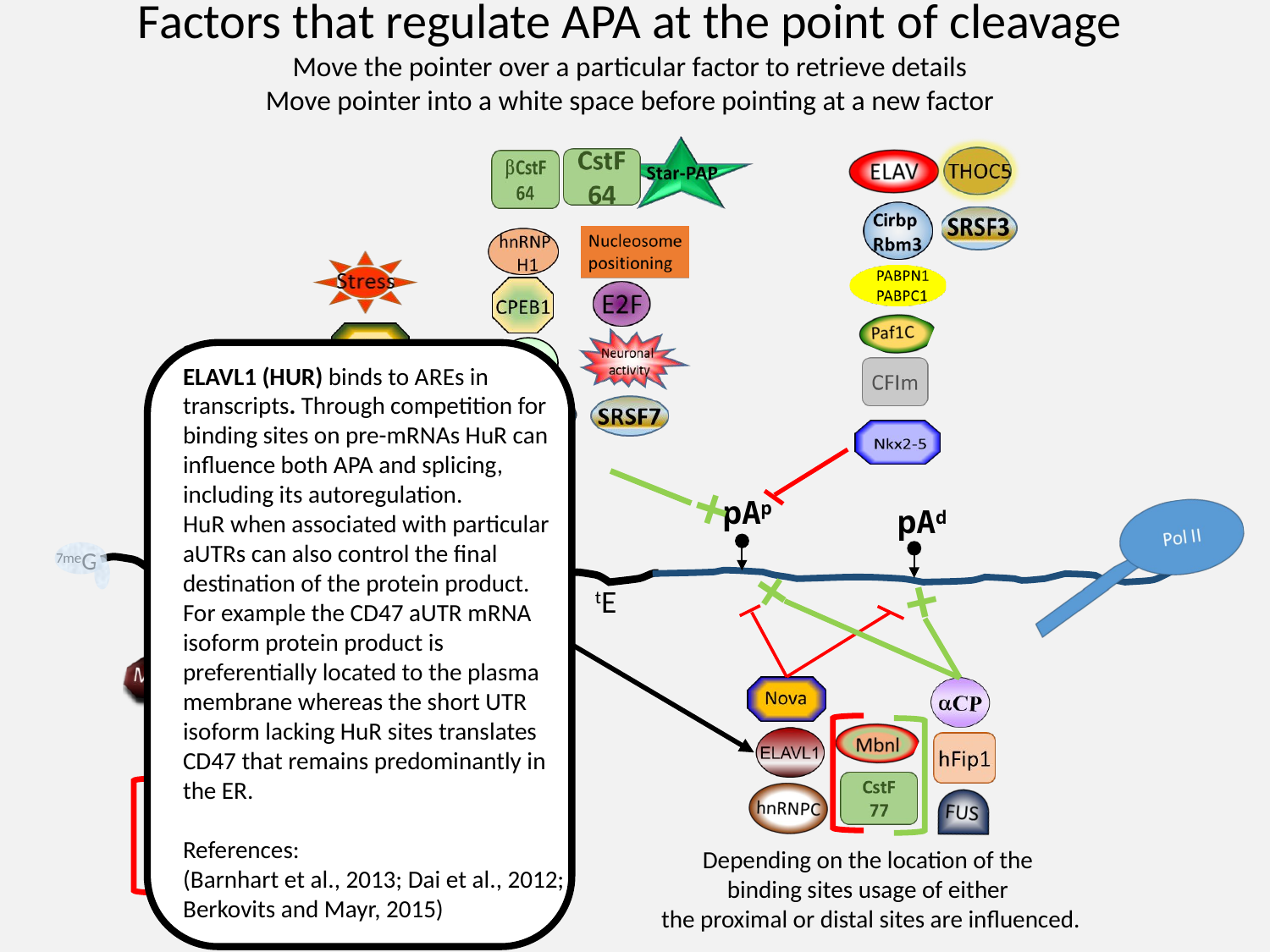

Factors that regulate APA at the point of cleavage
Move the pointer over a particular factor to retrieve details
Move pointer into a white space before pointing at a new factor
pcf11
ELAVL1 (HUR) binds to AREs in transcripts. Through competition for binding sites on pre-mRNAs HuR can influence both APA and splicing, including its autoregulation.
HuR when associated with particular aUTRs can also control the final destination of the protein product. For example the CD47 aUTR mRNA isoform protein product is preferentially located to the plasma membrane whereas the short UTR isoform lacking HuR sites translates CD47 that remains predominantly in the ER.
References:
(Barnhart et al., 2013; Dai et al., 2012; Berkovits and Mayr, 2015)
+
pAi
pAp
pAd
+
7meG
+
+
i
E
E
i
tE
Depending on the location of the
binding sites usage of either
the proximal or distal sites are influenced.

## Slide 26
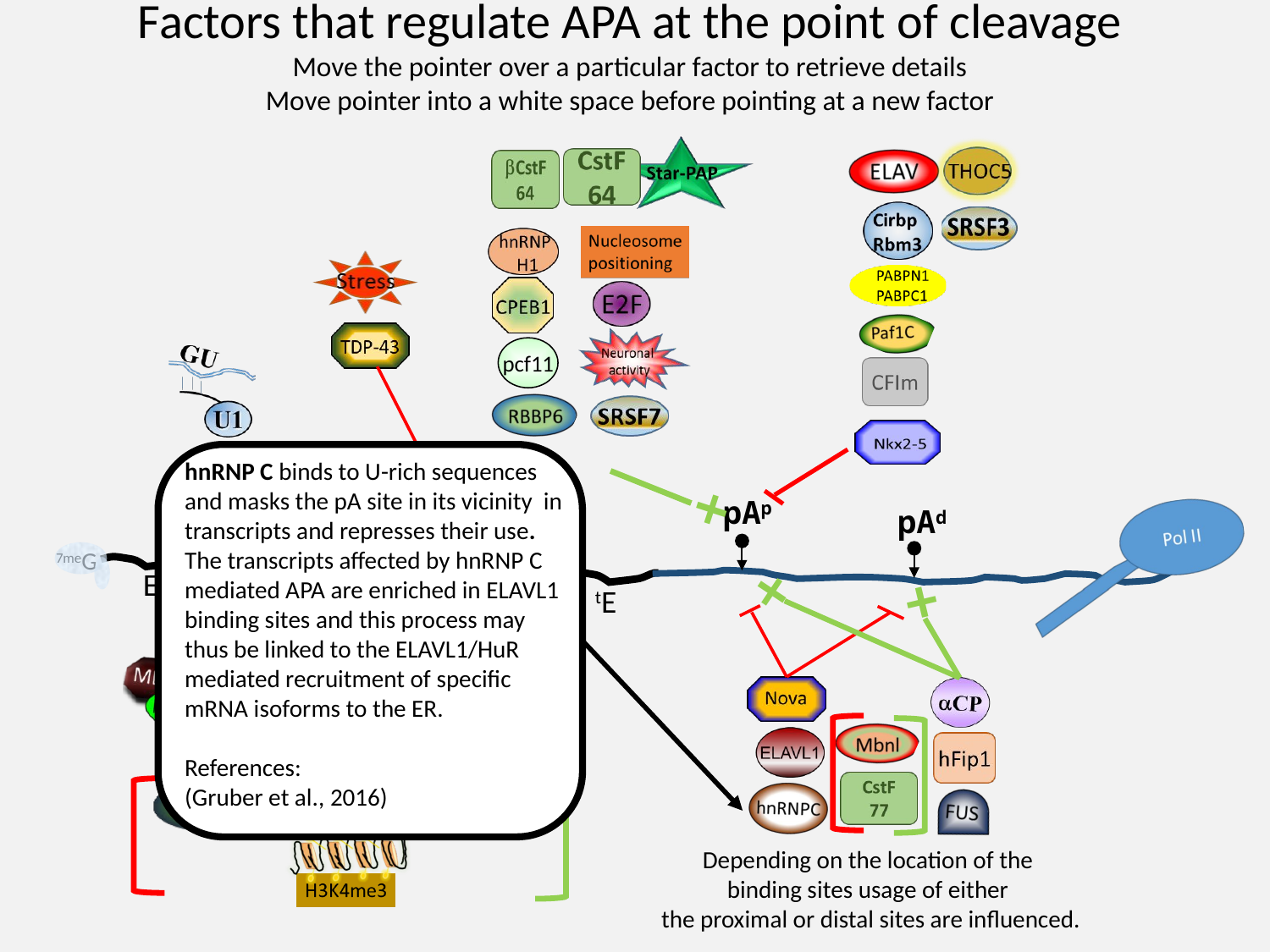

Factors that regulate APA at the point of cleavage
Move the pointer over a particular factor to retrieve details
Move pointer into a white space before pointing at a new factor
pcf11
hnRNP C binds to U-rich sequences and masks the pA site in its vicinity in transcripts and represses their use. The transcripts affected by hnRNP C mediated APA are enriched in ELAVL1 binding sites and this process may thus be linked to the ELAVL1/HuR mediated recruitment of specific mRNA isoforms to the ER.
References:
(Gruber et al., 2016)
+
pAi
pAp
pAd
+
7meG
+
+
i
E
E
i
tE
Depending on the location of the
binding sites usage of either
the proximal or distal sites are influenced.

## Slide 27
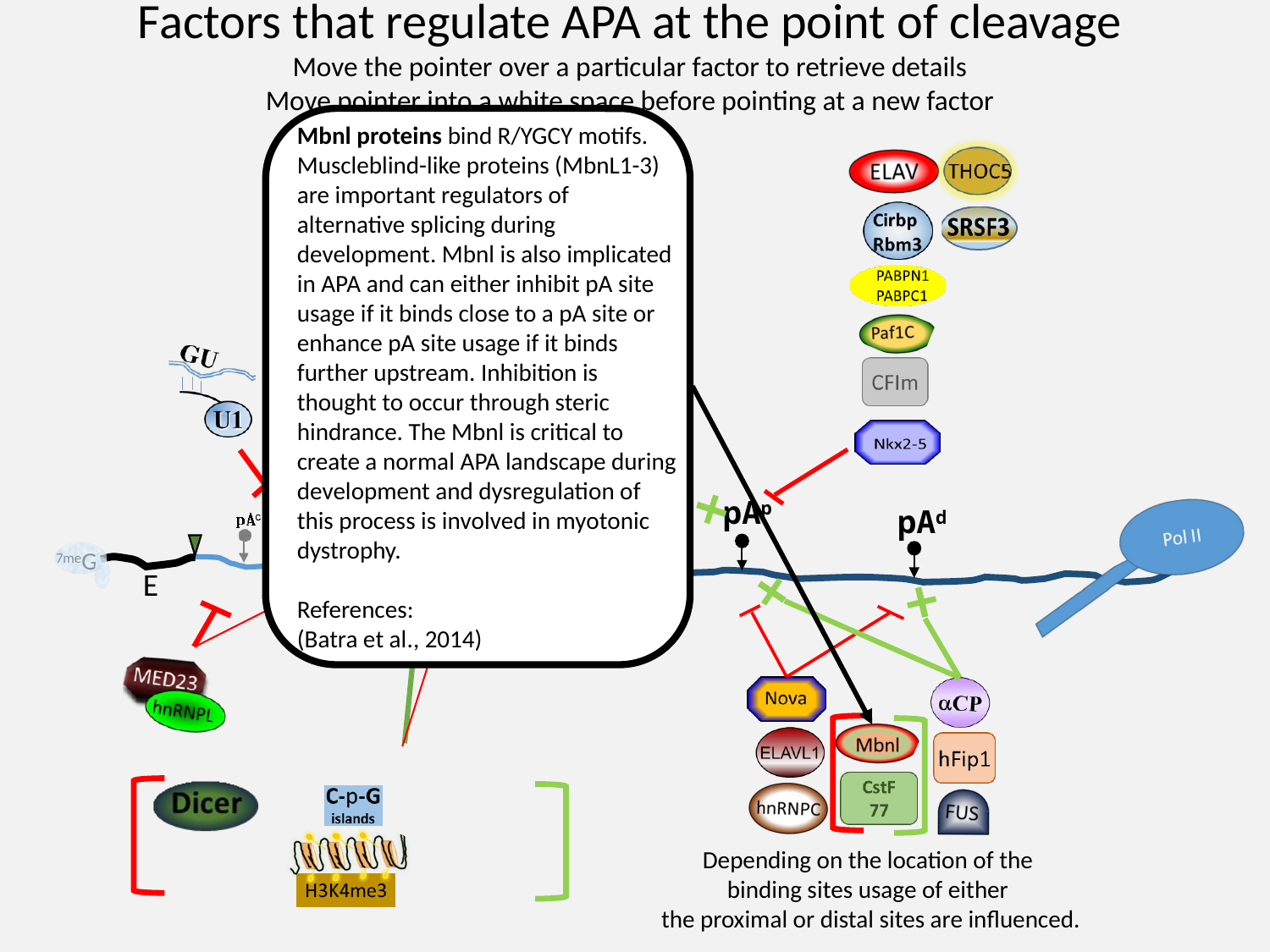

Factors that regulate APA at the point of cleavage
Move the pointer over a particular factor to retrieve details
Move pointer into a white space before pointing at a new factor
Mbnl proteins bind R/YGCY motifs. Muscleblind-like proteins (MbnL1-3) are important regulators of alternative splicing during development. Mbnl is also implicated in APA and can either inhibit pA site usage if it binds close to a pA site or enhance pA site usage if it binds further upstream. Inhibition is thought to occur through steric hindrance. The Mbnl is critical to create a normal APA landscape during development and dysregulation of this process is involved in myotonic dystrophy.
References:
(Batra et al., 2014)
pcf11
+
pAi
pAp
pAd
+
7meG
+
+
i
E
E
i
tE
Depending on the location of the
binding sites usage of either
the proximal or distal sites are influenced.

## Slide 28
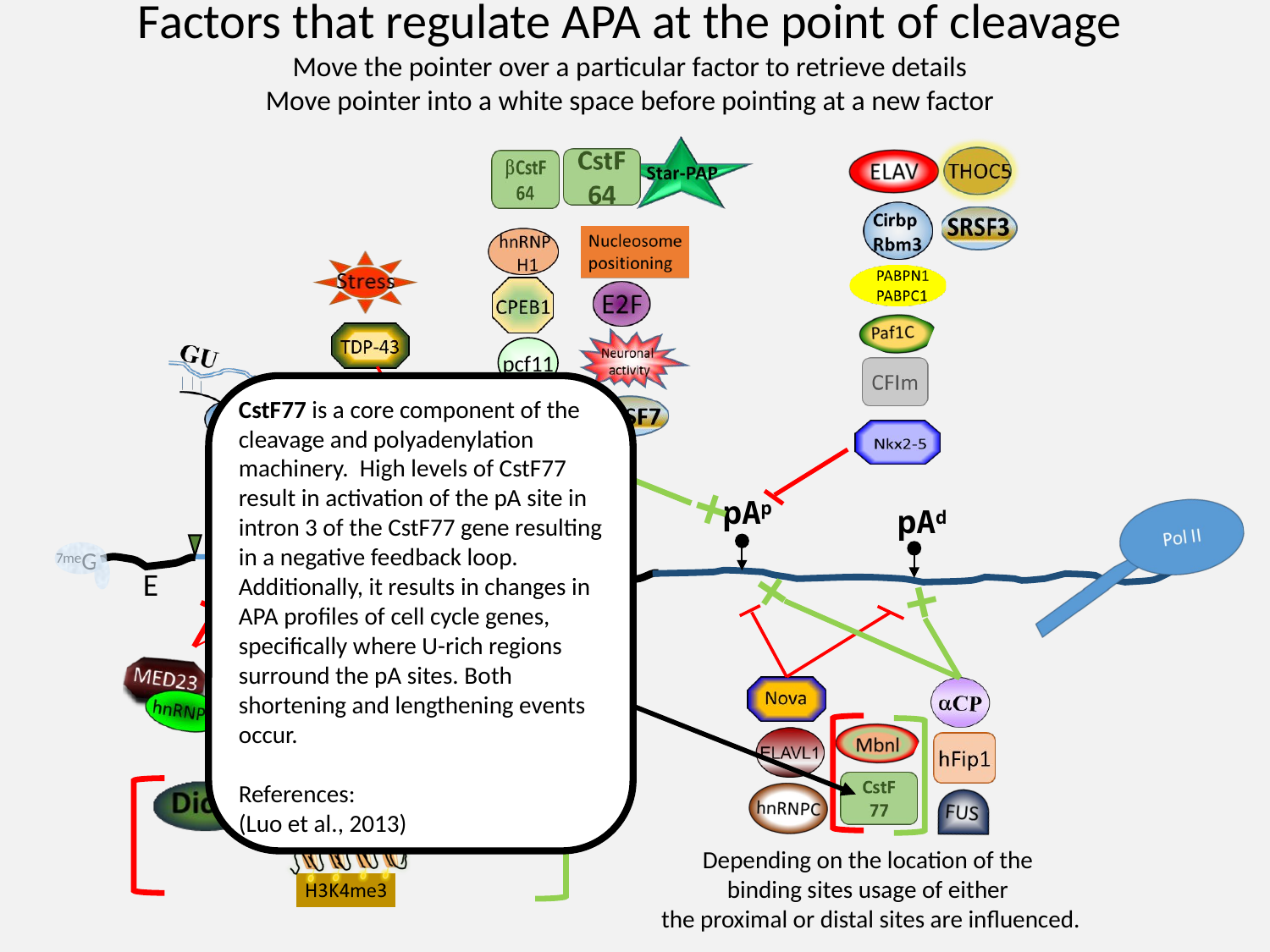

Factors that regulate APA at the point of cleavage
Move the pointer over a particular factor to retrieve details
Move pointer into a white space before pointing at a new factor
pcf11
CstF77 is a core component of the cleavage and polyadenylation machinery. High levels of CstF77 result in activation of the pA site in intron 3 of the CstF77 gene resulting in a negative feedback loop. Additionally, it results in changes in APA profiles of cell cycle genes, specifically where U-rich regions surround the pA sites. Both shortening and lengthening events occur.
References:
(Luo et al., 2013)
+
pAi
pAp
pAd
+
7meG
+
+
i
E
E
i
tE
Depending on the location of the
binding sites usage of either
the proximal or distal sites are influenced.

## Slide 29
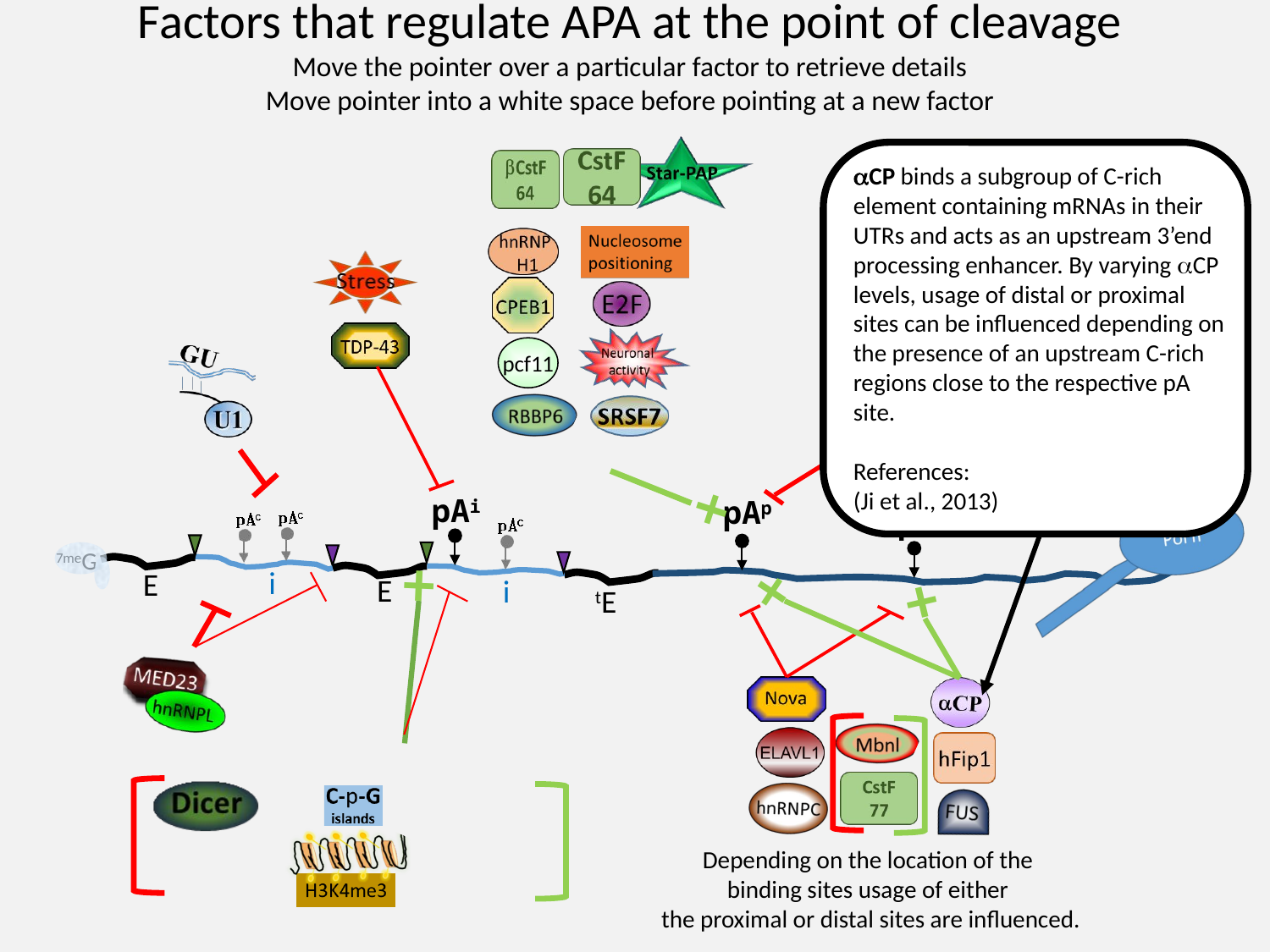

Factors that regulate APA at the point of cleavage
Move the pointer over a particular factor to retrieve details
Move pointer into a white space before pointing at a new factor
aCP binds a subgroup of C-rich element containing mRNAs in their UTRs and acts as an upstream 3’end processing enhancer. By varying aCP levels, usage of distal or proximal sites can be influenced depending on the presence of an upstream C-rich regions close to the respective pA site.
References:
(Ji et al., 2013)
pcf11
+
pAi
pAp
pAd
+
7meG
+
+
i
E
E
i
tE
Depending on the location of the
binding sites usage of either
the proximal or distal sites are influenced.

## Slide 30
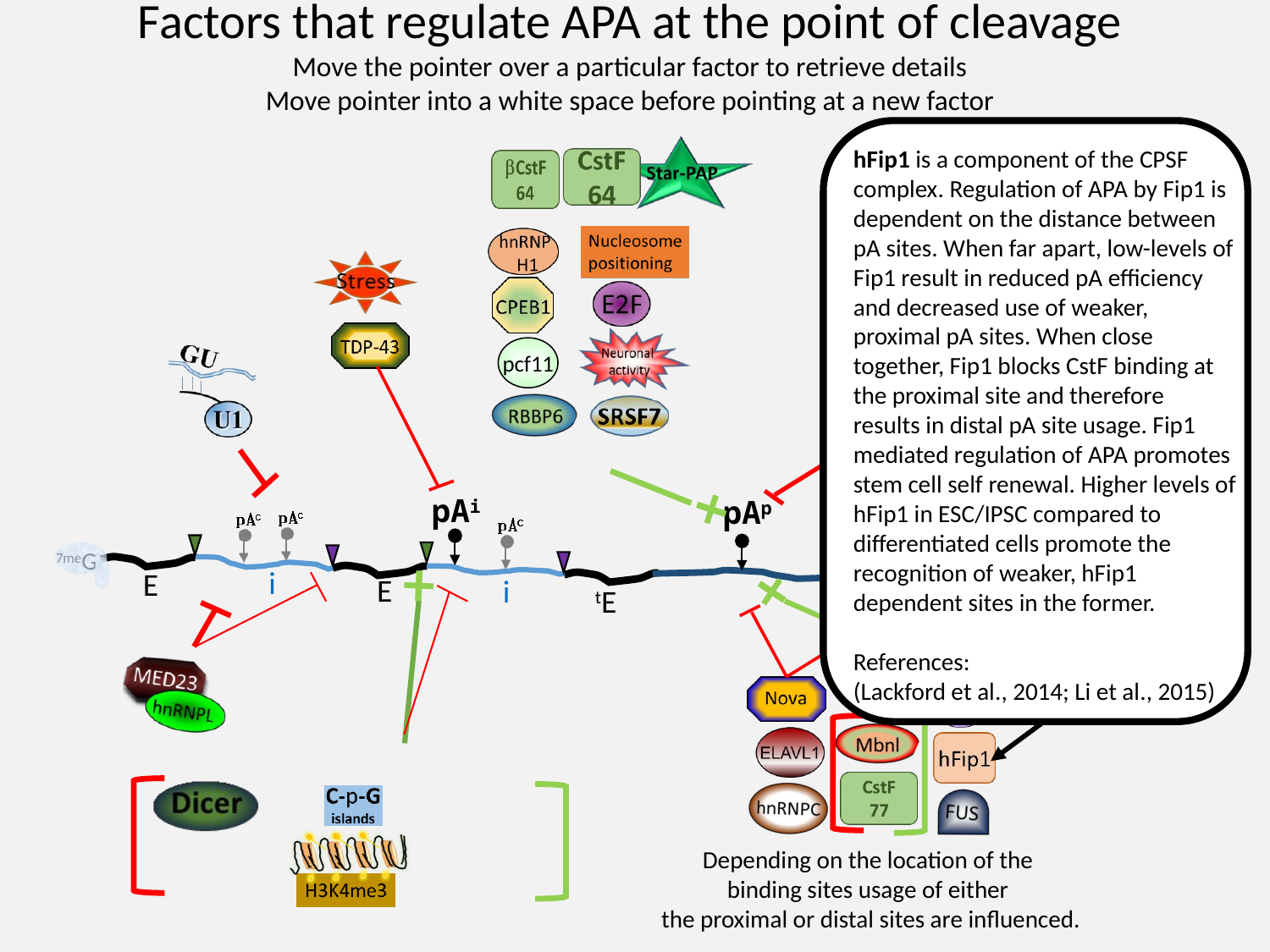

Factors that regulate APA at the point of cleavage
Move the pointer over a particular factor to retrieve details
Move pointer into a white space before pointing at a new factor
hFip1 is a component of the CPSF complex. Regulation of APA by Fip1 is dependent on the distance between pA sites. When far apart, low-levels of Fip1 result in reduced pA efficiency and decreased use of weaker, proximal pA sites. When close together, Fip1 blocks CstF binding at the proximal site and therefore results in distal pA site usage. Fip1 mediated regulation of APA promotes stem cell self renewal. Higher levels of hFip1 in ESC/IPSC compared to differentiated cells promote the recognition of weaker, hFip1 dependent sites in the former.
References:
(Lackford et al., 2014; Li et al., 2015)
pcf11
+
pAi
pAp
pAd
+
7meG
+
+
i
E
E
i
tE
Depending on the location of the
binding sites usage of either
the proximal or distal sites are influenced.

## Slide 31
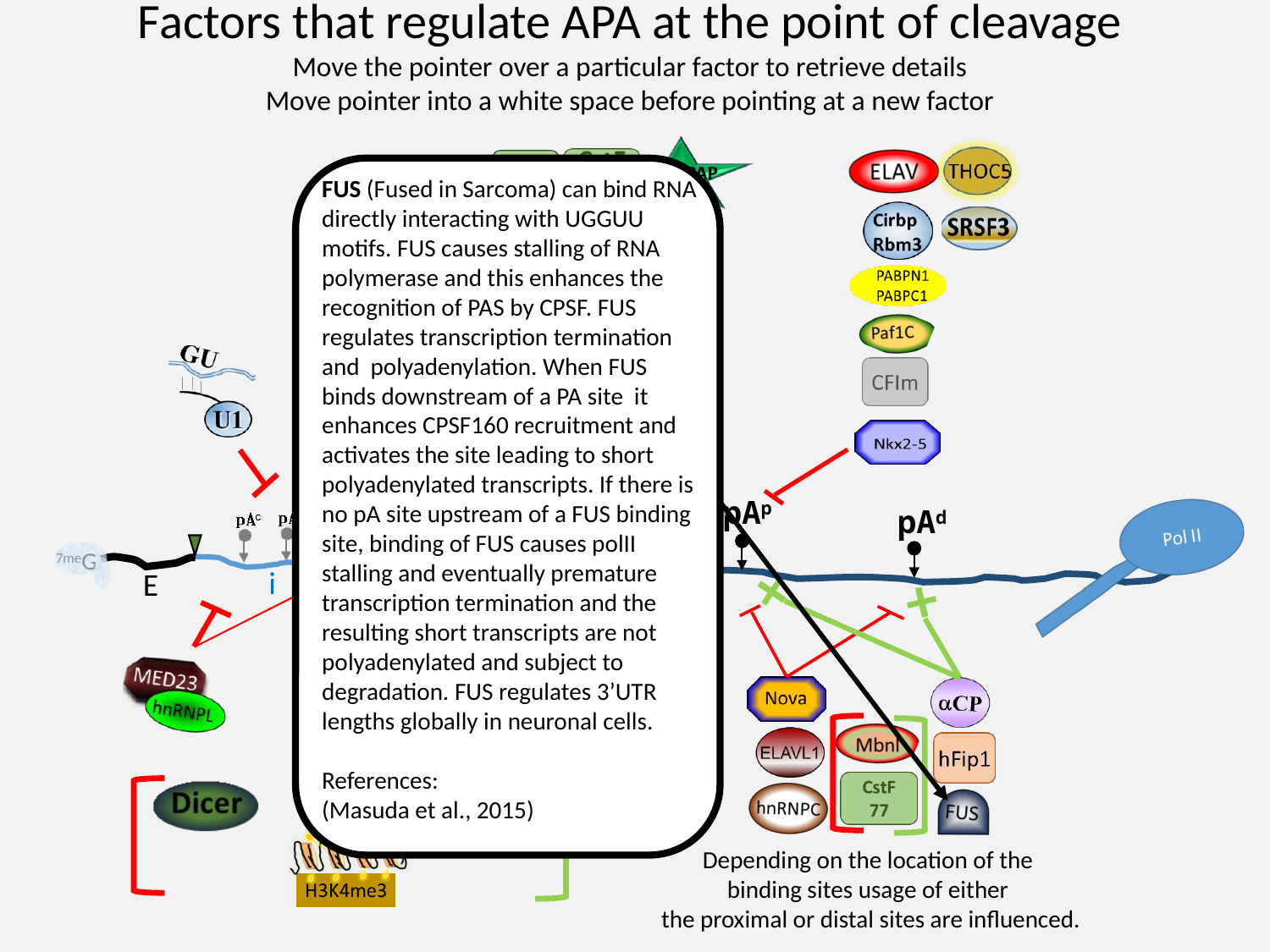

Factors that regulate APA at the point of cleavage
Move the pointer over a particular factor to retrieve details
Move pointer into a white space before pointing at a new factor
FUS (Fused in Sarcoma) can bind RNA directly interacting with UGGUU motifs. FUS causes stalling of RNA polymerase and this enhances the recognition of PAS by CPSF. FUS regulates transcription termination and polyadenylation. When FUS binds downstream of a PA site it enhances CPSF160 recruitment and activates the site leading to short polyadenylated transcripts. If there is no pA site upstream of a FUS binding site, binding of FUS causes polII stalling and eventually premature transcription termination and the resulting short transcripts are not polyadenylated and subject to degradation. FUS regulates 3’UTR lengths globally in neuronal cells.
References:
(Masuda et al., 2015)
pcf11
+
pAi
pAp
pAd
+
7meG
+
+
i
E
E
i
tE
Depending on the location of the
binding sites usage of either
the proximal or distal sites are influenced.

## Slide 32
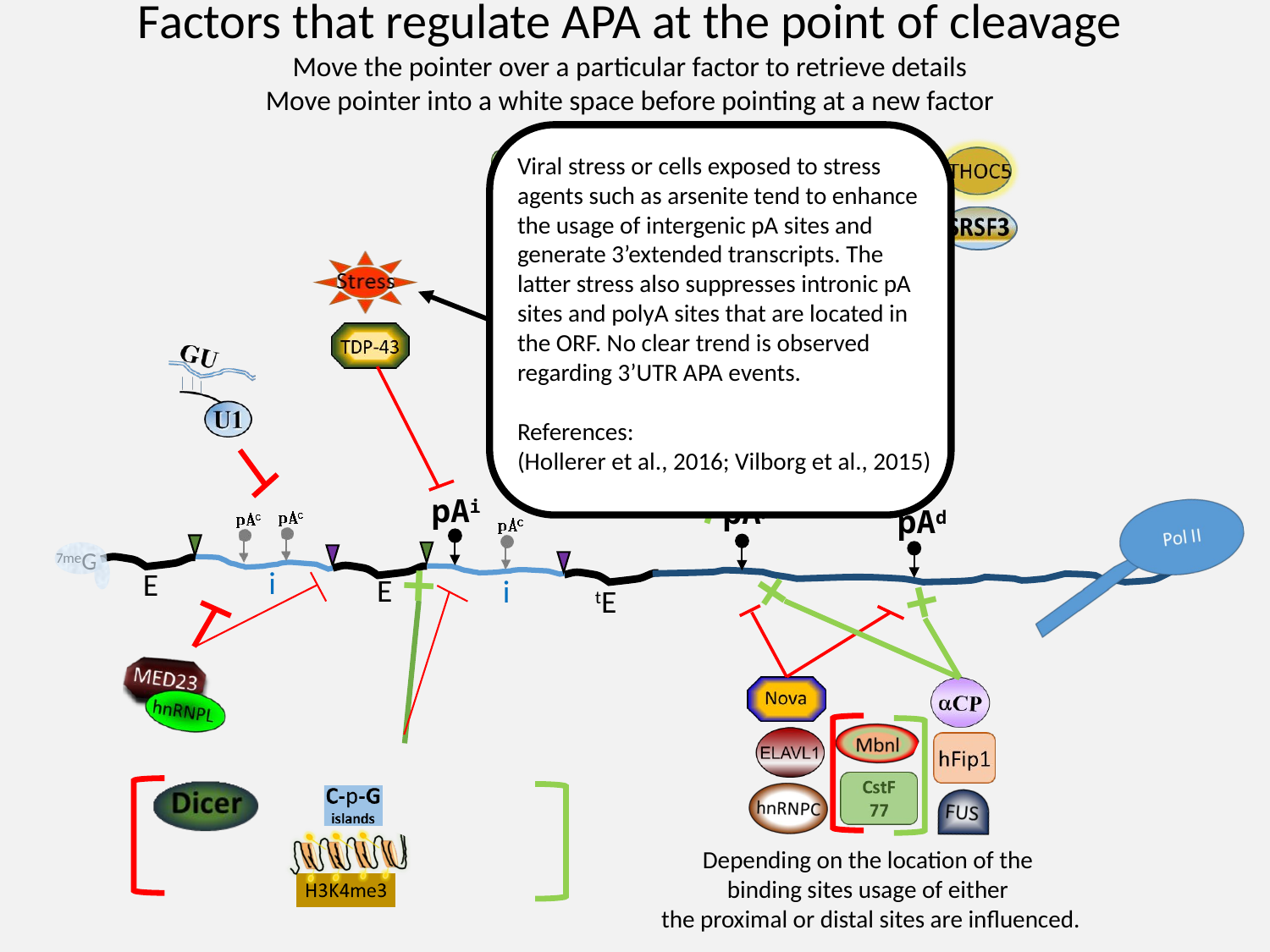

Factors that regulate APA at the point of cleavage
Move the pointer over a particular factor to retrieve details
Move pointer into a white space before pointing at a new factor
Viral stress or cells exposed to stress agents such as arsenite tend to enhance the usage of intergenic pA sites and generate 3’extended transcripts. The latter stress also suppresses intronic pA sites and polyA sites that are located in the ORF. No clear trend is observed regarding 3’UTR APA events.
References:
(Hollerer et al., 2016; Vilborg et al., 2015)
pcf11
+
pAi
pAp
pAd
+
7meG
+
+
i
E
E
i
tE
Depending on the location of the
binding sites usage of either
the proximal or distal sites are influenced.

## Slide 33
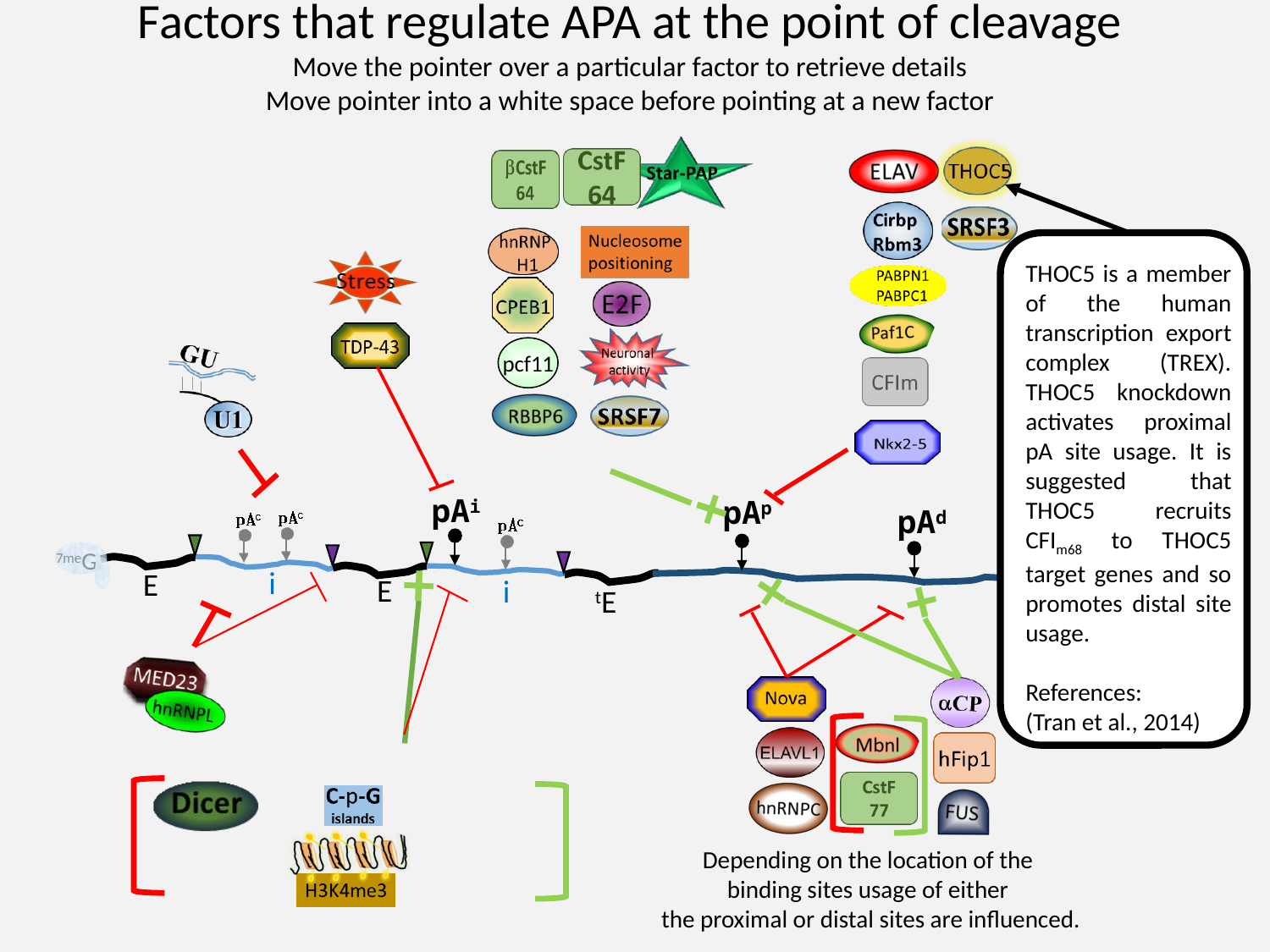

Factors that regulate APA at the point of cleavage
Move the pointer over a particular factor to retrieve details
Move pointer into a white space before pointing at a new factor
THOC5 is a member of the human transcription export complex (TREX). THOC5 knockdown activates proximal pA site usage. It is suggested that THOC5 recruits CFIm68 to THOC5 target genes and so promotes distal site usage.
References:
(Tran et al., 2014)
pcf11
+
pAi
pAp
pAd
+
7meG
+
+
i
E
E
i
tE
Depending on the location of the
binding sites usage of either
the proximal or distal sites are influenced.

## Slide 34
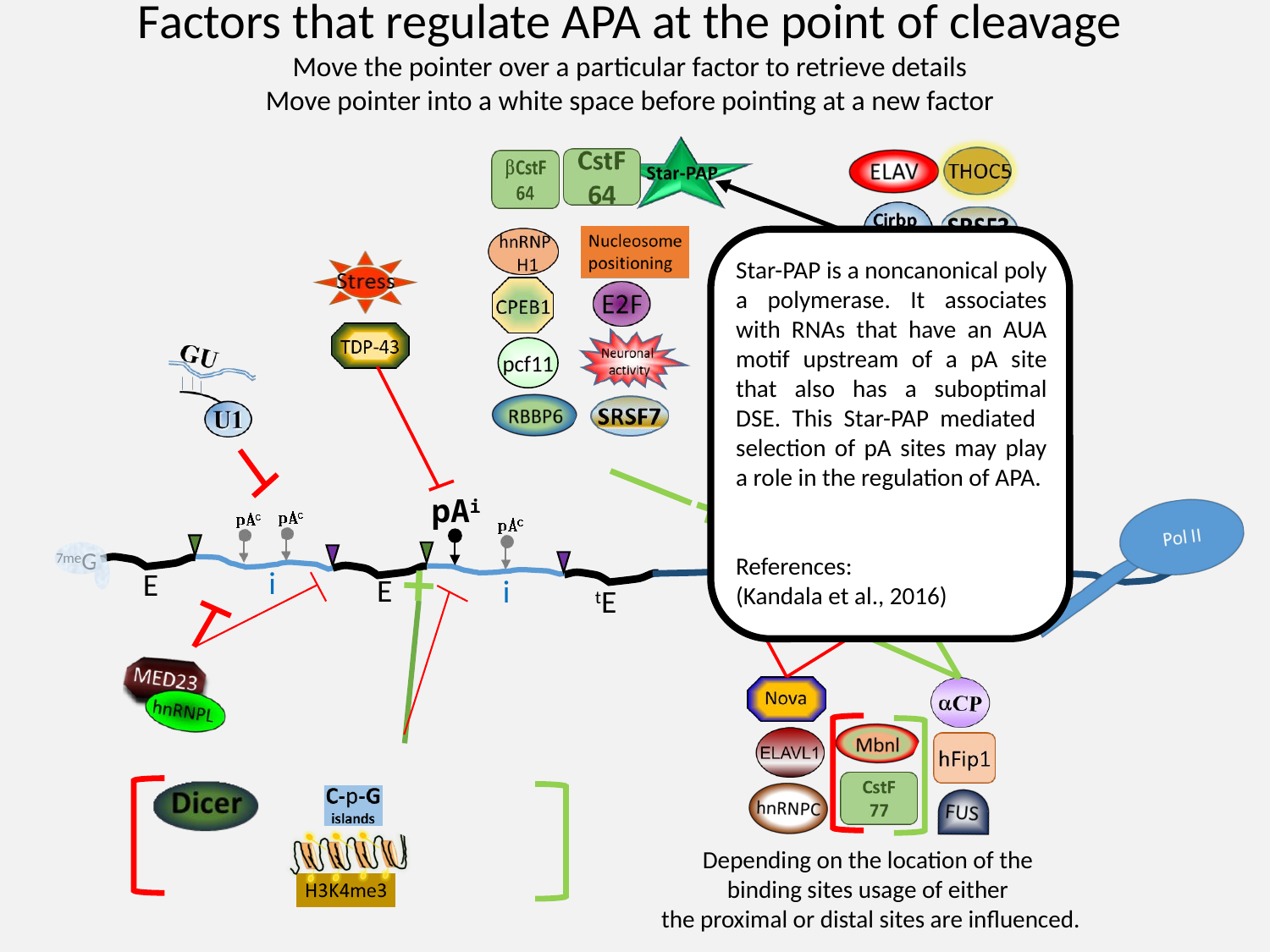

Factors that regulate APA at the point of cleavage
Move the pointer over a particular factor to retrieve details
Move pointer into a white space before pointing at a new factor
Star-PAP is a noncanonical poly a polymerase. It associates with RNAs that have an AUA motif upstream of a pA site that also has a suboptimal DSE. This Star-PAP mediated selection of pA sites may play a role in the regulation of APA.
References:
(Kandala et al., 2016)
pcf11
+
pAi
pAp
pAd
+
7meG
+
+
i
E
E
i
tE
Depending on the location of the
binding sites usage of either
the proximal or distal sites are influenced.

## Slide 35
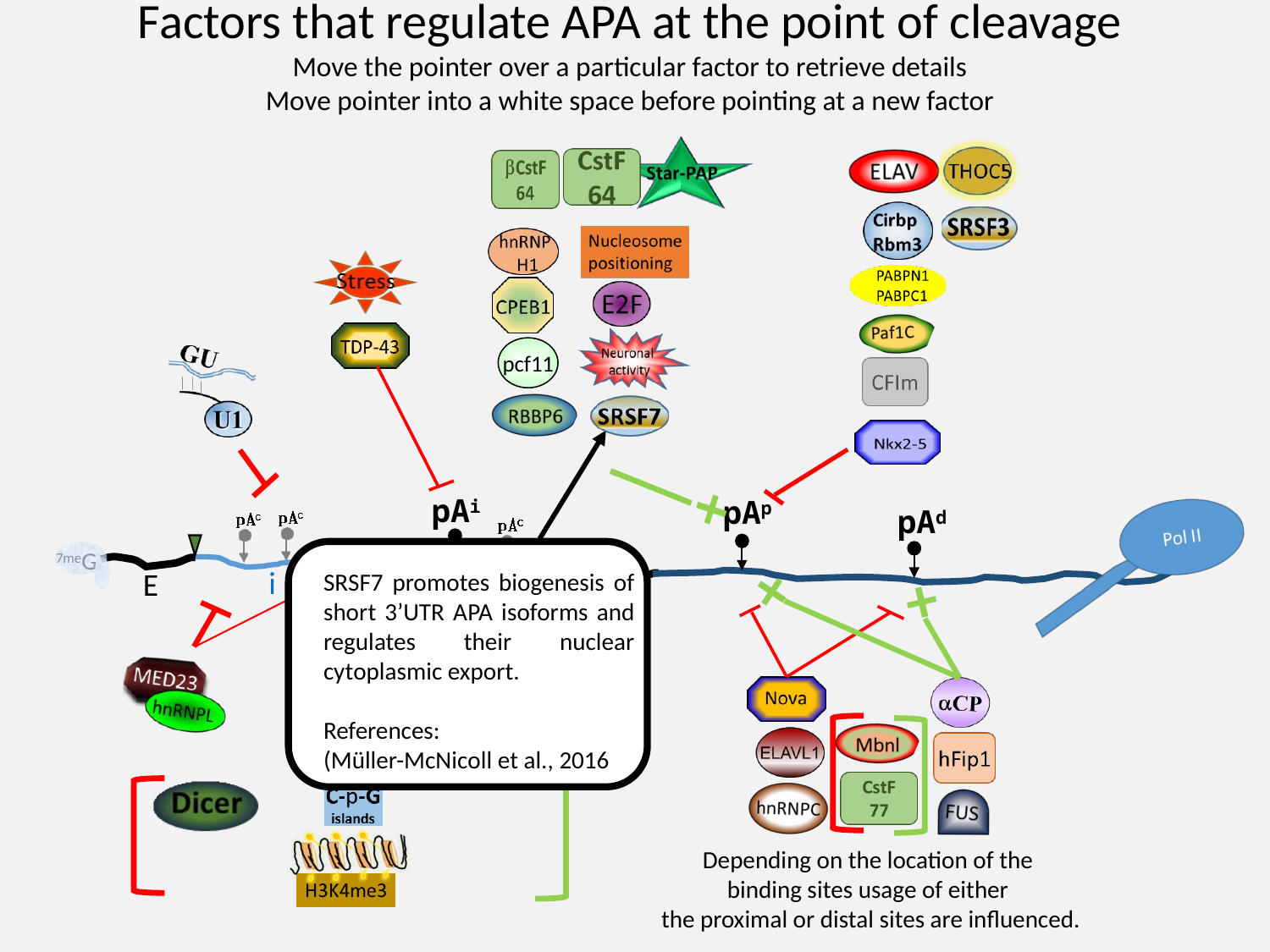

Factors that regulate APA at the point of cleavage
Move the pointer over a particular factor to retrieve details
Move pointer into a white space before pointing at a new factor
pcf11
+
pAi
pAp
pAd
+
7meG
+
+
i
E
SRSF7 promotes biogenesis of short 3’UTR APA isoforms and regulates their nuclear cytoplasmic export.
References:
(Müller-McNicoll et al., 2016
E
i
tE
Depending on the location of the
binding sites usage of either
the proximal or distal sites are influenced.

## Slide 36
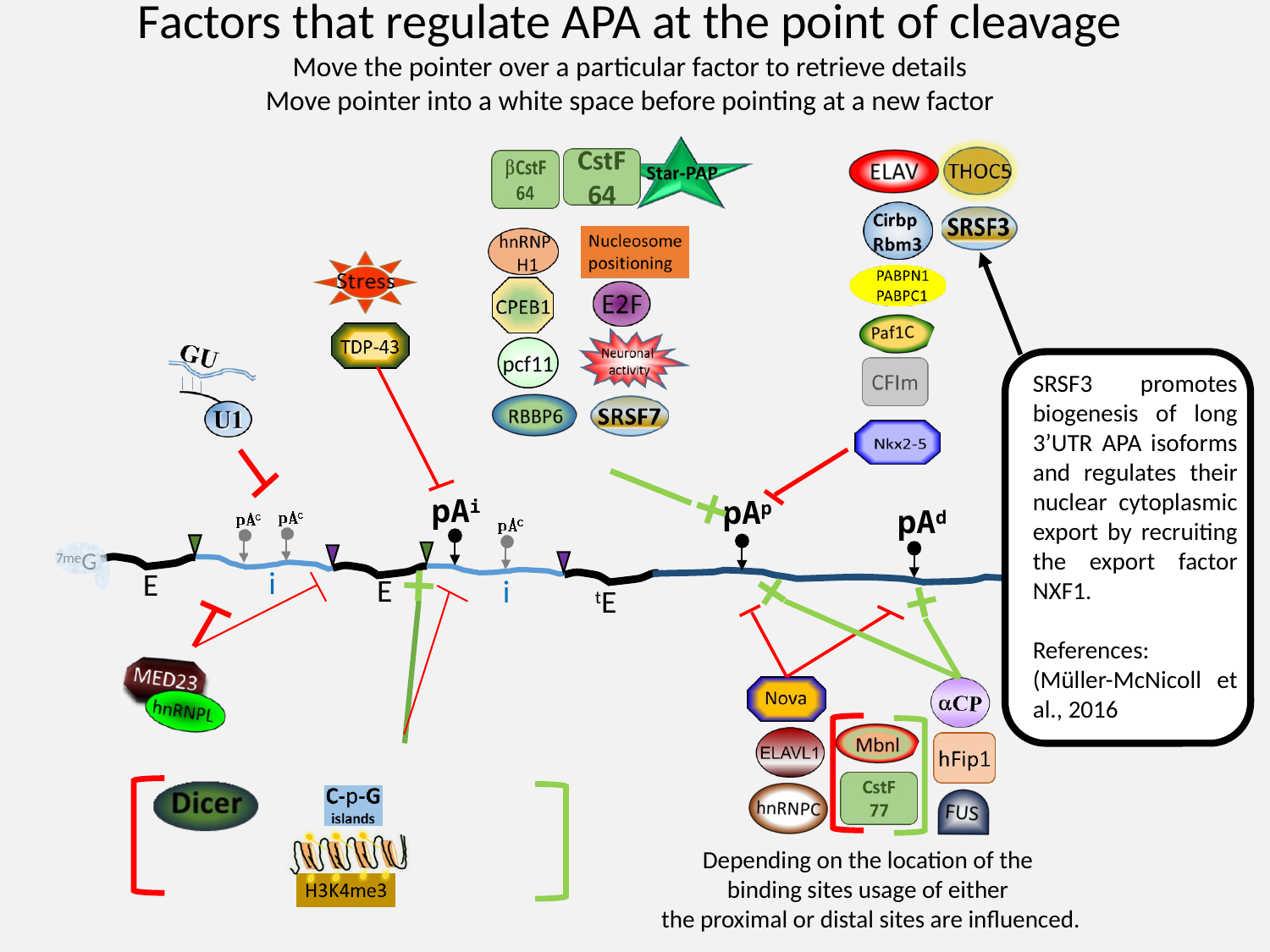

Factors that regulate APA at the point of cleavage
Move the pointer over a particular factor to retrieve details
Move pointer into a white space before pointing at a new factor
pcf11
SRSF3 promotes biogenesis of long 3’UTR APA isoforms and regulates their nuclear cytoplasmic export by recruiting the export factor NXF1.
References:
(Müller-McNicoll et al., 2016
+
pAi
pAp
pAd
+
7meG
+
+
i
E
E
i
tE
Depending on the location of the
binding sites usage of either
the proximal or distal sites are influenced.
